# YAP1 as a Novel Negative Biomarker of Immune Checkpoint Inhibitors for EGFR-Mutant Non-Small-Cell Lung Cancer

**DOI:** 10.1155/2023/4689004

**Published:** 2023-06-21

**Authors:** Ling-Chen Li, Xie-Wan Chen, Ling Fang, Chun-Li Jian, Yong-Xin Yu, Xing-Yun Liao, Jian-Guo Sun

**Affiliations:** ^1^Cancer Institute, Xinqiao Hospital, Army Medical University, Chongqing 400037, China; ^2^Medical English Department, College of Basic Medicine, Army Medical University, Chongqing 400038, China; ^3^Department of Medical Oncology, Cancer Hospital, Chongqing University, Chongqing 400030, China

## Abstract

**Background:**

Immune checkpoint inhibitors (ICIs) have become a standard care in non-small-cell lung cancer (NSCLC). However, its application to epidermal growth factor receptor (EGFR)-mutant NSCLC patients is confronted with drug resistance. This study aimed to clarify the potential role of Yes1-associated transcriptional regulator (YAP1) in ICIs treatment for EGFR-mutant NSCLC population.

**Methods:**

All the clinical data of NSCLC were downloaded from Cancer Genome Atlas (TCGA) and Gene Expression Omnibus (GEO) for GSE11969 and GSE72094. Based on YAP1 expression, all the NSCLC patients including the EGFR-mutant and EGFR-wildtype (WT) patients were divided into two groups, YAP1_High and YAP1_Low. Using cBioPortal, genetic alterations were analyzed for identification of immunogenicity in EGFR-mutant NSCLC. MR analysis was used to analyze the hub gene of EGFR. The infiltration of immune cells and the expression of the identified tumor-associated antigens were identified with TIMER. By graph learning-based dimensionality reduction analysis, the immune landscape was visualized. Moreover, survival analysis was performed to verify the predictive value of YAP1 in ICIs treatment for EGFR-mutant NSCLC population using Ren's research data (NCT03513666).

**Results:**

YAP1 was a poor prognostic factor of EGFR-mutant NSCLC population rather than lung adenocarcinoma (LUAD) patients. MR analysis revealed that the EGFR gene regulated YAP1 expression. YAP1 was identified as a hub gene closely associated with immunosuppressive microenvironment and poor prognosis in EGFR-mutant NSCLC population in TCGA LUAD. Tumors with YAP1_High showed an immune-“cold” and immunosuppressive phenotype, whereas those with YAP1_Low demonstrated an immune-“hot” and immunoactive phenotype. More importantly, it was verified that YAP1_High subpopulation had a significantly shorter progression-free survival (PFS) and overall survival (OS) after ICIs treatment in EGFR-mutant NSCLC patients in the clinical trial.

**Conclusions:**

YAP1 mediates immunosuppressive microenvironment and poor prognosis in EGFR-mutant NSCLC population. YAP1 is a novel negative biomarker of ICIs treatment in EGFR-mutant NSCLC population. *Clinical Trials*. This trial is registered with NCT03513666.

## 1. Introduction

Lung cancer is still the leading cause of cancer death worldwide [1, 2], among which non-small-cell lung cancer (NSCLC) is the most common type. Despite great progress in treatment over the last decade, the mortality remains high, and the 5-year survival rate is approximately 15% in advanced NSCLC patients [3]. Encouragingly, the immunotherapy for NSCLC has been revolutionized by the introduction of immune checkpoint inhibitors (ICIs) against programmed death protein-1 (PD-1) and its ligand PD-L1 or cytotoxic T-lymphocyte antigen-4 (CTLA-4) [4–6]. Increasing studies have indicated that ICIs treatment has promising efficacy since it can greatly prolong the survival of patients with advanced NSCLC including lung adenocarcinoma (LUAD) and lung squamous carcinoma (LUSC) [7]. Application of ICIs treatment in advanced NSCLC patients has thus become a research hotspot and standard care, especially in those patients without epidermal growth factor receptor (EGFR) mutation [8]. However, those EGFR-mutant patients appeared to respond poorly to ICIs treatment [9]. The reasons for different effects of ICIs treatment on EGFR-mutant and wild type (WT) NSCLC patients are unknown. Thus, we intended to explore some clues from gene regulation and immune microenvironment.

At present, PD-L1 is widely used as a biomarker indicating sensitivity to ICIs treatment in NSCLC [10]. However, not all PD-L1-positive NSCLC patients can benefit from ICIs treatment [11]. In addition, tumor mutation burden (TMB) and microsatellite instability (MSI) are widely used as major clinical indicators for ICIs treatment in pan-cancer. However, TMB or MSI cannot fully predict the response of NSCLC to ICIs treatment [12–15]. Nevertheless, ICIs treatment has greatly improved the outcomes of NSCLC patients without EGFR mutation.

As for EGFR-mutant NSCLC patients, tyrosine kinase inhibitors (TKIs) are milestones of tumor-targeted therapy, which have greatly improved the outcomes. However, acquired resistance to EGFR-TKIs remains a clinical challenge. Despite a report demonstrating clinical benefits of ICIs monotherapy or ICIs combination with chemotherapy for some patients with EGFR-TKIs resistance [16], it is still controversial which subpopulation with EGFR-TKIs resistance is suitable for ICIs. Many factors may account for the insensitivity of NSCLC patients with EGFR mutation to ICIs treatment, but undisputed biomarkers are still lacking. Hence, to improve the curative effect in those patients with EGFR mutation, it is necessary to explore a novel biomarker to guide ICIs treatment in clinical work.

Our team previously found that the Yes1-associated transcriptional regulator (YAP1) was associated with EGFR-TKI resistance [17]. Here, we identified YAP1 as a hub gene that was closely associated with EGFR regulation in NSCLC patients. Dividing the EGFR-mutant NSCLC patients into two subpopulations based on YAP1 expression, we found that patients with YAP1_High tumor had immunosuppressive immune cells and inferior prognosis in EGFR-mutant NSCLC patients compared to those with YAP1_Low tumor. Moreover, YAP1_High patients had poor progression-free survival (PFS) and overall survival (OS) after ICIs treatment in EGFR-mutant NSCLC population. Thus, our findings might provide scientists and oncology clinicians with a valuable and reliable biomarker for selection of patients suitable for ICIs treatment from EGFR-mutant NSCLC population.

## 2. Methods

### 2.1. Data Acquisition

The LUAD dataset required for the study was downloaded from the Cancer Genome Atlas (TCGA, https://portal.gdc.Cancer.gov/), including raw counts of RNA sequencing data of the tumor transcriptome with their clinical information. There were 513 cases of tumor tissues. Mutation information from the TCGA cohort was collected from the UCSC Xena database (https://xenabrowser.net/datapages/), excluding the patients without survival information. Finally, 444 EGFR-WT and 65 EGFR-mutant NSCLC cases were obtained. GSE11969, GSE72094, GSE31210, and GSE13522 were obtained from the Gene Expression Omnibus (GEO) database (https://www.ncbi.nlm.nih.gov/GEO/). The data of 149 NSCLC patients were collected from GSE11969, including 90 LUAD patients and 34 EGFR-mutant patients. The data of 442 LUAD patients were obtained from GSE72094, including 47 EGFR-mutant cases. The expression profiles of 226 cases of LUAD were collected from GSE31210, including 127 EGFR-mutant cases. A total of 27 EGFR-WT LUAD patients having received ICIs treatment were from GSE13522. The mutation frequency was analyzed by the cbioportal (https://www.cbioportal.org/). All the detailed dataset information including the LUAD dataset, GSE11969, GSE72094, GSE31210, GSE13522, immune infiltration, mutation enrichment, GO-KEGG, and the phase-II trial from Prof. Ren's lab (NCT03513666) are shown in Supplementary Table 1. The progression-free survival (PFS), overall survival (OS), and the expression of YAP1 were included. The immune infiltration of B cells, CD8^+^/CD4^+^ T cells, Tregs, NK cells, macrophages M1/M2, and dendritic cells and the term of gene ontology-Kyoto Encyclopedia of Genes and Genomes (GO-KEGG) as well as mutation enrichment are also provided in Supplementary Table 1.

### 2.2. MR Analysis and PCA Analysis

The MR graph (https://www.mr-graph.org/) was used to establish the interaction network between different main regulatory proteins and their upstream driving genes in different TCGA datasets. Principal component (PC) analysis was used to visualize the data in two dimensions with the “ggplot2” packages.

### 2.3. Functional Enrichment Analysis

The selected patients were divided into two groups based on YAP1 expression. By using a bioinformatics online tool (https://www.bioinformatics.com.cn), (GO) including biological process (BP), cellular components (CC), molecular function (MF), and (KEGG) pathway enrichment analyses were performed. ^*∗*^*P* < 0.05 was considered statistically significant.

### 2.4. Survival Analysis

Kaplan–Meier analysis of OS and PFS) was performed using Survminer [18] (R package). We calculated the hazard ratio (HR) and log-rank *P* value of the 95% confidence interval. All codes used for the analyses were written in R software (3.6.1).

### 2.5. Relative Proportions of Immune Cells in NSCLC

Based on normalized gene expression data from the TCGA LUAD, we estimated a subset of 22 tumor-infiltrating immune cells (TIICs) in LUAD tissue using the CIBERSORT (https://cibersort.stanford.edu/) computational algorithm [19] and inferred the relative proportions of the 22 TIIC subtypes. Only patients with CIBERSORT *P* < 0.05 were considered eligible for further analysis. By using the Wilcoxon rank sum test, TIIC proportions were analyzed between low and high expression groups based on prognosis-related gene expression in LUAD patients. The single-sample GSEA method of “GSVA” (R package) [20] was used, including 22 types of immune cells, to analyze the level of immune cell infiltration of LUAD expression profile data and evaluate the correlation of prognostic genes with these immune cells using Spearman analysis. *P* < 0.05 was considered statistically significant.

### 2.6. Statistical Analysis

The expression levels of hub genes between mutation and wild-type groups were assessed and compared using *t*-test. Statistical significance was set at ^*∗*^*P* < 0.05. Pearson analysis was used to evaluate the correlation between prognosis-related genes and immune cells. The value of correlation strength >0.2 was considered significant.

## 3. Results

### 3.1. YAP1 Is Not a Prognostic Factor of LUAD

**Figure 1:**
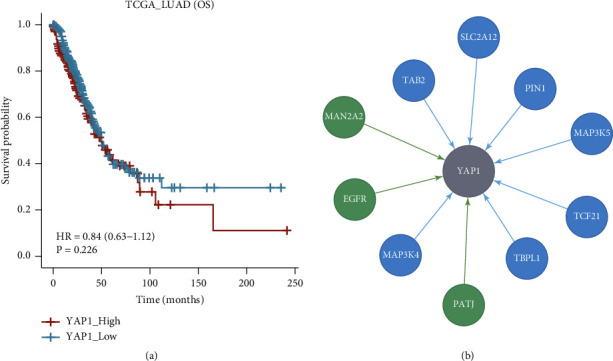
YAP1 is not a prognostic factor of LUAD. (a) The OS had no significant difference between YAP1_High and YAP1_Low lung cancer patients from TCGA LUAD. (b) The EGFR gene regulated YAP1 expression as shown by MR analysis.

Our previous work has confirmed that YAP1 regulated the stemness and promoted the growth of tumor cells [21], and it was closely related to EGFR-TKI resistance [17]. Thus, we tried to observe the effect of YAP1 on ICIs treatment for NSCLC. First, all the LUAD patients from TCGA LUAD were divided into two subpopulations according to YAP1 expression (YAP1_High vs YAP1_Low = 4 : 6). Second, by analyzing the information of LUAD patients from TCGA LUAD, we found that the OS had no significant difference between YAP1_High and YAP1_Low LUAD patients (46 months vs 51 months, *P*=0.226, Figure 1(a)). Next, MR analysis revealed that the EGFR gene regulated YAP1 expression (Figure 1(b)). Together, the results indicate that YAP1 has no effect on the prognosis of LUAD.

### 3.2. Identification of Immunogenicity in EGFR-Mutant NSCLC

**Figure 2:**
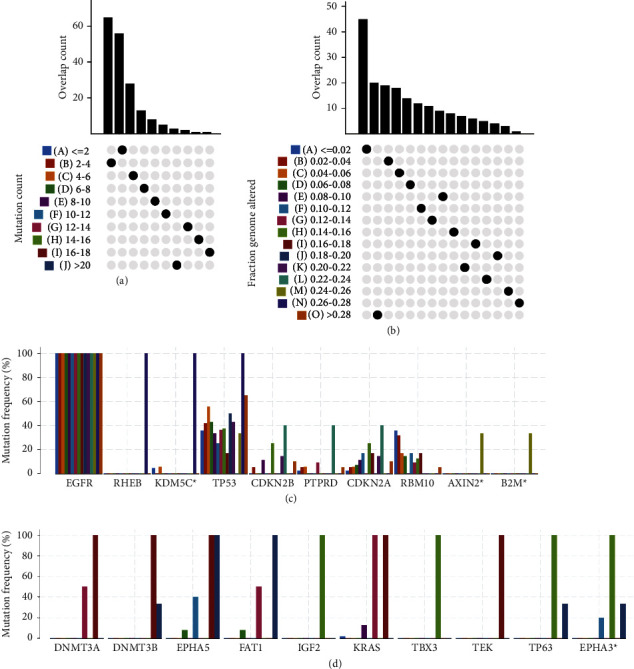
Identification of low immunogenicity in EGFR-mutant NSCLC. Overlapping mutant genes distributed in the mutation count group (a) and fraction genome altered group (b). Genes with the highest frequency in the fraction genome altered groups (c) and mutation count groups (d).

A total of 925 mutant genes encoding tumor-specific antigens were screened by assessing fraction genome alteration and mutation counts in each EGFR-mutant NSCLC patient. Genes with more than 4 mutations accounted for approximately 30% of all mutated genes (Figure 2(a)). Similarly, the majority of genes had a percentage of genomic mutations less than 0.08, and only a small proportion of genes had a large percentage of genomic mutations (Figure 2(b)). Genes with the highest alteration frequency in the fraction genome altered group, including tumor protein *p*53 (TP53), Ras homolog, mTORC1 binding (RHEB), and lysine demethylase 5C (KDM5C), were individually displayed (Figure 2(c)). Genes with the highest mutation frequency in the mutation count group, including DNA methyltransferase 3 alpha (DNMT3A), DNA methyltransferase 3 beta (DNMT3B), EPH receptor A5 (EPHA5), FAT atypical cadherin 1 (FAT1), insulin like growth factor 2 (IGF2), KRAS, T-box transcription factor 3 (TBX3), TEK, TP63, and EPH receptor A3 (EPHA3), were individually displayed (Figure 2(d)). Together, the results indicate the low immunogenicity of EGFR-mutant NSCLC.

### 3.3. YAP1 Is Associated with the Poor Prognosis of EGFR-Mutant NSCLC Patients

**Figure 3:**
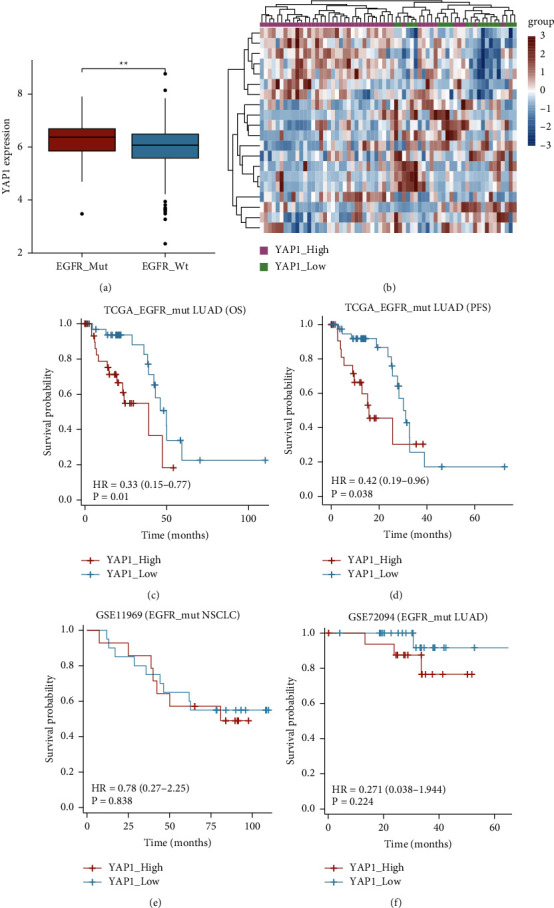
YAP1 is associated with poor prognosis of EGFR-mutant NSCLC patients. (a) The expression of YAP1 in the EGFR mutation group and EGFR-WT group. (b) The heatmap shows the differential expressions of YAP1 in the YAP1_High and YAP1_Low groups in EGFR-mutant NSCLC. (c-d) Kaplan–Meier analysis of the OS and PFS of the YAP1_High and YAP1_Low groups from TCGA LUAD. (e-f) Kaplan–Meier analysis of the OS of the two groups for GSE11969 and GSE72094.

We tried to determine the potential biomarkers and immune subtypes of NSCLC for selection of patients suitable for ICIs therapy from EGFR-mutant population. The expression of YAP1 in the EGFR mutation group was higher than that in the EGFR-WT group (Figure 3(a)). Analyzing the 65 LUAD patients with EGFR mutation from TCGA LUAD, we assigned all the patients into the YAP1_High or YAP1_Low groups (4 : 6) and found that the gene profiles of the two subtypes were obviously different (Figure 3(b)). Analysis of the survival relevance of YAP1 showed that YAP1_High subpopulation had significantly shorter OS (39.1 months vs 49.7 months, *P*=0.01, Figure 3(c)) and PFS (18.7 months vs 31.5 months, *P*=0.038, Figure 3(d)) than the YAP1_Low group.

Similarly, survival analysis was repeated from 32 to 29 EGFR-mutant NSCLC patients for GSE11969 and GSE72094. As a result, the OS of the YAP1_High group was 80.9 months while not reached (NR) in the YAP1_Low group in GSE11969 (*P*=0.838, Figure 3(e)). The OS curves of both groups were separated in GSE72094 (NR vs NR, *P*=0.224, Figure 3(f)). Taken together, YAP1 is associated with poor prognosis of EGFR-mutant NSCLC population both in TCGA LUAD and GEO datasets.

### 3.4. YAP1 Is Associated with Immunosuppressive Microenvironment in EGFR-Mutant NSCLC

**Figure 4:**
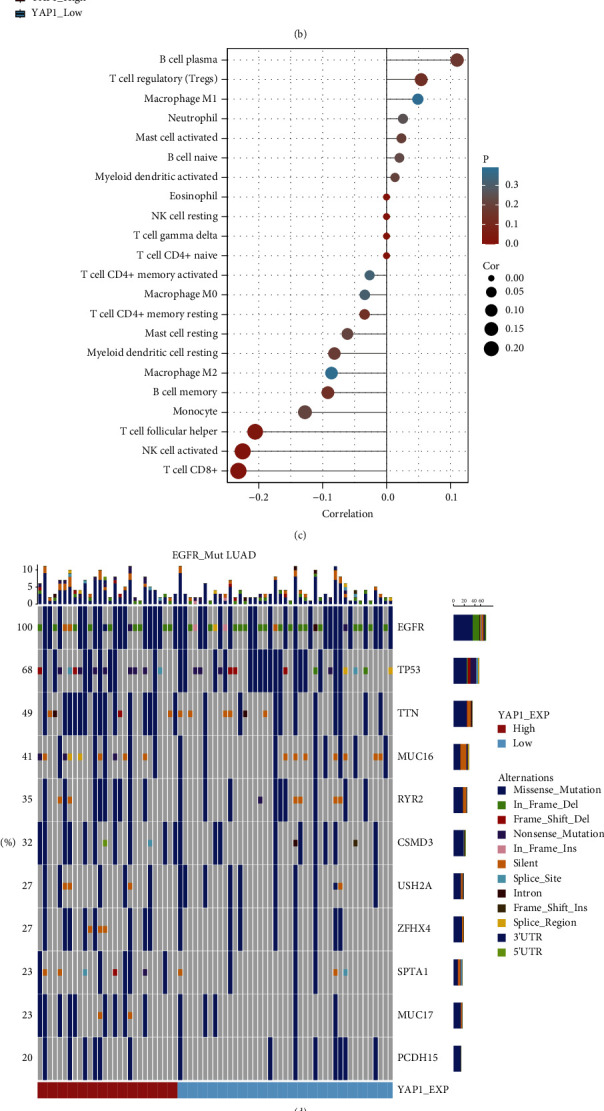
YAP1 is associated with negative tumor microenvironment in EGFR-mutant NSCLC. Spearman's analysis showed infiltration of 22 immune cell types in NSCLC (a) and EGFR-mutant NSCLC (b) using CIBERSORT in TCGA LUAD. (c) The correlation between YAP1 expression and immune cells. (d) The mutations per coding region were evaluated in the two subtypes. (e) The tumor mutation burden (TMB) expression in the YAP1_High and YAP1_Low groups. YAP1 was negatively correlated with PD-L1 (CD274) in EGFR-mutant TCGA LUAD (f) and GSE31210 (g).

Infiltration of TIICs was assessed using ssGSEA and CIBERSORT tools. First, we found that the suppressive immune cells including macrophage M2 and activated mast cells were highly expressed in the EGFR-mutant group. The active immune cells including NK cells, CD8^+^ T cells, and T-helper cells were highly expressed in the EGFR-WT group for the patients from TCGA LUAD (all *P* < 0.05, Figure 4(a)).

Further, we compared the immune cell subsets between the YAP1_High and YAP1_Low groups of EGFR-mutant patients in TCGA LUAD. The YAP1_High group showed a significant decrease in the proportion of immunoactive cells including NK cells, CD8^+^ T cells, and helper T cells compared with the YAP1_Low group (all *P* < 0.05, Figure 4(b)). YAP1_High tumors were rich in immunosuppressive cells infiltration (Figure 4(c)).

Additionally, we explored other factors that may affect this property. Previous studies have elucidated that TMB and mutation for quantification of tumor antigens are closely associated with immunotherapeutic efficacy [22, 23]. Therefore, we assessed TMB and mutations from the mutect2-processed TCGA LUAD between YAP1_High and YAP1_Low subtypes in EGFR-mutant patients. No significant difference was observed between the two subtypes in the number of mutant genes (*P*  >  0.05, Figure 4(d)) or TMB (*P*  >  0.05, Figure 4(e)). Accumulating evidence showed that PD-L1 is a predictive biomarker of response to ICIs treatment in NSCLC patients [24, 25]. Therefore, we analyzed the correlation between YAP1 and PD-L1 (CD274). As predicted, YAP1 was negatively correlated with PD-L1 in EGFR-mutant TCGA LUAD (Figure 4(f)) and GSE31210 (Figure 4(g)). Together, YAP1 was identified as a biomarker for immunosuppressive microenvironment in EGFR-mutant NSCLC.

### 3.5. Immune Landscape and YAP1 Expression in EGFR-Mutant NSCLC Patients

**Figure 5:**
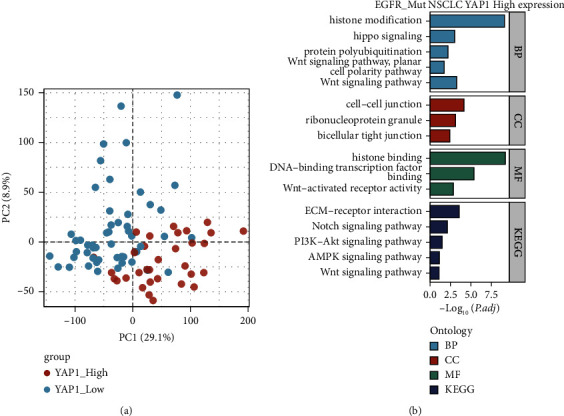
The immune landscape of EGFR-mutant NSCLC. (a) PC analysis in EGFR-mutant NSCLC patients revealed an obvious clustering between the YAP1_High and YAP1_Low groups. (b) GO and KEGG enrichment analysis of EGFR-mutant NSCLC in the YAP1_High group.

Based on the above results, we used PC analysis to test the immune landscape. The plots revealed an obvious clustering between the YAP1_High and YAP1_Low groups (Figure 5(a)). Next, the enrichment analysis of GO and KEGG between the two groups was performed. In the YAP1_High group, GO analysis showed the enrichment of histone modification, hippo signaling and Wnt signaling in biological process (BP), cell-cell junction and bicellular tight junction in cellular component (CC), histone binding, DNA-binding transcription factor binding, and Wnt-activated receptor activity in molecular function (MF). KEGG analysis showed the enrichment of ECM-receptor interaction, Notch signaling pathway, PI3K-Akt signaling pathway, and Wnt signaling pathway (Figure 5(b)).

### 3.6. Survival Analysis of ICIs Treatment Associated with YAP1 Expression in EGFR-Mutant NSCLC

**Figure 6:**
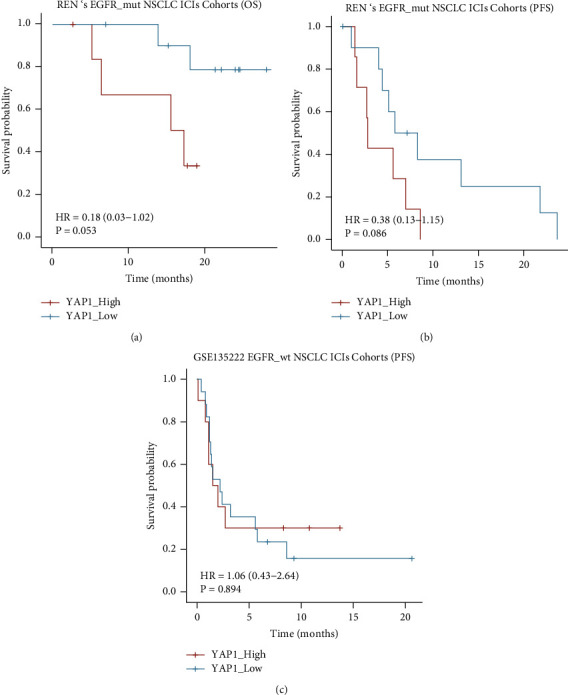
Poor survival after ICIs treatment from the YAP1_High group with EGFR-mutant NSCLC. (a) The YAP1_High group had a trend of shorter OS (15.6 months vs NR, *P*=0.053) and (b) shorter PFS (5.6 months vs 6.7 months, *P*=0.086) from Ren's research (NCT03513666). (c) The YAP1_High group had no different PFS with the YAP1_Low group in 27 EGFR-WT NSCLC patients receiving ICIs treatment from GSE13522 (1.8 months vs 2.4 months, *P*=0.894).

We also analyzed the role of YAP1 as a biomarker for predicting response to ICIs treatment in EGFR-mutant NSCLC. There were 18 EGFR-mutant NSCLC patients receiving ICIs treatment from Ren's research (NCT03513666) [26]. The YAP1_High group had a trend of shorter OS (15.6 months vs NR, *P*=0.053, Figure 6(a)) and shorter PFS (5.6 months vs 6.7 months, *P*=0.086, Figure 6(b)). There were 27 EGFR-WT NSCLC patients receiving ICIs treatment from GSE13522, and the YAP1_High group had no different PFS with the YAP1_Low group (1.8 months vs 2.4 months, *P*=0.894, Figure 6(c)). Taken together, YAP1 is a strong negative biomarker for predicting efficacy of ICIs treatment in EGFR-mutant NSCLC.

## 4. Discussion

As is known, the majority of patients experience insensitive response to ICIs monotherapy. Priority should be given to explorations of predictive biomarkers to determine which subpopulation of lung cancer patients will respond to ICIs. PD-L1 is regarded as the routine biomarker to predict the efficacy of ICIs treatment. NSCLC patients with high PD-L1 expression undergo durable response and achieve long PFS flowing ICIs treatment [27]. Also, TMB serves as a candidate biomarker for predicting the efficacy of ICIs monotherapy in various solid tumors [14, 28, 29]. However, not all NSCLC patients, especially the EGFR-mutant population, are unsuitable for ICIs treatment.

EGFR-TKIs are the preferred choice for EGFR-mutant NSCLC [30, 31], but whether these patients could benefit from ICIs treatment remains unknown. Recently, clinicians and researchers focused on the precision and individualized ICIs treatment for these patients [32–34]. Due to the complexity of the tumor immune microenvironment, it is likely insufficient to predict the response to ICIs treatment in NSCLC based on PD-L1 or TMB alone. In fact, the mechanism underlying immunotherapeutic effects on EGFR-mutant NSCLC is unclear. Dong et al. reported that EGFR-mutant patients were characterized by the immunosuppressive status, leading to decreased PD-L1^+^/CD8^+^ TILs compared with EGFR-WT NSCLC patients [35]. Tu et al. found that T-cell activity may play a role in response to ICIs treatment and T-cell infiltration was absent in these patients [36]. In addition, increased immunosuppressive cell types and decreased expression of immune checkpoint proteins generated an immune-silent environment in EGFR-mutant NSCLC [37]. Together, immune environment in EGFR-mutant tumors is possibly a key factor to determine which population could benefit from ICIs treatment.

One study reported that ICIs as monotherapy or in combination with chemotherapy can be used as the first-line treatment of advanced EGFR-mutant NSCLC [38]. Meanwhile, numerous case reports have shown that ICIs treatment appears to be beneficial for EGFR-mutant NSCLC patients after EGFR-TKIs resistance [39, 40]. Due to the heterogeneity of tumors, genomic changes are insufficient to serve as biomarkers for ICIs treatment. To date, no biomarker is available to select the patients suitable for ICIs treatment from EGFR-mutant population. Thus, it is essential to identify novel biomarkers to accurately select the lung cancer patients who will benefit from ICIs treatment for EGFR-mutant NSCLC.

In the current work, we performed bioinformatic analysis to explore potential immunity-related markers which could have an effect on the immunotherapeutic efficacy in EGFR-mutant population. YAP1 was found to be a possible novel negative biomarker to select the patients who could respond to ICIs treatment. Furthermore, we divided these patients into two immune subtypes. In our results, compared with YAP1_Low patients, YAP1_High subpopulation had a poorer prognosis in EGFR-mutant NSCLC patients from TCGA LUAD. However, we found that the OS of the YAP1_High group was numerically shorter than that of the YAP1_Low group in GSE11969 and GSE72094. This is mainly attributed to the small sample size. If the sample size is enlarged, the OS difference will probably become more obvious. Tumor microenvironment (TME) is an essential factor that may affect immunotherapeutic efficacy. YAP1_high tumor with EGFR mutation had a lower activated immune cell infiltration such as NK cells, CD8^+^ T cells, and helper T cells than YAP1_low tumor. Therefore, the insensitivity to ICIs treatment of the EGFR-mutant NSCLC patients with a high YAP1 expression is possibly due to a low infiltration of immune cells in the TME. Importantly, we verified that the OS and PFS after ICIs treatment were shorter in the YAP1_High group than in the YAP1_Low group in EGFR-mutant NSCLC patients from Ren's research [26] (NCT03513666), but not in EGFR-WT NSCLC patients from GSE13522. To our best knowledge, this is the first study to report that YAP1 is associated with immune subtypes in EGFR-mutant NSCLC and is a novel negative biomarker of ICIs treatment.

Additionally, our previous work has demonstrated that YAP1 plays an important role in self-renewal of cancer stem cells and its activity is negatively correlated with patient outcome [21]. A recent study found that interferon-*γ* induced tumor resistance to anti-PD-1 immunotherapy by promoting YAP1 phase separation [41]. All these reports strengthened the credibility of the results of our research.

Interestingly, from the TCGA database containing 6 types of tumors, the pooled analysis showed that YAP1 expression was significantly negatively correlated with OS after ICIs treatment. In the future, further studies will be needed to elucidate the mechanism.

Despite a small sample size due to the limitation that EGFR-mutant patients are not recommended to receive ICIs treatment, our findings are still meaningful and interesting and might provide some hints for further studies and potential directions for development of ICIs treatment for EGFR-mutant NSCLC.

## 5. Conclusions

Taken together, YAP1 mediates immunosuppressive microenvironment and poor prognosis in EGFR-mutant NSCLC population. YAP1 serves as a novel negative biomarker for ICIs treatment in NSCLC. Further studies on YAP1 might be promising and significant to individualized and precision ICIs treatment for NSCLC.

## Data Availability

The datasets used in the present study are obtained from the corresponding author upon reasonable request. Our data were obtained from Gene Expression Omnibus (GEO), TCGA LUAD and a phase-II, multicenter, open-label trial from Ren's research (NCT03513666).

## Abbreviations

YAP1: Yes1-associated transcriptional regulator
NSCLC: Non-small-cell lung cancer
EGFR: Epidermal growth factor receptor
TKI: Tyrosine kinase inhibitor
LUAD: Lung adenocarcinoma
ICI: Immune checkpoint inhibitor
PD-1: Programmed death protein 1
PD-L1: Programmed death-ligand 1
PFS: Progression-free survival
OS: Overall survival
TMB: Tumor mutation burden
TME: Tumor microenvironment
MSI: Microsatellite instability
WT: Wild type
CTLA-4: Cytotoxic T-lymphocyte antigen 4
TP53: Tumor protein *p*53
RHEB: mTORC1 binding
KDM5C: Lysine demethylase 5C
DNMT3A: DNA methyltransferase 3 alpha
DNMT3B: DNA methyltransferase 3 beta
EPHA5: EPH receptor A5
FAT1: FAT atypical cadherin 1
IGF2: Insulin-like growth factor 2
TBX3: T-box transcription factor 3
EPHA3: EPH receptor A3
TIIC: Tumor-infiltrating immune cell.

## Supplementary Materials

Supplementary Materials All the detailed dataset information including LUAD dataset, GSE11969, GSE72094, GSE31210, GSE13522, immune infiltration, mutation enrichment, GO-KEGG, and the phase-II trial from Prof. Ren's lab (NCT03513666) is shown in Supplementary Table 1. The PFS, OS, and the expression of YAP1 are included. The immune infiltration of B cells, CD8^+^/ CD4^+^ T cells, Tregs, NK cells, macrophages M1/M2, and dendritic cells and the term of GO-KEGG as well as mutation enrichment are also provided (Supplementary Table 1). All our analyses were based on the above information.
