# Supplementary material for: YAP1 as a Novel Negative Biomarker of Immune Checkpoint Inhibitors for EGFR-Mutant Non-Small-Cell Lung Cancer
Source: Can Respir J. 2023 Jun 21;2023:4689004. doi: 10.1155/2023/4689004 (PMC10307059; doi:10.1155/2023/4689004)
Supplement: Supplementary Materials — All the detailed dataset information including LUAD dataset, GSE11969, GSE72094, GSE31210, GSE13522, immune infiltration, mutation enrichment, GO-KEGG, and the phase-II trial from Prof. Ren's lab (NCT03513666) is shown in Supplementary Table 1. The PFS, OS, and the expression of YAP1 are included. The immune infiltration of B cells, CD8+/ CD4+ T cells, Tregs, NK cells, macrophages M1/M2, and dendritic cells and the term of GO-KEGG as well as mutation enrichment are also provided (Supplementary Table 1). All our analyses were based on the above information. [file 4689004.f1.pdf]

TCGA_LUAD

Sample_id	OS_event	OS_time	PFS_event	PFS_time	YAP1_expression	EGFR
TCGA-55-8620-01	1	12.5	1	12.5	1.242056619	Wt
TCGA-55-5899-01	0	31	0	31	2.163269954	Wt
TCGA-44-7667-01	0	36.566666667	0	36.566666667	2.185623692	Wt
TCGA-55-6968-01	1	43.1	1	43.1	2.234752035	Mut
TCGA-86-8358-01	0	21.766666667	0	21.766666667	2.265098089	Wt
TCGA-44-5644-01	0	28.766666667	0	28.766666667	2.343362284	Wt
TCGA-4B-A93V-01	1	10	1	8.366666667	2.436434932	Wt
TCGA-49-AAR2-01	0	74.133333333	0	74.133333333	2.469280747	Wt
TCGA-44-7669-01	1	19.133333333	1	13	2.729972912	Wt
TCGA-49-4514-01	0	56.666666667	0	56.666666667	2.797529837	Wt
TCGA-49-AARE-01	1	40.966666667	1	12.933333333	2.8007718	Wt
TCGA-64-1678-01	0	39.633333333	0	39.633333333	2.865278249	Wt
TCGA-91-8499-01	0	1.2	0	1.2	2.902560114	Wt
TCGA-55-A490-01	1	3.3	0	3.3	2.955003271	Wt
TCGA-86-A4D0-01	1	3.866666667	0	3.866666667	2.984797747	Wt
TCGA-MP-A4TE-01	1	29.866666667	1	7.533333333	3.020724623	Wt
TCGA-55-8094-01	0	18.033333333	0	18.033333333	3.067544397	Wt
TCGA-64-5774-01	0	89.2	1	8.2	3.150479665	Wt
TCGA-55-7728-01	0	23.466666667	0	23.466666667	3.218339516	Wt
TCGA-35-4122-01	0	7.5	0	7.5	3.225889528	Wt
TCGA-55-7995-01	0	29.633333333	1	15.6	3.24100651	Wt
TCGA-49-4494-01	1	36.033333333	1	36.033333333	3.242563895	Mut
TCGA-64-5778-01	0	43.5	1	32.066666667	3.25852321	Wt
TCGA-95-A4VP-01	0	20.166666667	1	7.2	3.277806451	Wt
TCGA-49-AARQ-01	0	224.4	0	224.4	3.306556647	Wt
TCGA-49-AAR0-01	0	158.833333333	0	158.833333333	3.317435636	Wt
TCGA-80-5608-01	0	94.4	0	94.4	3.354498836	Wt
TCGA-55-8615-01	0	14.866666667	1	7.466666667	3.365117944	Wt
TCGA-49-6761-01	0	11.8	0	11.8	3.373186349	Wt
TCGA-55-8203-01	0	18.233333333	0	18.233333333	3.386906159	Wt
TCGA-35-5375-01	0	8.8	0	8.8	3.413714387	Wt
TCGA-86-8054-01	0	38.266666667	0	38.266666667	3.429290921	Wt
TCGA-NJ-A55R-01	0	20.1	0	20.1	3.461651682	Wt
TCGA-MP-A4TD-01	1	10.233333333	1	7.533333333	3.475931103	Wt
TCGA-55-8508-01	0	20.566666667	0	20.566666667	3.502279559	Wt
TCGA-MN-A4N1-01	0	27.566666667	0	27.566666667	3.504126557	Wt
TCGA-50-6597-01	1	42.266666667	0	42.266666667	3.521173508	Wt
TCGA-49-AAQV-01	1	22.566666667	1	16.966666667	3.523326976	Wt
TCGA-05-5420-01	0	15.233333333	1	8.166666667	3.532425963	Wt
TCGA-91-6835-01	0	2.633333333	0	2.633333333	3.536779202	Mut
TCGA-86-7953-01	0	33.233333333	0	33.233333333	3.547303151	Wt
TCGA-44-5643-01	0	33.766666667	0	33.766666667	3.562328149	Wt
TCGA-69-8255-01	0	4.3	0	4.3	3.56456479	Wt
TCGA-95-A4VN-01	0	18.433333333	0	18.433333333	3.592350247	Wt
TCGA-73-7499-01	1	51.033333333	1	48.233333333	3.600679924	Wt
TCGA-38-4632-01	1	45.233333333	1	22.666666667	3.605061827	Wt
TCGA-L9-A444-01	0	10.233333333	0	10.233333333	3.607474782	Wt
TCGA-44-7660-01	0	19.733333333	1	8.433333333	3.611131105	Wt
TCGA-44-6778-01	0	62.133333333	0	62.133333333	3.635479124	Wt
TCGA-49-4506-01	1	33.3	1	23.066666667	3.646803103	Wt
TCGA-55-8208-01	0	22.466666667	1	17.033333333	3.651161019	Wt
TCGA-50-5066-01	0	48.066666667	1	15.9	3.65960594	Mut
TCGA-86-8585-01	0	11.766666667	0	11.766666667	3.66616608	Wt
TCGA-50-5931-01	1	14.466666667	1	14.166666667	3.669346226	Wt
TCGA-99-8032-01	0	1.466666667	0	1.466666667	3.683058193	Wt
TCGA-55-A4DF-01	1	20.466666667	1	17.166666667	3.683338595	Wt
TCGA-44-7672-01	0	23.966666667	0	23.966666667	3.687495155	Wt
TCGA-78-7166-01	1	8.6	0	8.6	3.697877531	Wt

TCGA-62-A471-01	0	41.533333333	0	41.533333333	3.70560262	Wt
TCGA-91-A4BD-01	0	20.1	0	20.1	3.711724329	Wt
TCGA-86-8673-01	0	28.733333333	1	21.2	3.713567926	Wt
TCGA-62-A46P-01	1	19.8	1	8.9	3.715165492	Wt
TCGA-95-7043-01	1	16.766666667	1	16.766666667	3.716080815	Wt
TCGA-MN-A4N5-01	0	2.8	0	2.8	3.728386732	Wt
TCGA-62-A46S-01	1	55.1	1	17.566666667	3.73466567	Wt
TCGA-99-8028-01	0	37.266666667	0	37.266666667	3.770762492	Wt
TCGA-55-6987-01	0	71.233333333	0	71.233333333	3.783131003	Wt
TCGA-05-4403-01	0	19.266666667	0	19.266666667	3.785780212	Wt
TCGA-55-8302-01	0	15.933333333	0	15.933333333	3.806368881	Wt
TCGA-95-8494-01	0	2.8	0	2.8	3.825114327	Wt
TCGA-44-4112-01	1	26.933333333	1	20.633333333	3.833320495	Wt
TCGA-50-5051-01	1	15.933333333	1	6.133333333	3.844354356	Wt
TCGA-69-8453-01	0	27.1	1	11.466666667	3.853966863	Wt
TCGA-55-A491-01	0	20.866666667	0	20.866666667	3.855062418	Wt
TCGA-49-AAR4-01	1	29.3	1	29.3	3.857162851	Wt
TCGA-44-8119-01	0	9.5	0	9.5	3.864360508	Wt
TCGA-05-4389-01	0	45.633333333	0	45.633333333	3.881941141	Wt
TCGA-50-5045-01	1	72.466666667	1	47.766666667	3.885585904	Wt
TCGA-55-A493-01	0	0.933333333	0	0.933333333	3.885998515	Wt
TCGA-64-5815-01	0	28.866666667	0	28.866666667	3.894796319	Wt
TCGA-38-4628-01	1	49.733333333	1	36.1	3.896731914	Mut
TCGA-55-7281-01	0	29.066666667	1	11.3	3.905666383	Wt
TCGA-95-8039-01	0	27.666666667	1	7.6	3.91582095	Wt
TCGA-49-4507-01	1	8.933333333	1	5.266666667	3.918963147	Wt
TCGA-49-4512-01	1	30.166666667	1	30.166666667	3.925777661	Wt
TCGA-50-5044-01	1	20.8	1	16.1	3.926157771	Wt
TCGA-75-5147-01	0	44.433333333	0	44.433333333	3.932953156	Wt
TCGA-86-7701-01	0	31.566666667	1	14.133333333	3.943489118	Wt
TCGA-50-5068-01	1	49.966666667	1	22.866666667	3.944855008	Wt
TCGA-05-4433-01	0	24.333333333	0	24.333333333	3.94970064	Wt
TCGA-93-8067-01	0	6.2	0	6.2	3.952942459	Wt
TCGA-78-7220-01	1	26.9	1	17.7	3.959652257	Wt
TCGA-44-A4SS-01	0	13.833333333	0	13.833333333	3.962107834	Wt
TCGA-55-1594-01	0	39.266666667	0	39.266666667	3.962233598	Wt
TCGA-75-6211-01	1		1	0	3.963610315	Wt
TCGA-55-7283-01	0	20.3	0	20.3	3.964640168	Wt
TCGA-05-5429-01	1	9.166666667	0	9.166666667	3.964833452	Wt
TCGA-49-4487-01	1	28.5	1	23.233333333	3.966529015	Wt
TCGA-97-8176-01	1	15.6	1	1.3	3.975022743	Wt
TCGA-50-5055-01	1	61	1	25.7	3.979786467	Wt
TCGA-55-6983-01	0	94.1	0	94.1	4.00224814	Wt
TCGA-MP-A4TJ-01	1	11.3	0	11.3	4.006325996	Wt
TCGA-44-3917-01	0	39.433333333	0	39.433333333	4.011650435	Wt
TCGA-44-A479-01	0	16.2	1	14.566666667	4.020039765	Wt
TCGA-NJ-A55O-01	0	0.433333333	0	0.433333333	4.022233357	Wt
TCGA-05-4390-01	0	37.533333333	1	13.166666667	4.022272359	Wt
TCGA-L9-A8F4-01	0	15.866666667	0	15.866666667	4.026329284	Wt
TCGA-78-7159-01	0	65.8	0	65.8	4.028110504	Wt
TCGA-55-A494-01	0	16.033333333	0	16.033333333	4.031207558	Wt
TCGA-38-4625-01	0	99.1	0	99.1	4.036834167	Wt
TCGA-05-4432-01	0	25.366666667	0	25.366666667	4.050391714	Wt
TCGA-44-3918-01	0	34.533333333	1	17.033333333	4.052062763	Wt
TCGA-86-8074-01	0	0.8	0	0.8	4.053881721	Mut
TCGA-05-5428-01	0	22.333333333	0	22.333333333	4.066663199	Wt
TCGA-05-4417-01	0	15.166666667	0	15.166666667	4.067697408	Wt
TCGA-99-8025-01	0	35.333333333	0	35.333333333	4.074909497	Wt
TCGA-55-6971-01	0	46.666666667	0	46.666666667	4.076198612	Wt
TCGA-86-8671-01	0	27.966666667	0	27.966666667	4.077046754	Wt
TCGA-69-7973-01	0	7.666666667	0	7.666666667	4.080756184	Wt
TCGA-64-5781-01	0	51.966666667	1	3.2	4.081093038	Wt

TCGA-49-4488-01	1	28.966666667	1	21.133333333	4.086719289	Wt
TCGA-NJ-A4YF-01	0	72.033333333	0	72.033333333	4.088268064	Wt
TCGA-73-4659-01	1	23.7	1	1.166666667	4.088699083	Wt
TCGA-L4-A4E6-01	0	14.5	0	14.5	4.099101812	Wt
TCGA-NJ-A4YQ-01	0	47.733333333	0	47.733333333	4.109150796	Wt
TCGA-55-8510-01	0	17.966666667	0	17.966666667	4.109481801	Wt
TCGA-38-A44F-01	0	4.433333333	0	4.433333333	4.113242578	Wt
TCGA-95-7944-01	0	12.566666667	0	12.566666667	4.121240215	Wt
TCGA-05-4418-01	1	9.133333333	0	9.133333333	4.13335106	Wt
TCGA-50-5049-01	0	103.133333333	1	52.266666667	4.139153517	Wt
TCGA-L9-A743-01	0	22.133333333	0	22.133333333	4.141958459	Wt
TCGA-38-4629-01	1	28.8	1	12.633333333	4.146139332	Mut
TCGA-62-8395-01	0	40.533333333	1	13.166666667	4.14915488	Wt
TCGA-55-7903-01	0	18.9	0	18.9	4.149570184	Wt
TCGA-86-A456-01	0	29.866666667	0	29.866666667	4.154344717	Wt
TCGA-62-A470-01	1	39.8	1	17.966666667	4.158350463	Wt
TCGA-49-4510-01	1	29.866666667	1	16.733333333	4.158361808	Wt
TCGA-35-4123-01	0	6.066666667	0	6.066666667	4.164957474	Wt
TCGA-L9-A443-01	1	6.433333333	0	6.433333333	4.171751514	Wt
TCGA-50-8460-01	0	27.633333333	0	27.633333333	4.187923607	Wt
TCGA-91-A4BC-01	0	1.466666667	0	1.466666667	4.188487294	Wt
TCGA-78-7540-01	1	39.9	0	39.9	4.196743869	Wt
TCGA-86-8674-01	0	26.866666667	1	11.133333333	4.201531626	Wt
TCGA-75-7031-01	0		0	0	4.201850555	Wt
TCGA-MP-A4SW-01	1	59.266666667	0	59.266666667	4.201911165	Mut
TCGA-05-4398-01	0	47.7	0	47.7	4.202173761	Wt
TCGA-44-A47G-01	0	11.7	0	11.7	4.202187574	Wt
TCGA-86-A4JF-01	1	24.566666667	1	14.533333333	4.204739584	Wt
TCGA-78-8648-01	1	40.3	1	24	4.214786719	Wt
TCGA-38-7271-01	1	26.666666667	1	10.133333333	4.216510018	Wt
TCGA-75-5122-01	1		1	0	4.21784173	Wt
TCGA-91-6831-01	0	10.333333333	0	10.333333333	4.218571122	Wt
TCGA-55-8301-01	0	17.8	1	7.933333333	4.220842705	Wt
TCGA-55-7284-01	1	8.1	1	7.7	4.224696488	Wt
TCGA-99-AA5R-01	0	21.933333333	0	21.933333333	4.227698281	Wt
TCGA-55-6979-01	1	7.9	1	6.5	4.235869426	Wt
TCGA-49-4505-01	1	14.266666667	1	13.9	4.24084034	Wt
TCGA-97-8547-01	0	21.9	0	21.9	4.243470197	Mut
TCGA-69-7760-01	0	6.733333333	0	6.733333333	4.25208609	Mut
TCGA-62-8397-01	0	42.966666667	0	42.966666667	4.252646906	Mut
TCGA-73-4662-01	0	83.833333333	1	0.7	4.254866318	Wt
TCGA-97-8552-01	0	20.866666667	0	20.866666667	4.255491552	Mut
TCGA-44-7670-01	0	29.4	0	29.4	4.255873917	Wt
TCGA-05-4420-01	0	30.4	0	30.4	4.256622058	Wt
TCGA-55-8621-01	0	17.166666667	0	17.166666667	4.256933256	Wt
TCGA-78-7537-01	1	54.066666667	0	54.066666667	4.257314169	Wt
TCGA-MP-A4T7-01	1	5.566666667	0	5.566666667	4.266936915	Wt
TCGA-MP-A4T8-01	1	5.366666667	0	5.366666667	4.267977405	Wt
TCGA-55-A48Z-01	0	21.7	1	17.866666667	4.272420995	Mut
TCGA-80-5611-01	0	86.5	0	86.5	4.275891341	Wt
TCGA-55-8507-01	0	13.933333333	0	13.933333333	4.276587248	Wt
TCGA-49-6742-01	1	16.266666667	1	7.133333333	4.276644576	Wt
TCGA-05-4427-01	0	26.366666667	0	26.366666667	4.280440189	Wt
TCGA-50-8459-01	0	37.3	1	14.4	4.28484243	Wt
TCGA-97-8177-01	0	16.633333333	0	16.633333333	4.28972257	Mut
TCGA-49-4486-01	1	77.266666667	1	68.166666667	4.293589882	Wt
TCGA-64-1681-01	1	38.9	1	14.633333333	4.295443083	Mut
TCGA-97-7941-01	0	16.133333333	0	16.133333333	4.295844214	Wt
TCGA-55-8205-01	0	19.966666667	1	16.5	4.295922737	Wt
TCGA-55-A492-01	0	19.866666667	0	19.866666667	4.297438232	Wt
TCGA-05-4430-01	0	25.366666667	0	25.366666667	4.298498337	Wt
TCGA-55-6984-01	1	25.333333333	1	24.133333333	4.299743915	Wt
TCGA-97-A4LX-01	0	20.466666667	0	20.466666667	4.300146998	Wt

TCGA-97-A4M2-01	0	20.8	0	20.8	4.306458954	Wt
TCGA-44-3398-01	0	38.766666667	0	38.766666667	4.308325561	Wt
TCGA-97-8179-01	0	14.5	0	14.5	4.309412514	Wt
TCGA-91-6848-01	0	7.466666667	0	7.466666667	4.311188235	Wt
TCGA-44-2661-01	0	38.633333333	0	38.633333333	4.318609969	Mut
TCGA-67-3773-01	0	14.233333333	0	14.233333333	4.320801476	Wt
TCGA-86-8359-01	1	14.8	0	14.8	4.321870069	Wt
TCGA-05-4397-01	1	24.366666667	0	24.366666667	4.322755471	Wt
TCGA-55-8505-01	0	14.666666667	0	14.666666667	4.324625042	Wt
TCGA-93-A4JO-01	1	1.1	0	1.1	4.325172321	Wt
TCGA-49-AAR3-01	0	63.1	1	63.1	4.329335055	Wt
TCGA-49-AAR9-01	1	8.666666667	1	8.666666667	4.329717558	Wt
TCGA-05-5715-01	0	2.066666667	0	2.066666667	4.332687234	Wt
TCGA-55-7911-01	0	17.9	1	17.166666667	4.337164748	Wt
TCGA-O1-A52J-01	1	59.933333333	1	29.933333333	4.338077198	Wt
TCGA-78-7148-01	1	20.866666667	1	6.066666667	4.345033659	Wt
TCGA-L9-A7SV-01	0	18.833333333	0	18.833333333	4.347254853	Wt
TCGA-86-A4P7-01	0	13.833333333	0	13.833333333	4.350333101	Mut
TCGA-75-5126-01	0		0	0	4.355015404	Wt
TCGA-MP-A4TI-01	1	14.3	1	2.8	4.356377305	Wt
TCGA-MP-A4TK-01	1	19.4	1	13.233333333	4.356684748	Wt
TCGA-86-8672-01	1	0.633333333	0	0.633333333	4.362125665	Wt
TCGA-55-8085-01	0	30.133333333	0	30.133333333	4.362276269	Wt
TCGA-55-8513-01	0	26.366666667	1	10.566666667	4.36537725	Wt
TCGA-49-4490-01	1	12.833333333	1	12.833333333	4.366357724	Mut
TCGA-05-5423-01	0	5.033333333	0	5.033333333	4.368106028	Wt
TCGA-93-7347-01	0	22.766666667	0	22.766666667	4.371272655	Wt
TCGA-50-5941-01	0	49.133333333	0	49.133333333	4.372907531	Wt
TCGA-78-7145-01	1	27.533333333	1	14.066666667	4.373227676	Wt
TCGA-93-A4JQ-01	0	17.533333333	0	17.533333333	4.374868037	Wt
TCGA-44-6146-01	0	24.266666667	1	21.033333333	4.380606637	Wt
TCGA-73-4670-01	0	4.366666667	0	4.366666667	4.381214187	Wt
TCGA-78-7542-01	1	10.7	0	10.7	4.383673163	Wt
TCGA-78-7536-01	1	8.133333333	1	7.7	4.384773252	Wt
TCGA-91-8497-01	1	14.466666667	0	14.466666667	4.385920683	Wt
TCGA-44-3918-01	0	34.533333333	1	17.033333333	4.386993219	Wt
TCGA-55-7907-01	1	11.433333333	1	9.8	4.389759817	Wt
TCGA-MP-A4T4-01	1	87.233333333	0	87.233333333	4.392897508	Wt
TCGA-55-6972-01	1	54.4	0	54.4	4.394455985	Wt
TCGA-NJ-A4YP-01	0	1.666666667	0	1.666666667	4.395358885	Wt
TCGA-55-8514-01	0	17.333333333	0	17.333333333	4.406459429	Wt
TCGA-49-AARO-01	0	125.3	1	38.133333333	4.408540102	Wt
TCGA-53-7813-01	0	14.133333333	0	14.133333333	4.40994241	Wt
TCGA-MP-A4SY-01	1	50.033333333	1	16.3	4.411284994	Wt
TCGA-55-8619-01	0	13.866666667	0	13.866666667	4.421488442	Wt
TCGA-62-8398-01	1	14.8	0	14.8	4.424364053	Wt
TCGA-50-6593-01	1	11.2	1	8.866666667	4.428026357	Wt
TCGA-55-6980-01	0	70.3	0	70.3	4.429755914	Mut
TCGA-97-8175-01	0	18.366666667	1	9.8	4.433003752	Wt
TCGA-69-7980-01	0	13.7	0	13.7	4.442417526	Wt
TCGA-05-4424-01	0	30.433333333	1	5.1	4.447485556	Wt
TCGA-62-A472-01	0	30.333333333	1	9.666666667	4.448269175	Wt
TCGA-64-1680-01	0	37.533333333	0	37.533333333	4.45198318	Wt
TCGA-49-AARR-01	0	166.4	1	36.633333333	4.453867958	Wt
TCGA-78-8655-01	0	78.666666667	0	78.666666667	4.456406688	Wt
TCGA-78-7535-01	1	31.633333333	1	26.966666667	4.459549038	Wt
TCGA-78-7149-01	0	131.333333333	0	131.333333333	4.468697392	Wt
TCGA-86-A4P8-01	0	26.833333333	0	26.833333333	4.473053577	Wt
TCGA-NJ-A4YG-01	0	75.366666667	0	75.366666667	4.473883613	Wt
TCGA-64-5779-01	0	28.8	1	26.5	4.474133484	Wt
TCGA-MN-A4N4-01	0	39.166666667	0	39.166666667	4.475099359	Wt
TCGA-62-8402-01	1	49.933333333	1	25.733333333	4.480039258	Mut
TCGA-05-4250-01	1	4.033333333	0	4.033333333	4.489615325	Wt

TCGA-38-4626-01	0	122.466666667	1	83.933333333	4.496782326	Wt
TCGA-44-6145-01	0	19.833333333	0	19.833333333	4.499832782	Wt
TCGA-64-1679-01	0	82.933333333	0	82.933333333	4.501660303	Wt
TCGA-97-A4M7-01	0	20.966666667	0	20.966666667	4.502675929	Mut
TCGA-97-A4M5-01	0	21.133333333	0	21.133333333	4.502714762	Wt
TCGA-78-7161-01	1	9.7	1	5.4	4.504760598	Wt
TCGA-44-2657-01	0	45.033333333	0	45.033333333	4.518771405	Wt
TCGA-69-7979-01	0	13.6	0	13.6	4.520917495	Wt
TCGA-44-3396-01	0	37.666666667	0	37.666666667	4.521471248	Wt
TCGA-55-7576-01	0	22.333333333	0	22.333333333	4.522339386	Wt
TCGA-55-8512-01	1	20.233333333	1	20.233333333	4.522570151	Wt
TCGA-91-8496-01	0	16.833333333	0	16.833333333	4.522682592	Wt
TCGA-55-6985-01	0	41.1	0	41.1	4.534846528	Wt
TCGA-97-A4M3-01	0	18	1	1.8	4.535151534	Wt
TCGA-73-4658-01	1	53.333333333	0	53.333333333	4.543063754	Wt
TCGA-55-6642-01	0	81.633333333	0	81.633333333	4.543221266	Wt
TCGA-J2-A4AD-01	1	18.333333333	1	17.466666667	4.543371088	Wt
TCGA-53-7626-01	1	30.966666667	1	28.833333333	4.546074255	Wt
TCGA-73-4675-01	1	30.733333333	1	12.033333333	4.547653851	Wt
TCGA-78-8660-01	1	10.7	1	8.066666667	4.551714504	Wt
TCGA-35-3615-01	0	0.466666667	0	0.466666667	4.554814983	Wt
TCGA-69-7978-01	0	4.466666667	0	4.466666667	4.557137036	Wt
TCGA-50-7109-01	1	10.266666667	1	0.5	4.558200149	Wt
TCGA-86-8669-01	0	31.266666667	1	28.3	4.558480082	Wt
TCGA-95-7039-01	0	42.4	1	41.933333333	4.558983624	Mut
TCGA-86-7954-01	0	20.166666667	0	20.166666667	4.560376451	Wt
TCGA-64-1676-01	0	57.6	0	57.6	4.560621415	Wt
TCGA-L4-A4E5-01	0	19.266666667	0	19.266666667	4.562584578	Wt
TCGA-97-7547-01	0	65.5	1	25.833333333	4.568252598	Wt
TCGA-91-6828-01	0	10.766666667	0	10.766666667	4.568556943	Wt
TCGA-64-1677-01	1	20.933333333	1	12	4.568800879	Wt
TCGA-55-7815-01	0	25.766666667	1	15.533333333	4.573403984	Wt
TCGA-50-5930-01	1	9.4	1	5.9	4.581915586	Wt
TCGA-67-3770-01	0	20.333333333	0	20.333333333	4.584510324	Mut
TCGA-38-4631-01	1	11.8	1	11.8	4.58583549	Wt
TCGA-86-8076-01	0	33.1	0	33.1	4.586874109	Wt
TCGA-75-7025-01	0	110.166666667	1	49.366666667	4.59523693	Mut
TCGA-S2-AA1A-01	0	17.1	0	17.1	4.595415996	Wt
TCGA-71-8520-01	1	7	1	5.966666667	4.999837791	Mut
TCGA-50-6590-01	1	42.933333333	0	42.933333333	4.611261683	Wt
TCGA-86-7711-01	1	34.866666667	1	34.866666667	4.614205237	Wt
TCGA-55-8097-01	0	15.866666667	0	15.866666667	4.614956922	Wt
TCGA-86-6851-01	0	5.966666667	0	5.966666667	4.620912732	Wt
TCGA-55-6981-01	1	45.966666667	0	45.966666667	4.621691191	Mut
TCGA-MP-A4TH-01	0	24.7	0	24.7	4.621719388	Wt
TCGA-91-6836-01	0	13.9	0	13.9	4.628235535	Wt
TCGA-44-6777-01	1	32.9	0	32.9	4.632987227	Wt
TCGA-95-7947-01	0	15.9	0	15.9	4.634117562	Mut
TCGA-50-6594-01	1	12.333333333	1	9.5	4.635233414	Wt
TCGA-55-7727-01	0	3.966666667	0	3.966666667	4.635824308	Wt
TCGA-53-A4EZ-01	0	35.7	0	35.7	4.639016403	Wt
TCGA-78-8662-01	1	112.033333333	1	101.466666667	4.641027559	Wt
TCGA-69-7764-01	0	13.8	0	13.8	4.642272682	Wt
TCGA-69-7761-01	0	6.2	0	6.2	4.643194011	Wt
TCGA-49-AARN-01	1	37.833333333	0	37.833333333	4.644347367	Wt
TCGA-NJ-A7XG-01	0	20.566666667	0	20.566666667	4.645506424	Wt
TCGA-78-7160-01	1	23.233333333	0	23.233333333	4.646629909	Wt
TCGA-75-6214-01	1	37.166666667	1	13.966666667	4.648135034	Wt
TCGA-62-A46Y-01	1	13.8	1	10.466666667	4.648279174	Wt
TCGA-44-7659-01	0	23.033333333	0	23.033333333	4.648697745	Wt
TCGA-75-6205-01	1		1	0	4.64931815	Wt
TCGA-78-8640-01	0	235.4	0	235.4	4.649992856	Wt
TCGA-97-A4M1-01	0	20.033333333	0	20.033333333	4.650972813	Mut

TCGA-97-7553-01	0	62.333333333	0	62.333333333	4.654405222	Wt
TCGA-97-7552-01	0	64.4	1	26.6	4.658432681	Wt
TCGA-50-5072-01	1	8.333333333	1	7.1	4.662726554	Wt
TCGA-73-4676-01	1	9.366666667	1	9.366666667	4.664293423	Wt
TCGA-55-8090-01	1	19.933333333	1	18.266666667	4.665473625	Wt
TCGA-50-6592-01	1	25.9	1	25.9	4.669415309	Wt
TCGA-73-7498-01	0	39.633333333	0	39.633333333	4.671393921	Wt
TCGA-38-4627-01	1	38.233333333	0	38.233333333	4.676548937	Mut
TCGA-97-A4M6-01	0	18.933333333	0	18.933333333	4.680707593	Mut
TCGA-67-3772-01	0	19.1	0	19.1	4.681345009	Mut
TCGA-44-6146-01	0	24.266666667	1	21.033333333	4.683094836	Wt
TCGA-MP-A4T9-01	1	42.166666667	1	11.366666667	4.686497561	Mut
TCGA-62-8394-01	1	4.633333333	0	4.633333333	4.690003128	Mut
TCGA-93-A4JP-01	0	19.266666667	1	16.566666667	4.69428335	Mut
TCGA-05-4244-01	0	0	0	0	4.70036676	Wt
TCGA-86-7713-01	0	38.566666667	0	38.566666667	4.702142701	Wt
TCGA-55-7910-01	0	34.666666667	1	33.933333333	4.7060271	Wt
TCGA-75-7027-01	0	101.966666667	1	98.3	4.707055745	Wt
TCGA-78-7162-01	1	105.633333333	1	73.933333333	4.708340445	Wt
TCGA-L9-A5IP-01	1	1.933333333	1	1.633333333	4.708733304	Wt
TCGA-91-6847-01	0	28.066666667	1	25.733333333	4.709324042	Wt
TCGA-78-7153-01	0	121.166666667	0	121.166666667	4.711345752	Wt
TCGA-67-6216-01	0	4.7	0	4.7	4.715366332	Wt
TCGA-44-2655-01	0	44.133333333	1	33.633333333	4.72118216	Wt
TCGA-75-7030-01	0		0	0	4.722509457	Wt
TCGA-75-5146-01	0	78.933333333	1	59.1	4.727425149	Wt
TCGA-69-8254-01	0	13.633333333	0	13.633333333	4.728114622	Wt
TCGA-05-4382-01	0	20.233333333	1	11.133333333	4.731054018	Mut
TCGA-44-2665-01	0	43.366666667	0	43.366666667	4.738677013	Wt
TCGA-44-2656-01	0	47.633333333	1	18.933333333	4.738802656	Wt
TCGA-05-4434-01	1	15.233333333	0	15.233333333	4.73955148	Wt
TCGA-75-6212-01	1	50.533333333	1	44.666666667	4.741073224	Wt
TCGA-55-7227-01	1	31.733333333	1	8.5	4.744510306	Wt
TCGA-97-A4M0-01	0	21.733333333	0	21.733333333	4.747353061	Wt
TCGA-69-7763-01	0	23	0	23	4.748682415	Wt
TCGA-55-A48X-01	0	22.966666667	1	20.5	4.749640801	Wt
TCGA-78-7154-01	1	19.766666667	0	19.766666667	4.751003986	Wt
TCGA-55-6970-01	1	15.466666667	1	15.233333333	4.753674937	Wt
TCGA-93-A4JN-01	0	23.933333333	0	23.933333333	4.75704398	Wt
TCGA-44-2668-01	1	25.366666667	1	14.433333333	4.757991926	Wt
TCGA-55-1596-01	0	68.833333333	0	68.833333333	4.760558008	Wt
TCGA-05-4249-01	0	50.766666667	0	50.766666667	4.760932814	Wt
TCGA-44-3919-01	1	34.2	1	30.7	4.76464565	Wt
TCGA-NJ-A55A-01	0	0.5	0	0.5	4.769478023	Wt
TCGA-44-7671-01	0	29.633333333	1	29.433333333	4.769791423	Wt
TCGA-05-4395-01	1	0	0	0	4.772928139	Wt
TCGA-MP-A4T6-01	1	59.666666667	0	59.666666667	4.7760866	Wt
TCGA-55-7574-01	1	33.166666667	1	15.933333333	4.776460269	Wt
TCGA-49-6744-01	0	56.1	0	56.1	4.784540261	Wt
TCGA-91-7771-01	0	16.4	0	16.4	4.786324456	Wt
TCGA-55-A48Y-01	0	21	0	21	4.791273719	Wt
TCGA-55-6978-01	1	5.866666667	1	1.4	4.792528771	Wt
TCGA-55-8206-01	0	29.6	0	29.6	4.792793356	Mut
TCGA-67-6217-01	0	14.066666667	1	9.866666667	4.796705907	Mut
TCGA-55-7725-01	0	14.733333333	0	14.733333333	4.798975352	Wt
TCGA-55-8204-01	0	17.166666667	0	17.166666667	4.800083459	Wt
TCGA-44-7661-01	1	18.566666667	1	11.166666667	4.803957936	Wt
TCGA-55-7816-01	1	15.6	1	15.6	4.807868143	Wt
TCGA-67-3771-01	0	20.333333333	0	20.333333333	4.8099124	Mut
TCGA-49-4501-01	1	47.366666667	1	18.166666667	4.8120311	Mut
TCGA-69-8253-01	0	14.2	0	14.2	4.816055249	Wt
TCGA-55-7913-01	1	18.7	1	16	4.817528024	Wt
TCGA-05-4384-01	0	14.2	1	6.1	4.81870719	Wt

TCGA-97-8172-01	0	18.166666667	0	18.166666667	4.821709707	Wt
TCGA-55-8089-01	1	23.4	0	23.4	4.824775268	Wt
TCGA-69-7765-01	0	5.5	0	5.5	4.828708727	Mut
TCGA-78-7152-01	1	40.5	1	40.066666667	4.832302358	Wt
TCGA-55-8299-01	1	15.633333333	1	9.133333333	4.836331888	Wt
TCGA-55-6982-01	1	33.166666667	1	6.1	4.841350903	Wt
TCGA-86-8075-01	1	23.133333333	1	6.633333333	4.845065301	Mut
TCGA-67-6215-01	0	5.8	0	5.8	4.851195205	Wt
TCGA-MP-A4SV-01	1	87.333333333	0	87.333333333	4.851228346	Wt
TCGA-86-8056-01	0	4.633333333	0	4.633333333	4.854426707	Wt
TCGA-50-5946-01	0	53.9	1	7.366666667	4.854595991	Wt
TCGA-69-A59K-01	0	19.7	0	19.7	4.863408701	Wt
TCGA-86-8073-01	0	24.666666667	0	24.666666667	4.863872371	Wt
TCGA-05-4405-01	0	20.333333333	0	20.333333333	4.867914161	Wt
TCGA-44-2666-01	1	3.233333333	1	3.233333333	4.869504209	Wt
TCGA-86-8055-01	1	4.133333333	0	4.133333333	4.871081024	Mut
TCGA-44-2656-01	0	47.633333333	1	18.933333333	4.874339769	Wt
TCGA-55-7570-01	0	27.466666667	0	27.466666667	4.875605544	Mut
TCGA-J2-A4AE-01	0	35.966666667	0	35.966666667	4.877619178	Wt
TCGA-44-7662-01	0	7.266666667	0	7.266666667	4.890130378	Wt
TCGA-55-8616-01	0	1.6	0	1.6	4.891825946	Mut
TCGA-J2-8194-01	0	24.133333333	1	15.666666667	4.896258359	Wt
TCGA-73-A9RS-01	1	11.333333333	1	3.566666667	4.900164752	Wt
TCGA-78-7146-01	1	5.766666667	0	5.766666667	4.904183354	Wt
TCGA-50-5935-01	1	21.766666667	0	21.766666667	4.905568511	Wt
TCGA-55-8096-01	1	23.966666667	1	18.866666667	4.906464614	Mut
TCGA-44-2666-01	1	3.233333333	1	3.233333333	4.910745764	Wt
TCGA-78-7147-01	1	19.533333333	1	19.266666667	4.917487399	Mut
TCGA-93-7348-01	0	17.7	0	17.7	4.917819187	Wt
TCGA-44-2668-01	1	25.366666667	1	14.433333333	4.920347368	Wt
TCGA-55-8091-01	0	20	0	20	4.923928798	Wt
TCGA-67-4679-01	0	14.933333333	0	14.933333333	4.926101982	Wt
TCGA-05-4426-01	0	26.366666667	1	15.233333333	4.926528853	Wt
TCGA-44-6147-01	0	28.166666667	0	28.166666667	4.926685987	Wt
TCGA-62-A46V-01	0	73.3	0	73.3	4.930434697	Wt
TCGA-71-6725-01	0	8.533333333	1	5.466666667	4.930467475	Wt
TCGA-44-6775-01	0	23.5	1	22.8	4.93409749	Wt
TCGA-99-7458-01	0	24.9	0	24.9	4.937231542	Wt
TCGA-73-4668-01	0	15.566666667	1	7.866666667	4.940102504	Wt
TCGA-95-7562-01	1	2.9	0	2.9	4.941312391	Wt
TCGA-49-6767-01	0	22.566666667	0	22.566666667	4.94599395	Wt
TCGA-44-2662-01	0	42.666666667	1	8.166666667	4.950204361	Wt
TCGA-78-7143-01	1	165.366666667	1	50	4.95783246	Wt
TCGA-55-8092-01	1	5.133333333	1	4.233333333	4.961581376	Wt
TCGA-55-7914-01	1	6.233333333	1	6.233333333	4.964927327	Wt
TCGA-50-5946-02	0	53.9	1	7.366666667	4.965478611	Wt
TCGA-86-6562-01	1	12.533333333	1	9.133333333	4.97292279	Wt
TCGA-91-6830-01	0	2	1	0.6	4.978440943	Wt
TCGA-55-8207-01	0	32.566666667	0	32.566666667	4.979995586	Wt
TCGA-95-A4VK-01	0	21.7	1	16.533333333	4.980037705	Wt
TCGA-J2-A4AG-01	0	32.933333333	0	32.933333333	4.980259394	Wt
TCGA-44-2662-01	0	42.666666667	1	8.166666667	4.981981981	Wt
TCGA-83-5908-01	0	27.466666667	0	27.466666667	4.987142824	Wt
TCGA-78-7633-01	1	50.933333333	1	48.333333333	4.988940595	Wt
TCGA-05-4396-01	1	10.1	0	10.1	4.995638464	Wt
TCGA-50-5066-02	0	48.066666667	1	15.9	4.99638551	Wt
TCGA-44-4112-01	1	26.933333333	1	20.633333333	5.009607515	Wt
TCGA-MP-A4TF-01	1	11.2	1	6.5	5.015175569	Wt
TCGA-62-A46O-01	1	48.466666667	1	31.433333333	5.025343711	Wt
TCGA-67-3774-01	0	12.833333333	0	12.833333333	5.025970966	Wt
TCGA-44-6774-01	0	21.933333333	0	21.933333333	5.027356928	Wt
TCGA-55-6975-01	1	3.933333333	1	3.933333333	5.033260007	Wt
TCGA-91-6840-01	0	12.4	0	12.4	5.037101114	Wt

TCGA-44-6775-01	0	23.5	1	22.8	5.039657179	Wt
TCGA-86-8280-01	0	23.366666667	0	23.366666667	5.051187033	Mut
TCGA-NJ-A4YI-01	1	0.133333333	0	0.133333333	5.054954405	Wt
TCGA-38-4630-01	1	35.766666667	1	17.466666667	5.061509484	Wt
TCGA-78-7163-01	0	241.6	0	241.6	5.062946681	Wt
TCGA-75-6207-01	1		0	0	5.06711881	Wt
TCGA-55-8087-01	0	15.4	0	15.4	5.077504386	Wt
TCGA-86-8281-01	0	0	0	0	5.081685938	Wt
TCGA-44-2668-01	1	25.366666667	1	14.433333333	5.085091809	Wt
TCGA-80-5607-01	0		0	0	5.086150535	Wt
TCGA-49-6745-01	0	17.4	0	17.4	5.098734799	Wt
TCGA-J2-8192-01	0	24.633333333	1	16.066666667	5.099077915	Mut
TCGA-55-7724-01	0	23.5	0	23.5	5.102770593	Wt
TCGA-95-7948-01	0	15.866666667	0	15.866666667	5.108568658	Wt
TCGA-50-6673-01	1	0.733333333	0	0.733333333	5.110954241	Wt
TCGA-44-6779-01	1	16.666666667	1	7.733333333	5.112700679	Wt
TCGA-75-6203-01	0		0	0	5.115891511	Wt
TCGA-55-A57B-01	0	18.2	0	18.2	5.116611607	Mut
TCGA-62-A46R-01	1	57.5	0	57.5	5.117365529	Wt
TCGA-55-7726-01	0	21.733333333	0	21.733333333	5.120665786	Wt
TCGA-50-6595-01	1	6.3	1	6.066666667	5.128403301	Mut
TCGA-50-8457-01	0	37.5	0	37.5	5.132124884	Wt
TCGA-97-7554-01	0	25.833333333	0	25.833333333	5.136727641	Wt
TCGA-55-8614-01	0	17.866666667	0	17.866666667	5.136777443	Wt
TCGA-75-5125-01	1	67.566666667	1	58.4	5.138987579	Wt
TCGA-50-5933-01	1	79.766666667	0	79.766666667	5.140783573	Wt
TCGA-44-3917-01	0	39.433333333	0	39.433333333	5.141155493	Wt
TCGA-50-5939-01	1	15.333333333	0	15.333333333	5.145491623	Wt
TCGA-55-8511-01	0	18.4	1	15.333333333	5.150007862	Wt
TCGA-75-6206-01	0	86.333333333	0	86.333333333	5.153898434	Wt
TCGA-MP-A4TA-01	1	31.666666667	1	24.166666667	5.158569107	Wt
TCGA-44-A47A-01	0	15.533333333	1	13.233333333	5.163500786	Wt
TCGA-05-4410-01	0	0	0	0	5.165765243	Mut
TCGA-L9-A50W-01	1	14.733333333	1	12.966666667	5.196222253	Mut
TCGA-44-3918-01	0	34.533333333	1	17.033333333	5.198289209	Wt
TCGA-50-5944-01	0	58.333333333	0	58.333333333	4.206944192	Mut
TCGA-44-6147-01	0	28.166666667	0	28.166666667	5.233683289	Wt
TCGA-73-4677-01	1	1.266666667	1	1.266666667	5.235813704	Wt
TCGA-55-6543-01	0	14.5	0	14.5	5.237763492	Wt
TCGA-50-6591-01	1	3.966666667	1	3.966666667	5.250530939	Mut
TCGA-MP-A5C7-01	0	74.933333333	0	74.933333333	5.254997819	Wt
TCGA-86-8668-01	0	14.1	0	14.1	5.255279374	Mut
TCGA-69-7974-01	0	6.133333333	0	6.133333333	5.257465164	Wt
TCGA-44-A4SU-01	1	13.633333333	1	8.666666667	5.262213592	Mut
TCGA-05-4402-01	1	8.133333333	0	8.133333333	5.272714424	Mut
TCGA-44-8117-01	0	12.833333333	0	12.833333333	5.295968595	Wt
TCGA-86-7955-01	0	35.733333333	1	27.333333333	5.300652216	Wt
TCGA-86-8278-01	0	31.466666667	1	0.966666667	5.304885507	Wt
TCGA-44-8120-01	0	8.666666667	0	8.666666667	5.307858445	Wt
TCGA-97-8171-01	0	18.933333333	1	14.7	5.310662357	Mut
TCGA-78-7158-01	1	5.966666667	1	4.4	5.322298252	Mut
TCGA-97-7937-01	0	18.8	0	18.8	5.337336429	Wt
TCGA-44-5645-01	0	28.4	0	28.4	5.345560449	Mut
TCGA-05-4422-01	0	12.166666667	0	12.166666667	5.353010488	Wt
TCGA-44-6148-01	0	23.466666667	0	23.466666667	5.365960472	Wt
TCGA-55-6986-01	0	108.7	0	108.7	5.36936531	Wt
TCGA-55-7573-01	0	16.233333333	0	16.233333333	5.381536178	Wt
TCGA-50-5936-01	1	8.566666667	1	5	5.40318397	Wt
TCGA-86-8279-01	0	31.633333333	0	31.633333333	5.40688921	Wt
TCGA-49-6743-01	0	54.033333333	0	54.033333333	5.412471959	Mut
TCGA-78-7167-01	1	89.366666667	1	24.2	5.42796714	Wt
TCGA-44-5645-01	0	28.4	0	28.4	5.439696988	Mut
TCGA-91-6849-01	0	1.166666667	0	1.166666667	5.44018912	Wt

TCGA-44-6146-01	0	24.266666667	1	21.033333333	5.45198047	Wt
TCGA-73-4666-01	0	26.666666667	0	26.666666667	5.455258435	Wt
TCGA-55-1592-01	1	23.366666667	1	15.066666667	5.46364076	Wt
TCGA-55-A4DG-01	0	20.266666667	0	20.266666667	5.465035583	Wt
TCGA-62-8399-01	0	89.866666667	0	89.866666667	5.475540864	Wt
TCGA-44-6776-01	0	87.2	0	87.2	5.483390203	Wt
TCGA-78-7150-01	1	22.2	1	4.633333333	5.494285129	Wt
TCGA-97-7546-01	0	42.833333333	1	41.833333333	5.511744784	Wt
TCGA-86-7714-01	1	20.833333333	1	20.833333333	5.547016885	Wt
TCGA-55-8506-01	0	0.366666667	0	0.366666667	5.549529224	Mut
TCGA-78-7156-01	1	32.533333333	0	32.533333333	5.558827026	Wt
TCGA-50-5942-01	0	61.566666667	1	45.933333333	5.584576233	Wt
TCGA-05-4425-01	0	22.3	0	22.3	5.596131177	Wt
TCGA-50-5932-01	1	41.166666667	1	36.333333333	5.607266764	Wt
TCGA-05-5425-01	0	29.4	1	16.2	5.623740392	Wt
TCGA-91-6829-01	1	41.933333333	0	41.933333333	5.62959823	Wt
TCGA-99-8033-01	1	21.866666667	1	21.866666667	5.677005426	Wt
TCGA-97-7938-01	1	0.6	0	0.6	5.684384139	Wt
TCGA-44-6775-01	0	23.5	1	22.8	5.691767192	Wt
TCGA-44-2666-01	1	3.233333333	1	3.233333333	5.692384244	Wt
TCGA-78-7539-01	0	26.366666667	1	22.933333333	5.693512312	Wt
TCGA-MP-A4TC-01	1	2.466666667	0	2.466666667	5.750976962	Wt
TCGA-44-2662-01	0	42.666666667	1	8.166666667	5.760679281	Wt
TCGA-38-6178-01	0	14.933333333	0	14.933333333	5.781469551	Mut
TCGA-55-6712-01	1	5.7	1	5.7	5.796375897	Wt
TCGA-44-2656-01	0	47.633333333	1	18.933333333	5.838726573	Wt
TCGA-64-5775-01	1	2.066666667	1	2.066666667	5.844559767	Wt
TCGA-44-A47B-01	0	9.566666667	0	9.566666667	5.915979843	Wt
TCGA-05-4415-01	1	3.033333333	1	2	5.931133699	Wt
TCGA-44-5645-01	0	28.4	0	28.4	5.941481214	Mut
TCGA-55-7994-01	0	20.1	0	20.1	5.950084562	Wt
TCGA-44-6147-01	0	28.166666667	0	28.166666667	6.001354575	Wt
TCGA-97-8174-01	1	5.466666667	0	5.466666667	6.005339689	Wt
TCGA-78-7155-01	1	39.033333333	1	6.966666667	6.007145552	Mut
TCGA-53-7624-01	1	34.766666667	1	13.333333333	6.307631825	Wt
TCGA-44-2659-01	0	45.566666667	1	38.2	6.50191913	Wt
TCGA-95-7567-01	0	18.933333333	0	18.933333333	7.164023776	Wt

GSE11969

Accession	ch1_Status	ch1_Survival (days)	ch1_Survival (months)	ch1_EGFR status	YAP1
GSM303109	Dead	790	26.33333333	Wt	-0.324649708
GSM303020	Alive	2974	99.13333333	Wt	-0.233977529
GSM303108	Dead	229	7.633333333	Wt	-0.223820653
GSM303141	Dead	1862	62.06666667	Wt	-0.201105938
GSM302999	Alive	3214	107.1333333	Wt	-0.19752348
GSM303009	Dead	694	23.13333333	Wt	-0.171200198
GSM303120	Dead	1554	51.8	Wt	-0.167836592
GSM303019	Alive	3170	105.6666667	Wt	-0.160279019
GSM303073	Dead	1008	33.6	Wt	-0.157276402
GSM303006	Alive	3295	109.8333333	Mut	-0.137173457
GSM303008	Dead	1855	61.83333333	Mut	-0.135333272
GSM303074	Dead	1201	40.03333333	Wt	-0.130795899
GSM303130	Alive	2705	90.16666667	Wt	-0.126243339
GSM303011	Alive	3169	105.6333333	Wt	-0.121332452
GSM303142	Dead	683	22.76666667	Wt	-0.120912705
GSM303027	Alive	2701	90.03333333	Wt	-0.119236803
GSM303105	Dead	374	12.46666667	Wt	-0.10982187
GSM303084	Dead	906	30.2	Wt	-0.10482786
GSM303114	Dead	2196	73.2	Wt	-0.10278679
GSM303104	Alive	2521	84.03333333	Wt	-0.10274043
GSM303077	Alive	2335	77.83333333	Wt	-0.099263599
GSM303089	Alive	3066	102.2	Wt	-0.098220746
GSM303133	Dead	139	4.633333333	Wt	-0.093638022
GSM303010	Alive	3246	108.2	Wt	-0.090044185
GSM303038	Alive	2426	80.86666667	Wt	-0.085230525
GSM303129	Dead	888	29.6	Wt	-0.08347518
GSM303101	Dead	134	4.466666667	Wt	-0.083260055
GSM303059	Alive	2533	84.43333333	Wt	-0.079962391
GSM303004	Dead	511	17.03333333	Mut	-0.077426991
GSM303048	Dead	784	26.13333333	Wt	-0.076106321
GSM303024	Alive	2979	99.3	Wt	-0.072744404
GSM303005	Alive	3245	108.1666667	Wt	-0.071783302
GSM303013	Dead	1030	34.33333333	Wt	-0.07147457
GSM303015	Alive	3057	101.9	Wt	-0.063719969
GSM303116	Alive	3282	109.4	Wt	-0.062932703
GSM303102	Dead	1454	48.46666667	Wt	-0.062036225
GSM303087	Dead	309	10.3	Wt	-0.052860422
GSM303002	Alive	3245	108.1666667	Mut	-0.051561214
GSM303125	Dead	445	14.83333333	Wt	-0.048563399
GSM303046	Alive	2702	90.06666667	Wt	-0.048172444
GSM303119	Alive	2998	99.93333333	Wt	-0.047557714
GSM303052	Dead	1880	62.66666667	Mut	-0.042866666
GSM303121	Dead	232	7.733333333	Wt	-0.042249833

GSM303127	Alive	2886	96.2	Wt	-0.036058185
GSM303003	Alive	3274	109.1333333	Wt	-0.032193996
GSM303012	Alive	3161	105.3666667	Wt	-0.027490279
GSM302996	Dead	1326	44.2	Mut	-0.023526085
GSM303023	Alive	2873	95.76666667	Wt	-0.022961449
GSM303047	Alive	2456	81.86666667	Wt	-0.022224175
GSM303039	Dead	2193	73.1	Wt	-0.021113876
GSM303060	Alive	2528	84.26666667	Wt	-0.020294087
GSM303136	Dead	25	0.833333333	Wt	-0.019673782
GSM303131	Dead	2546	84.86666667	Wt	-0.017054788
GSM303056	Alive	2525	84.16666667	Wt	-0.016315786
GSM303138	Alive	2533	84.43333333	Wt	-0.016175976
GSM303090	Alive	3263	108.7666667	Wt	-0.013190756
GSM303134	Alive	2513	83.76666667	Wt	-0.011946265
GSM303036	Dead	397	13.23333333	Mut	-0.011434889
GSM303016	Alive	3182	106.0666667	Wt	-0.007211423
GSM303086	Dead	1425	47.5	Wt	-0.005334954
GSM303033	Dead	1389	46.3	Mut	-0.004446542
GSM303078	Dead	860	28.66666667	Mut	-0.000885413
GSM303070	Dead	777	25.9	Wt	0.001181344
GSM303122	Alive	2823	94.1	Wt	0.004609064
GSM303064	Alive	2521	84.03333333	Mut	0.004994578
GSM303028	Dead	896	29.86666667	Wt	0.005503052
GSM303113	Dead	1565	52.16666667	Wt	0.009732259
GSM303035	Dead	361	12.03333333	Mut	0.011829447
GSM303043	Alive	2702	90.06666667	Mut	0.012131993
GSM303007	Alive	3260	108.6666667	Mut	0.01327219
GSM303079	Dead	186	6.2	Wt	0.013592755
GSM303128	Alive	2718	90.6	Wt	0.014121735
GSM302997	Alive	3275	109.1666667	Wt	0.014762845
GSM303062	Alive	2507	83.56666667	Wt	0.0189041
GSM303029	Alive	2910	97	Wt	0.019932985
GSM303124	Dead	933	31.1	Wt	0.022456541
GSM303139	Alive	2358	78.6	Wt	0.024237149
GSM303094	Dead	660	22	Wt	0.025402346
GSM303082	Dead	867	28.9	Wt	0.025488267
GSM303041	Alive	2355	78.5	Mut	0.025736147
GSM303025	Alive	2905	96.83333333	Wt	0.028381052
GSM303014	Dead	2102	70.06666667	Wt	0.029452816
GSM302998	Dead	1687	56.23333333	Wt	0.030690891
GSM303115	Alive	2818	93.93333333	Wt	0.033665391
GSM303111	Alive	3596	119.8666667	Wt	0.034346107
GSM303081	Dead	1074	35.8	Mut	0.034718225
GSM303080	Alive	2342	78.06666667	Wt	0.035859159
GSM303069	Alive	2341	78.03333333	Wt	0.036871894
GSM303144	Dead	795	26.5	Wt	0.038730781

GSM303000	Dead	1200	40	Mut	0.039271062
GSM303034	Alive	2796	93.2	Mut	0.043488619
GSM303040	Alive	2797	93.23333333	Mut	0.045143877
GSM303068	Dead	1229	40.96666667	Wt	0.046323133
GSM303021	Alive	2879	95.96666667	Mut	0.047228183
GSM303017	Alive	3009	100.3	Wt	0.047624856
GSM303095	Dead	422	14.06666667	Wt	0.048620655
GSM303051	Dead	2347	78.23333333	Wt	0.049396292
GSM303118	Dead	764	25.46666667	Wt	0.049674733
GSM303123	Alive	2888	96.26666667	Wt	0.050683997
GSM303001	Dead	223	7.433333333	Mut	0.051302385
GSM303137	Dead	25	0.833333333	Wt	0.05327955
GSM303132	Dead	1463	48.76666667	Wt	0.054326086
GSM303110	Alive	3429	114.3	Wt	0.056676269
GSM303022	Dead	1271	42.36666667	Mut	0.057282031
GSM303018	Alive	3004	100.1333333	Wt	0.058542251
GSM303076	Dead	749	24.96666667	Mut	0.059432814
GSM303140	Dead	288	9.6	Wt	0.062272988
GSM303107	Alive	2353	78.43333333	Wt	0.063942865
GSM303099	Alive	2712	90.4	Wt	0.067072969
GSM303061	Alive	2353	78.43333333	Mut	0.067271037
GSM303097	Dead	297	9.9	Wt	0.070447699
GSM303072	Alive	2348	78.26666667	Mut	0.072585948
GSM303032	Alive	2932	97.73333333	Mut	0.07563788
GSM303044	Dead	636	21.2	Wt	0.076224185
GSM303058	Dead	1164	38.8	Mut	0.078273345
GSM303026	Dead	2885	96.16666667	Wt	0.07890779
GSM303112	Dead	220	7.333333333	Wt	0.080398359
GSM303066	Alive	2260	75.33333333	Wt	0.084180245
GSM303098	Dead	127	4.233333333	Wt	0.088234903
GSM303117	Alive	3238	107.9333333	Wt	0.088416756
GSM303106	Alive	2634	87.8	Wt	0.094132423
GSM303037	Alive	2506	83.53333333	Wt	0.094504531
GSM303050	Alive	2520	84	Mut	0.096207075
GSM303054	Alive	2072	69.06666667	Wt	0.096748399
GSM303083	Dead	304	10.13333333	Wt	0.097586115
GSM303075	Alive	1956	65.2	Mut	0.099328899
GSM303085	Dead	477	15.9	Wt	0.106873354
GSM303088	Dead	2428	80.93333333	Mut	0.109475888
GSM303065	Dead	1501	50.03333333	Mut	0.10996232
GSM303042	Alive	2729	90.96666667	Mut	0.109965402
GSM303030	Alive	2813	93.76666667	Wt	0.112454654
GSM303091	Alive	3238	107.9333333	Wt	0.118687158
GSM303126	Alive	2910	97	Wt	0.124921821
GSM303053	Dead	2050	68.33333333	Wt	0.127170577
GSM303045	Dead	828	27.6	Wt	0.127643425

GSM303103	Alive	2688	89.6	Mut	0.139801638
GSM303031	Alive	2749	91.63333333	Mut	0.144041145
GSM303093	Dead	198	6.6	Wt	0.152183724
GSM303071	Dead	1333	44.43333333	Wt	0.152822879
GSM303063	Alive	2519	83.96666667	Mut	0.159866826
GSM303096	Alive	2713	90.43333333	Wt	0.160884164
GSM303135	Dead	2528	84.26666667	Wt	0.170674668
GSM303067	Alive	2339	77.96666667	Wt	0.176505549
GSM303057	Dead	482	16.06666667	Wt	0.283147351
GSM303049	Dead	596	19.86666667	Wt	0.305468283
GSM303055	Dead	440	14.66666667	Wt	0.315016784
GSM303100	Alive	2681	89.36666667	Wt	0.442809561
GSM303092	Dead	75	2.5	Wt	0.528135463
GSM303143	Dead	683	22.76666667	Wt	0.701515401

GSE72094

Accession	Vital_status	Survival_time _in_days	Survival_time _in_months	EGFR_status	YAP1
GSM1854797	NA	NA	NA	Mut	9.099851
GSM1854798	Alive	1249	41.63333333	WT	9.226885
GSM1854799	Alive	1057	35.23333333	WT	8.814131
GSM1854800	Alive	1025	34.16666667	WT	8.87244
GSM1854801	Alive	895	29.83333333	WT	8.997569
GSM1854802	Dead	922	30.73333333	WT	9.506766
GSM1854803	Alive	959	31.96666667	WT	8.751716
GSM1854804	Alive	969	32.3	WT	8.565
GSM1854805	Alive	833	27.76666667	WT	8.88976
GSM1854806	Alive	891	29.7	WT	9.197149
GSM1854807	Dead	524	17.46666667	WT	8.73646
GSM1854808	Alive	1035	34.5	WT	9.383468
GSM1854809	Alive	43	1.433333333	WT	9.471216
GSM1854810	Alive	925	30.83333333	WT	7.731534
GSM1854811	Alive	1053	35.1	Mut	10.00251
GSM1854812	Alive	24	0.8	WT	8.968085
GSM1854813	Alive	825	27.5	WT	8.360703
GSM1854814	Alive	854	28.46666667	WT	8.860174
GSM1854815	Alive	820	27.33333333	WT	8.537586
GSM1854816	Alive	412	13.73333333	WT	8.313683
GSM1854817	Alive	297	9.9	WT	9.330482
GSM1854818	Alive	582	19.4	WT	8.958425
GSM1854819	Alive	28	0.933333333	WT	8.964916
GSM1854820	Alive	22	0.733333333	WT	8.566269
GSM1854821	Alive	1260	42	Mut	8.832343
GSM1854822	Alive	1402	46.73333333	WT	10.82722
GSM1854823	Alive	799	26.63333333	WT	9.455572
GSM1854824	Alive	NA	NA	Mut	9.75746
GSM1854825	Alive	1329	44.3	WT	9.16274
GSM1854826	Alive	1488	49.6	WT	9.234784
GSM1854827	Alive	579	19.3	WT	8.247477
GSM1854828	Alive	1186	39.53333333	WT	9.16601
GSM1854829	Alive	1151	38.36666667	WT	9.01817
GSM1854830	Alive	1323	44.1	WT	9.034584
GSM1854831	Dead	314	10.46666667	WT	8.672868
GSM1854832	Alive	1110	37	WT	9.706945
GSM1854833	Alive	1494	49.8	WT	8.673906
GSM1854834	Alive	1024	34.13333333	Mut	8.501016
GSM1854835	Alive	1134	37.8	WT	8.861037
GSM1854836	Dead	508	16.93333333	WT	8.800755
GSM1854837	Dead	46	1.533333333	WT	9.916493
GSM1854838	Dead	1231	41.03333333	WT	8.227887
GSM1854839	Alive	917	30.56666667	Mut	9.23688
GSM1854840	Dead	488	16.26666667	WT	8.603046
GSM1854841	Alive	730	24.33333333	WT	9.439354
GSM1854842	Dead	1079	35.96666667	WT	8.668938
GSM1854843	Alive	1127	37.56666667	Mut	9.55258
GSM1854844	Dead	193	6.433333333	WT	9.500429
GSM1854845	Dead	NA	NA	WT	8.571903
GSM1854846	Alive	833	27.76666667	WT	9.701096
GSM1854847	Alive	708	23.6	WT	8.694561
GSM1854848	Alive	1240	41.33333333	WT	8.583948
GSM1854849	Alive	1465	48.83333333	WT	9.33247
GSM1854850	Alive	1685	56.16666667	WT	9.507897
GSM1854851	Alive	1949	64.96666667	Mut	8.783205
GSM1854852	Alive	1569	52.3	Mut	9.30496
GSM1854853	Alive	275	9.166666667	WT	8.926117
GSM1854854	Alive	1507	50.23333333	Mut	9.533685
GSM1854855	Dead	307	10.23333333	WT	9.52222

GSM1854856	Dead	905	30.16666667	WT	8.031094
GSM1854857	Alive	1388	46.26666667	WT	9.190088
GSM1854858	Dead	NA	NA	WT	9.302686
GSM1854859	Dead	814	27.13333333	WT	7.838898
GSM1854860	Dead	436	14.53333333	WT	8.976737
GSM1854861	Dead	73	2.433333333	WT	10.65463
GSM1854862	Alive	1596	53.2	WT	8.87211
GSM1854863	Alive	1488	49.6	WT	9.349724
GSM1854864	Dead	398	13.26666667	Mut	9.344331
GSM1854865	Alive	1623	54.1	WT	7.824405
GSM1854866	Alive	1238	41.26666667	WT	8.680008
GSM1854867	Alive	1481	49.36666667	WT	9.015315
GSM1854868	Dead	430	14.33333333	WT	9.869303
GSM1854869	Alive	771	25.7	WT	9.108859
GSM1854870	Alive	1109	36.96666667	WT	9.078901
GSM1854871	Alive	1120	37.33333333	Mut	9.059004
GSM1854872	Alive	844	28.13333333	WT	8.912007
GSM1854873	Alive	1086	36.2	WT	8.718605
GSM1854874	Dead	871	29.03333333	WT	8.973937
GSM1854875	Alive	1063	35.43333333	WT	8.436293
GSM1854876	Alive	907	30.23333333	WT	6.939572
GSM1854877	Alive	776	25.86666667	WT	8.477135
GSM1854878	NA	NA	NA	Mut	8.988721
GSM1854879	Alive	974	32.46666667	WT	9.40172
GSM1854880	Alive	852	28.4	WT	9.097697
GSM1854881	Dead	732	24.4	WT	9.11721
GSM1854882	Alive	557	18.56666667	WT	9.605862
GSM1854883	Dead	1074	35.8	WT	8.635374
GSM1854884	Dead	328	10.93333333	WT	8.475591
GSM1854885	Dead	528	17.6	WT	9.701739
GSM1854886	Dead	415	13.83333333	WT	9.089432
GSM1854887	Alive	406	13.53333333	WT	8.897952
GSM1854888	Alive	840	28	WT	9.569073
GSM1854889	Dead	383	12.76666667	WT	8.509616
GSM1854890	Alive	1112	37.06666667	WT	9.133132
GSM1854891	Dead	442	14.73333333	WT	9.260076
GSM1854892	Dead	244	8.133333333	WT	9.249816
GSM1854893	Dead	3	0.1	WT	9.202608
GSM1854894	NA	NA	NA	WT	9.017601
GSM1854895	Dead	313	10.43333333	WT	8.947098
GSM1854896	Dead	1045	34.83333333	WT	7.975171
GSM1854897	Dead	347	11.56666667	WT	8.806149
GSM1854898	Alive	16	0.533333333	WT	9.429063
GSM1854899	Dead	131	4.366666667	WT	9.153653
GSM1854900	Alive	907	30.23333333	WT	7.781331
GSM1854901	NA	NA	NA	WT	7.878682
GSM1854902	Alive	27	0.9	WT	9.473649
GSM1854903	Dead	558	18.6	WT	8.970533
GSM1854904	Alive	1061	35.36666667	WT	8.195267
GSM1854905	NA	NA	NA	WT	7.494296
GSM1854906	Alive	1146	38.2	WT	9.802336
GSM1854907	Alive	1070	35.66666667	WT	9.28964
GSM1854908	Alive	1141	38.03333333	Mut	8.96191
GSM1854909	Alive	889	29.63333333	WT	8.999061
GSM1854910	Alive	1147	38.23333333	WT	8.529213
GSM1854911	Dead	999	33.3	WT	9.391904
GSM1854912	NA	NA	NA	WT	9.451543
GSM1854913	Alive	676	22.53333333	WT	8.661977
GSM1854914	NA	NA	NA	WT	9.227969
GSM1854915	Alive	140	4.666666667	WT	8.917343
GSM1854916	Alive	1153	38.43333333	WT	8.6891
GSM1854917	Alive	1239	41.3	Mut	9.590368
GSM1854918	Alive	1156	38.53333333	WT	9.114251

GSM1854919	Alive	982	32.73333333	Mut	8.704376
GSM1854920	Alive	950	31.66666667	WT	8.580546
GSM1854921	Dead	50	1.666666667	WT	9.029409
GSM1854922	Alive	1150	38.33333333	WT	7.937349
GSM1854923	Alive	1326	44.2	WT	8.443994
GSM1854924	Alive	124	4.133333333	Mut	8.881816
GSM1854925	Alive	268	8.933333333	WT	8.108461
GSM1854926	Alive	262	8.733333333	WT	8.162935
GSM1854927	Dead	76	2.533333333	WT	8.487679
GSM1854928	Alive	1239	41.3	Mut	8.708558
GSM1854929	Alive	683	22.76666667	WT	9.409146
GSM1854930	Alive	926	30.86666667	WT	9.177077
GSM1854931	Alive	956	31.86666667	WT	8.840576
GSM1854932	Alive	802	26.73333333	Mut	8.919307
GSM1854933	Alive	818	27.26666667	WT	8.508073
GSM1854934	Alive	849	28.3	WT	9.704236
GSM1854935	Dead	512	17.06666667	WT	9.226603
GSM1854936	Alive	962	32.06666667	WT	9.83335
GSM1854937	Alive	972	32.4	WT	9.230293
GSM1854938	Alive	79	2.633333333	WT	9.970903
GSM1854939	Alive	NA	NA	WT	9.518253
GSM1854940	Alive	947	31.56666667	WT	9.655503
GSM1854941	Alive	689	22.96666667	WT	9.763297
GSM1854942	Alive	527	17.56666667	WT	9.013743
GSM1854943	NA	NA	NA	WT	9.062817
GSM1854944	Alive	1166	38.86666667	WT	8.732088
GSM1854945	Alive	1984	66.13333333	WT	9.54887
GSM1854946	Alive	NA	NA	WT	9.154097
GSM1854947	Dead	281	9.366666667	WT	9.896821
GSM1854948	Dead	310	10.33333333	WT	9.665676
GSM1854949	Dead	NA	NA	Mut	9.112889
GSM1854950	Alive	874	29.13333333	WT	8.770766
GSM1854951	Dead	80	2.666666667	WT	8.182843
GSM1854952	Alive	1106	36.86666667	WT	9.08285
GSM1854953	Alive	1779	59.3	WT	9.508658
GSM1854954	Alive	996	33.2	WT	8.958061
GSM1854955	Alive	758	25.26666667	WT	9.757831
GSM1854956	Dead	460	15.33333333	WT	6.107579
GSM1854957	Dead	283	9.433333333	WT	9.297194
GSM1854958	Alive	921	30.7	WT	9.066954
GSM1854959	NA	NA	NA	WT	8.611041
GSM1854960	Alive	949	31.63333333	WT	9.636689
GSM1854961	Dead	113	3.766666667	WT	9.461845
GSM1854962	Alive	808	26.93333333	WT	9.295412
GSM1854963	Dead	35	1.166666667	WT	9.274907
GSM1854964	Alive	642	21.4	WT	9.092866
GSM1854965	Dead	680	22.66666667	WT	10.07548
GSM1854966	Dead	23	0.766666667	WT	8.917599
GSM1854967	Alive	924	30.8	WT	8.539295
GSM1854968	NA	NA	NA	WT	9.688819
GSM1854969	Alive	356	11.86666667	WT	8.955727
GSM1854970	Alive	392	13.06666667	WT	8.625811
GSM1854971	Alive	489	16.3	WT	8.842271
GSM1854972	Alive	919	30.63333333	WT	9.057262
GSM1854973	Alive	913	30.43333333	Mut	9.022705
GSM1854974	Alive	840	28	Mut	8.619756
GSM1854975	Alive	911	30.36666667	WT	8.662713
GSM1854976	NA	NA	NA	WT	8.652368
GSM1854977	Alive	692	23.06666667	WT	8.60133
GSM1854978	Dead	NA	NA	WT	8.621762
GSM1854979	Alive	751	25.03333333	WT	8.731385
GSM1854980	Alive	749	24.96666667	WT	8.138111
GSM1854981	Dead	591	19.7	WT	8.69628

GSM1854982	Alive	NA	NA	WT	9.284407
GSM1854983	Dead	116	3.866666667	WT	9.59548
GSM1854984	Alive	20	0.666666667	WT	8.549847
GSM1854985	Alive	687	22.9	WT	8.992447
GSM1854986	Alive	577	19.23333333	WT	9.889183
GSM1854987	Alive	937	31.23333333	Mut	9.05373
GSM1854988	Alive	785	26.16666667	WT	8.825728
GSM1854989	Alive	962	32.06666667	WT	9.205563
GSM1854990	Alive	830	27.66666667	Mut	9.883342
GSM1854991	Alive	NA	NA	WT	8.800814
GSM1854992	Alive	554	18.46666667	WT	8.873801
GSM1854993	NA	NA	NA	WT	9.508975
GSM1854994	Alive	905	30.16666667	Mut	9.283173
GSM1854995	Dead	496	16.53333333	WT	8.664582
GSM1854996	Dead	651	21.7	WT	9.583513
GSM1854997	Dead	600	20	WT	10.15957
GSM1854998	Dead	852	28.4	WT	9.766775
GSM1854999	Alive	897	29.9	WT	8.473205
GSM1855000	Alive	807	26.9	WT	9.234313
GSM1855001	Dead	487	16.23333333	WT	8.88496
GSM1855002	NA	NA	NA	WT	8.498395
GSM1855003	Alive	848	28.26666667	WT	8.401257
GSM1855004	Alive	1029	34.3	WT	8.758684
GSM1855005	Alive	977	32.56666667	WT	8.833255
GSM1855006	Alive	988	32.93333333	WT	9.146095
GSM1855007	Alive	1130	37.66666667	Mut	8.584954
GSM1855008	Alive	823	27.43333333	WT	8.716101
GSM1855009	Alive	756	25.2	WT	8.774098
GSM1855010	Alive	681	22.7	WT	8.172714
GSM1855011	Alive	1012	33.73333333	WT	8.773838
GSM1855012	Alive	1079	35.96666667	WT	9.684117
GSM1855013	NA	NA	NA	WT	8.094474
GSM1855014	Dead	1119	37.3	WT	8.738934
GSM1855015	Alive	868	28.93333333	WT	8.774109
GSM1855016	Alive	994	33.13333333	Mut	9.233771
GSM1855017	Dead	NA	NA	WT	10.41744
GSM1855018	Alive	878	29.26666667	WT	8.41826
GSM1855019	Dead	358	11.93333333	WT	8.457507
GSM1855020	Alive	1189	39.63333333	WT	8.592641
GSM1855021	Alive	890	29.66666667	WT	8.014189
GSM1855022	Alive	831	27.7	WT	8.87846
GSM1855023	Alive	1174	39.13333333	WT	8.528942
GSM1855024	Alive	1006	33.53333333	WT	8.625874
GSM1855025	Alive	NA	NA	WT	8.916119
GSM1855026	Alive	1038	34.6	WT	8.303096
GSM1855027	Dead	837	27.9	WT	9.332812
GSM1855028	Alive	911	30.36666667	WT	7.914101
GSM1855029	Dead	780	26	WT	8.45879
GSM1855030	NA	NA	NA	Mut	9.215962
GSM1855031	Alive	1033	34.43333333	WT	8.872519
GSM1855032	Alive	818	27.26666667	Mut	9.510533
GSM1855033	Alive	1011	33.7	Mut	9.832842
GSM1855034	Alive	809	26.96666667	WT	8.889399
GSM1855035	Alive	1074	35.8	WT	9.25587
GSM1855036	Dead	932	31.06666667	WT	8.947606
GSM1855037	Alive	1057	35.23333333	WT	8.4499
GSM1855038	Alive	871	29.03333333	WT	9.264548
GSM1855039	Dead	267	8.9	WT	9.553451
GSM1855040	Alive	807	26.9	WT	8.339844
GSM1855041	Alive	373	12.43333333	WT	8.697659
GSM1855042	Alive	937	31.23333333	WT	9.083464
GSM1855043	Alive	1029	34.3	WT	8.747866
GSM1855044	Alive	987	32.9	WT	9.444254

GSM1855045	Alive	860	28.66666667	WT	8.702441
GSM1855046	Alive	939	31.3	WT	9.77538
GSM1855047	Alive	1019	33.96666667	WT	8.359845
GSM1855048	Dead	652	21.73333333	WT	9.538899
GSM1855049	Dead	219	7.3	WT	8.510384
GSM1855050	Alive	698	23.26666667	WT	8.647972
GSM1855051	Alive	622	20.73333333	WT	9.437373
GSM1855052	Dead	951	31.7	WT	8.984037
GSM1855053	Alive	1015	33.83333333	Mut	9.891387
GSM1855054	Alive	900	30	WT	9.669322
GSM1855055	Alive	1076	35.86666667	WT	8.736675
GSM1855056	Alive	1061	35.36666667	WT	9.914101
GSM1855057	Alive	76	2.533333333	WT	9.790379
GSM1855058	NA	NA	NA	WT	8.657075
GSM1855059	Alive	588	19.6	Mut	9.292607
GSM1855060	Alive	1033	34.43333333	WT	8.775507
GSM1855061	Dead	759	25.3	WT	8.810337
GSM1855062	Dead	600	20	WT	9.045363
GSM1855063	Dead	349	11.63333333	WT	9.364958
GSM1855064	Alive	980	32.66666667	WT	9.518323
GSM1855065	Alive	724	24.13333333	WT	8.449454
GSM1855066	Alive	997	33.23333333	WT	9.303325
GSM1855067	Alive	987	32.9	WT	9.055422
GSM1855068	Dead	1031	34.36666667	WT	8.662406
GSM1855069	Alive	305	10.16666667	WT	9.617204
GSM1855070	Alive	994	33.13333333	Mut	9.331642
GSM1855071	Alive	597	19.9	WT	8.513405
GSM1855072	Alive	679	22.63333333	WT	8.838404
GSM1855073	Alive	1042	34.73333333	WT	9.152665
GSM1855074	Alive	733	24.43333333	WT	9.426005
GSM1855075	Alive	897	29.9	WT	9.087544
GSM1855076	Alive	917	30.56666667	WT	8.974595
GSM1855077	Alive	855	28.5	WT	9.144412
GSM1855078	Alive	882	29.4	WT	8.949082
GSM1855079	Dead	336	11.2	WT	8.872517
GSM1855080	Alive	671	22.36666667	WT	8.76047
GSM1855081	Alive	777	25.9	WT	10.05013
GSM1855082	Alive	595	19.83333333	WT	9.25394
GSM1855083	Alive	832	27.73333333	WT	8.991131
GSM1855084	Alive	919	30.63333333	WT	9.956692
GSM1855085	Alive	971	32.36666667	WT	9.433555
GSM1855086	Alive	882	29.4	WT	10.08199
GSM1855087	Alive	938	31.26666667	WT	9.426887
GSM1855088	Alive	468	15.6	WT	9.529674
GSM1855089	Alive	834	27.8	WT	9.487739
GSM1855090	NA	NA	NA	Mut	9.562624
GSM1855091	Dead	936	31.2	WT	8.706697
GSM1855092	Alive	732	24.4	WT	8.869249
GSM1855093	Alive	766	25.53333333	WT	8.576382
GSM1855094	Alive	607	20.23333333	Mut	8.741239
GSM1855095	Alive	578	19.26666667	WT	9.703231
GSM1855096	Alive	816	27.2	WT	7.000216
GSM1855097	Alive	853	28.43333333	WT	8.510905
GSM1855098	Alive	759	25.3	WT	8.627658
GSM1855099	Dead	917	30.56666667	WT	9.505332
GSM1855100	Alive	868	28.93333333	WT	8.992259
GSM1855101	Dead	920	30.66666667	Mut	9.224189
GSM1855102	Alive	607	20.23333333	WT	8.829236
GSM1855103	Alive	568	18.93333333	WT	7.199698
GSM1855104	Dead	98	3.266666667	WT	7.715828
GSM1855105	Alive	555	18.5	Mut	8.608009
GSM1855106	Dead	306	10.2	WT	9.21821
GSM1855107	Alive	906	30.2	WT	9.068145

GSM1855108	Alive	918	30.6	WT	9.637703
GSM1855109	Dead	693	23.1	WT	9.171387
GSM1855110	Alive	680	22.66666667	Mut	8.693938
GSM1855111	Alive	539	17.96666667	WT	9.625321
GSM1855112	Alive	820	27.33333333	WT	8.302545
GSM1855113	Alive	750	25	Mut	9.606819
GSM1855114	Alive	708	23.6	WT	9.150829
GSM1855115	NA	NA	NA	WT	8.583315
GSM1855116	Alive	672	22.4	WT	8.446901
GSM1855117	Alive	470	15.66666667	WT	9.533998
GSM1855118	Alive	54	1.8	WT	9.049505
GSM1855119	Alive	780	26	WT	8.694143
GSM1855120	Dead	216	7.2	WT	8.569882
GSM1855121	Dead	294	9.8	WT	8.090837
GSM1855122	Alive	757	25.23333333	Mut	8.211009
GSM1855123	Alive	760	25.33333333	WT	8.615807
GSM1855124	Alive	1089	36.3	WT	9.289232
GSM1855125	Dead	17	0.566666667	WT	8.826455
GSM1855126	Alive	577	19.23333333	WT	8.883692
GSM1855127	Alive	539	17.96666667	WT	8.821817
GSM1855128	Alive	906	30.2	WT	9.050434
GSM1855129	Alive	628	20.93333333	WT	9.00211
GSM1855130	Alive	NA	NA	WT	9.451548
GSM1855131	Dead	696	23.2	WT	9.454125
GSM1855132	Alive	858	28.6	WT	9.17906
GSM1855133	Alive	701	23.36666667	WT	8.591083
GSM1855134	Dead	382	12.73333333	WT	8.375997
GSM1855135	Alive	539	17.96666667	WT	8.716868
GSM1855136	Dead	4	0.133333333	WT	8.210296
GSM1855137	Alive	557	18.56666667	WT	9.187701
GSM1855138	Alive	619	20.63333333	WT	9.087733
GSM1855139	Alive	NA	NA	WT	7.992938
GSM1855140	Alive	830	27.66666667	WT	8.812813
GSM1855141	Alive	821	27.36666667	WT	9.898284
GSM1855142	Alive	831	27.7	WT	9.163264
GSM1855143	Alive	591	19.7	WT	8.165349
GSM1855144	Alive	850	28.33333333	WT	8.517413
GSM1855145	Dead	809	26.96666667	WT	8.694493
GSM1855146	Alive	142	4.733333333	WT	9.377129
GSM1855147	Alive	807	26.9	WT	8.860173
GSM1855148	Alive	NA	NA	WT	9.452085
GSM1855149	Alive	832	27.73333333	WT	8.973953
GSM1855150	Dead	98	3.266666667	WT	8.609361
GSM1855151	Alive	798	26.6	WT	9.241076
GSM1855152	Alive	799	26.63333333	WT	9.44518
GSM1855153	Dead	325	10.83333333	WT	9.274659
GSM1855154	NA	NA	NA	WT	7.973678
GSM1855155	Alive	NA	NA	WT	8.687348
GSM1855156	Alive	978	32.6	WT	8.263017
GSM1855157	Alive	806	26.86666667	WT	9.145962
GSM1855158	Alive	698	23.26666667	WT	9.080448
GSM1855159	Alive	861	28.7	Mut	10.09757
GSM1855160	Alive	325	10.83333333	WT	9.429445
GSM1855161	Dead	332	11.06666667	WT	8.453652
GSM1855162	Alive	734	24.46666667	Mut	10.20527
GSM1855163	Alive	867	28.9	WT	8.668473
GSM1855164	Alive	830	27.66666667	WT	8.580675
GSM1855165	Dead	541	18.03333333	WT	8.866504
GSM1855166	Alive	679	22.63333333	WT	8.698965
GSM1855167	Alive	NA	NA	WT	9.508729
GSM1855168	Dead	40	1.333333333	WT	8.926149
GSM1855169	Alive	740	24.66666667	WT	8.394851
GSM1855170	Alive	540	18	WT	9.459166

GSM1855171	Alive	1155	38.5	WT	9.281847
GSM1855172	Alive	797	26.56666667	WT	8.856476
GSM1855173	Dead	676	22.53333333	WT	9.030521
GSM1855174	Alive	540	18	WT	9.576768
GSM1855175	Dead	380	12.66666667	WT	8.231805
GSM1855176	Alive	1350	45	WT	9.352592
GSM1855177	Alive	570	19	Mut	8.383839
GSM1855178	Dead	58	1.933333333	WT	9.819593
GSM1855179	Alive	5	0.166666667	WT	8.745003
GSM1855180	Alive	554	18.46666667	WT	9.465681
GSM1855181	Alive	534	17.8	WT	8.858136
GSM1855182	Alive	4	0.133333333	Mut	9.587959
GSM1855183	Alive	1692	56.4	WT	8.95765
GSM1855184	Dead	223	7.433333333	WT	8.710357
GSM1855185	Alive	1085	36.16666667	WT	9.119427
GSM1855186	Dead	NA	NA	WT	9.621825
GSM1855187	Dead	912	30.4	WT	10.11495
GSM1855188	Dead	1357	45.23333333	WT	9.437567
GSM1855189	Dead	844	28.13333333	WT	9.163622
GSM1855190	Dead	520	17.33333333	WT	8.952723
GSM1855191	Dead	NA	NA	WT	9.866543
GSM1855192	Dead	239	7.966666667	WT	9.374456
GSM1855193	Alive	349	11.63333333	WT	9.011456
GSM1855194	NA	NA	NA	WT	8.227216
GSM1855195	Dead	1025	34.16666667	WT	10.06806
GSM1855196	Dead	1560	52	WT	9.314643
GSM1855197	Dead	NA	NA	WT	8.9792
GSM1855198	Dead	564	18.8	WT	9.32724
GSM1855199	Dead	293	9.766666667	WT	9.282148
GSM1855200	Dead	1053	35.1	WT	8.366611
GSM1855201	NA	NA	NA	WT	9.144288
GSM1855202	Dead	627	20.9	WT	9.836526
GSM1855203	Alive	395	13.16666667	WT	9.54534
GSM1855204	Dead	1007	33.56666667	Mut	9.53024
GSM1855205	Alive	NA	NA	WT	8.681203
GSM1855206	Dead	681	22.7	WT	8.651549
GSM1855207	Alive	1293	43.1	WT	9.796939
GSM1855208	Dead	1293	43.1	WT	9.238604
GSM1855209	Dead	968	32.26666667	WT	5.572981
GSM1855210	Alive	1843	61.43333333	WT	8.823246
GSM1855211	Alive	1616	53.86666667	WT	8.655076
GSM1855212	Alive	1559	51.96666667	WT	9.243187
GSM1855213	Alive	1703	56.76666667	WT	8.893663
GSM1855214	Alive	1834	61.13333333	WT	10.01138
GSM1855215	Alive	1551	51.7	WT	9.209234
GSM1855216	Alive	NA	NA	WT	9.078441
GSM1855217	Alive	1554	51.8	Mut	9.426236
GSM1855218	Dead	489	16.3	WT	9.467519
GSM1855219	Dead	216	7.2	WT	9.652226
GSM1855220	Alive	1510	50.33333333	WT	9.490726
GSM1855221	Alive	701	23.36666667	WT	9.392711
GSM1855222	Alive	1363	45.43333333	WT	8.767084
GSM1855223	Dead	1135	37.83333333	WT	9.139396
GSM1855224	Dead	618	20.6	WT	9.321449
GSM1855225	Dead	1037	34.56666667	WT	8.810958
GSM1855226	Alive	1599	53.3	WT	9.337531
GSM1855227	NA	NA	NA	WT	8.922146
GSM1855228	Dead	171	5.7	WT	9.167542
GSM1855229	Alive	2077	69.23333333	WT	8.253573
GSM1855230	Alive	1652	55.06666667	WT	9.473641
GSM1855231	Dead	489	16.3	WT	9.075749
GSM1855232	Dead	762	25.4	WT	9.312448
GSM1855233	Dead	NA	NA	WT	9.164859

GSM1855234	Alive	1693	56.43333333	WT	10.2286
GSM1855235	Dead	713	23.76666667	Mut	9.342579
GSM1855236	Alive	1652	55.06666667	WT	8.910212
GSM1855237	Dead	181	6.033333333	WT	9.512869
GSM1855238	Dead	1629	54.3	WT	8.560154

Immune infiltration

	B cell naive	B cell memory	B cell plasma	T cell CD8+	T cell CD4+ naive	T cell CD4+ memory resting	T cell CD4+ memory activated	T cell follicular helper	T cell regulatory (Tregs)	T cell gamma delta	NK cell resting	NK cell activated	Monocyte	Macrophage M0	Macrophage M1	Macrophage M2	Myeloid dendritic cell resting	Myeloid dendritic cell activated	Mast cell activated	Mast cell resting	Eosinophil	Neutrophil	Groups
TCGA-71-8520-01	0	0.00874063	0.044472288	0.00744039	0	0.11280548	0	0.047234316	0.02788878	0.00E+00	0.023302218	0.016664818	0.013418395	2.43E-01	0.034276828	0.28492971	0	0.078741063	0.053661731	0	0	0.003108305	YAP1_High
TCGA-05-4402-01	0.01818205	0.000647203	0.095415049	0.04294095	0	0.10450356	0	0.030025231	0.030628037	0.00E+00	0	0.019899863	0.009169463	4.31E-02	0.074648764	0.41339453	0.01061388	0.030258705	0.050364417	0	0	0.026219973	YAP1_High
TCGA-78-7147-01	0.03818265	0	0	0.09023056	0	0.10747709	0	0.062308419	0.070547334	1.02E-02	0	0.039146969	0.025257831	1.20E-02	0.231063053	0.18922143	0.015702763	0.014456439	0.094172379	0	0	0	YAP1_High
TCGA-86-8075-01	0.011961069	0.000774008	0.119637311	0.0134082	0	0.12597729	0	0.039233763	0.014906662	0.00E+00	0	0.020480935	0.005268612	9.57E-02	0.061809491	0.37365029	0.005525083	0.0394747	0	0.044183241	0	0.028002537	YAP1_High
TCGA-05-4410-01	0	0.041135969	0.216490093	0.15704887	0	0.1066935	0.001866357	0.075832675	0	0.00E+00	0	0.026910849	0.019526062	3.04E-02	0.118029434	0.19368813	0	0	0.008920427	0	0	0.003436514	YAP1_High
TCGA-78-7155-01	0.034988085	0	0.005572699	0.18723033	0	0.08898716	0.042606526	0.057815256	0	2.92E-02	0	0.041766761	0.019180809	0.00E+00	0.093766311	0.34798717	0.002377489	0	0	0.048508891	0	0	YAP1_High
TCGA-75-6207-01	0	0.002676425	0.031616077	0.06428703	0	0.12994747	0	0.026197595	0.027741832	0.00E+00	0	0.00570096	0.058539938	1.94E-01	0.008444073	0.36653297	0.004783161	0.038521768	0.025853767	0	0	0.015215801	YAP1_High
TCGA-05-4382-01	0	0.008003789	0.11380273	0.03030642	0	0.06125619	0.002721309	0.031539432	0.02209678	0.00E+00	0	0.016799746	0	1.44E-01	0.049249653	0.45723941	0	0	0	0.035179929	0	0.028065012	YAP1_High
TCGA-49-4501-01	0.007152985	0.061731066	0.088129805	0.05325917	0	0.22818577	0	0.053162035	0.015315512	1.16E-02	0	0.041994364	0.032306686	0.00E+00	0.033521694	0.26100373	0.016754819	0.039701297	0.054250767	0	0	0.001956619	YAP1_High
TCGA-55-6981-01	0.052577377	0.014868665	0.126172149	0.01042232	0	0.1109819	0.003307028	0.054143474	0.009974795	0.00E+00	0.006815593	0.003398598	0.041411465	7.04E-02	0	0.2402699	0.08870142	0.060819859	0.100401011	0	0	0.005367624	YAP1_High
TCGA-67-6217-01	0	0.0729291	0.125882948	0.13884937	0	0.14539606	0	0.035915125	0.035794629	0.00E+00	0	0.029661136	0.017456268	0.00E+00	0.051291434	0.23024436	0.063710679	0.014174702	0.03590212	0	0	0.002792066	YAP1_High
TCGA-L9-A50W-	0.006048545	0	0.116172026	0.03724837	0	0.20344718	0	0.050168319	0.02114214	0.00E+00	0	0.057020942	0.018106346	0.00E+00	0.056521195	0.17320137	0.147965078	0	0.111422168	0	0	0.001536332	YAP1_High
TCGA-86-8055-01	0.004328019	0	0.13106745	0.03490869	0	0.13695333	0	0.011937256	0.019592611	0.00E+00	0	0.012860794	0.010830034	1.87E-01	0.077032629	0.29179891	0.00591904	0.008929394	0.018676122	0.017603432	0	0.030759694	YAP1_High
TCGA-55-8206-01	0	0.011538145	0.030474838	0.03169469	0	0.08840729	0.001047824	0.019351815	0.019746735	0.00E+00	0	0.016460774	0.353927513	0.00E+00	0	0.18078978	0.089914197	0.010488502	0.146157898	0	0	0	YAP1_High
TCGA-62-8402-01	0.02109536	0	0	0.06455445	0	0.11957312	0.023665312	0.034463866	0.014946193	8.16E-03	0	0.086415647	0	1.77E-01	0.289410523	0.09573166	0.019018424	0	0.036016203	0	0	0.010355231	YAP1_High
TCGA-78-7158-01	0	0.033101098	0.019896042	0.02870459	0	0.23561645	0	0.015850248	0.001654285	0.00E+00	0	0.039492174	0.055023497	4.91E-03	0.039316161	0.25488134	0.061092488	0.016446284	0.194014524	0	0	0	YAP1_High
TCGA-55-8616-01	0.065732962	0.086160595	0.125978817	0.11503339	0	0.10845567	0	0.024034678	0.032148081	3.50E-03	0	0.002072624	0.024853373	0.00E+00	0.031488252	0.3441266	0	0.00372195	0.032693308	0	0	0	YAP1_High
TCGA-95-7947-01	0.041264001	0	0.053745227	0.1019101	0	0.22548593	0	0.07860829	0.006138197	0.00E+00	0	0.02115574	0.009903307	8.13E-03	0.122180556	0.2821113	0	0.002348572	0.043078056	0	0	0.003945098	YAP1_High
TCGA-67-3770-01	0.009716429	0	0.034317111	0.05026408	0	0.17692553	0	0.029756092	0	3.79E-02	0	0.030467837	0.01997083	0.00E+00	0.028149681	0.38204098	0.016348056	0.060828606	0.114205297	0	0	0.009148825	YAP1_High
TCGA-49-6743-01	0.038985466	0	0.137109022	0.08238572	0	0.13257842	0	0.033118838	0.020826344	0.00E+00	0.001797574	0.003183153	0.005304086	1.41E-01	0.081227698	0.29708433	0	0	0	0.018536062	0	0.006657642	YAP1_High
TCGA-97-8171-01	0	0.045874165	0.057119874	0.01551935	0	0.04522724	0	0.064725156	0	0.00E+00	0	0.029649859	0.014471168	1.80E-01	0.013945923	0.4855779	0	0.010838084	0.037464027	0	0	0	YAP1_High
TCGA-44-A4SU-	0.005085007	0.097991658	0.175835232	0.04110507	0	0.10751906	0	0.110033749	0.057468737	0.00E+00	0	0.030982604	0	8.15E-02	0.080568557	0.13379632	0.031344929	0	0.046728391	0	0	0	YAP1_High
TCGA-55-A57B-01	0.015491377	0	0	0.0475831	0	0.19495356	0	0.013096634	0.02721528	0.00E+00	0	0.052721328	0.074354687	0.00E+00	0.011380134	0.30291077	0.134578784	0.041707588	0.081622781	0	0	0.002383988	YAP1_High
TCGA-44-5645-01	0.002270624	0.0579762	0.028934426	0.05358019	0	0.27560572	0	0.029931654	0.054028705	0.00E+00	0	0.009591084	0.054151611	0.00E+00	0.02356306	0.22622218	0.104453564	0.026258118	0.053432869	0	0	0	YAP1_High
TCGA-50-6591-01	0.001782301	0	0.049880695	0.025673	0	0.1529385	0	0.022763191	0.024388123	0.00E+00	0.044396737	0	0	5.60E-01	0.010963085	0.02657473	0.018253178	0	0	0.062312738	0	0	YAP1_High
TCGA-95-7039-01	0.014434907	0	0.022563721	0.12161935	0	0.0928469	0	0.096418789	0.005960481	2.09E-02	0	0.021645045	0	1.12E-01	0.140123978	0.31988409	0.009479649	0	0.014490129	0.007848404	0	0	YAP1_High
TCGA-50-6595-01	0.007521651	0	0.016159542	0.07678719	0	0.08608689	0.00060645	0.0275228	0.01092668	0.00E+00	0.035472615	0.001831192	0	2.11E-01	0.160341081	0.36313752	0	0	0	0.002855288	0	0	YAP1_High
TCGA-55-8506-01	0	0.005771964	0.061485498	0.06083962	0	0.03280598	0	0.063302544	0.000762409	5.32E-03	0	0.029442499	0.03470616	3.05E-01	0.051098107	0.31084019	0.001030603	0	0	0.03739933	0	0	YAP1_High
TCGA-55-7570-01	0	0	0.243582735	0.15502679	0	0	0.01270362	0.154224812	0	0.00E+00	0	0.040313404	0.025479096	1.09E-01	0.023130978	0.20253061	0.000287565	0	0	0.033438236	0	0	YAP1_High
TCGA-86-8280-01	0	0.037081096	0.061268562	0.05757953	0	0.17475689	0	0.082583175	0.033402061	0.00E+00	0	0.026389056	0.028182184	4.54E-02	0.11053867	0.23510503	0.039341682	0.011930695	0.056413301	0	0	0	YAP1_High
TCGA-97-A4M1-01	0.010142409	0.003335997	0	0.07198721	0	0.10584957	0	0.03244254	0.035679661	0.00E+00	0.03597631	0.020179857	0.124676259	3.60E-02	0.022949079	0.30475697	0.012799909	0.0102335	0.17294426	0	0	0	YAP1_High
TCGA-67-3771-01	0.009833179	0	0.116435737	0.09467766	0	0.13250914	0.018701978	0.035997183	0.007005173	0.00E+00	0	0.011086698	0.032962455	8.02E-02	0.105731989	0.33781651	0	0	0.01511025	0	0	0.001919956	YAP1_High
TCGA-69-7765-01	0	0.046679115	0.162334221	0.06641211	0	0.13455046	0.047517868	0.050629679	0.003534199	0.00E+00	0.015800621	0	0.004267339	7.24E-02	0.062590031	0.1927835	0.116789281	0.010983301	0.01155456	0.001184878	0	0	YAP1_High
TCGA-55-8096-01	0	0.020210032	0.133592119	0.03915614	0	0.07446725	0	0	0.018674168	0.00E+00	0	0.007497775	0.026210341	2.69E-01	0.026828895	0.34924826	0	0	0.035219667	0	0	0	YAP1_High
TCGA-38-6178-01	0.013158035	0.007275998	0.190406751	0.03600359	0	0.17411002	0.002075027	0.001846164	0	0.00E+00	0.018625801	0	0.007168405	3.17E-03	0.051026567	0.35434224	0	0.053205927	0.087587731	0	0	0	YAP1_High
TCGA-97-A4M6-01	0.012768964	0.046822673	0	0.05877461	0	0.20888576	0	0.084256649	0.056786205	0.00E+00	0	0.03234048	0.028628885	7.16E-02	0.104929998	0.21824178	0.020941257	0	0.052568567	0	0	0.002502503	YAP1_High
TCGA-62-8394-01	0.036897524	0	0.060261548	0.04058584	0	0.23291037	0	0.042000898	0.025755337	0.00E+00	0.00666342	0.006960342	0.020295239	1.80E-01	0.075500798	0.20304653	0	0.059865055	0.008942521	0	0	0	YAP1_High
TCGA-93-A4JP-01	0.007829794	0	0	0.02995935	0	0.08764099	0	0.043672024	0	0.00E+00	0.005084495	0.01974763	0.044133153	9.67E-02	0.055882193	0.39263706	0	0.093536173	0.117800392	0	0	0.005328565	YAP1_High
TCGA-J2-8192-01	0	0.014320324	0.027230846	0.01801822	0	0.23709078	0	0.005316681	0	0.00E+00	0	0.01739943	0.029443199	1.02E-01	0.015345291	0.36146388	0.082722943	0.026984513	0.052138216	0	0	0.010070489	YAP1_High
TCGA-50-5944-01	0.036216156	0	0.023849213	0.0331304	0	0.12669554	0	0.017346312	0.00114291	0.00E+00	0	0.025786297	0.080551002	5.60E-02	0.028052936	0.39365055	0.003962626	0	0.173597409	0	0	0	YAP1_High
TCGA-MP-A4T9-	0.010390379	0.025611603	0	0.09517123	0	0.11610841	0	0.060593792	0.016773733	0.00E+00	0	0.042867093	0.054669168	4.80E-02	0.03107934	0.34922277	0.014289125	0.022778807	0.106596385	0	0	0.005891151	YAP1_High
TCGA-38-4627-01	0.002639412	0	0.038926234	0.02933086	0	0.19852321	0	0	0	0.00E+00	0	0.020765184	0.03877708	0.00E+00	0.035133913	0.55904173	0.004366984	0	0.066564534	0	0	0.005930851	YAP1_High
TCGA-67-3772-01	0.031715223	0	0.088477074	0.05106268	0	0.21263169	0	0.031924054	0.005367725	0.00E+00	0	0	0.077971551	0.00E+00	0.024525077	0.30375953	0.084916276	0.029408194	0.05343292	0	0	0.004807997	YAP1_High
TCGA-86-8668-01	0.006298414	0.040273063	0.091847704	0.06415845	0	0.15278876	0	0.061442672	0.032445238	0.00E+00	0	0.04650606	0.023397805	1.92E-02	0.05942804	0.28018358	0.024627468	0	0.097382322	0	0	0	YAP1_High
TCGA-MP-A4SW-01	0.001596262	0.049945165	0.126705707	0.07981789	0	0.15867296	0	0.042886925	0.062064602	0.00E+00	0.00571515	0.0245263	0.040846297	4.64E-02	0.017999933	0.26887924	0.008440356	0.014389048	0.049619787	0	0	0.001524067	YAP1_Low
TCGA-97-8547-01	0.005216376	0	0	0.01917804	0	0.0236404	0	0.051480885	0.077339769	0.00E+00	0	0	0.002993427	4.20E-01	0	0.31873406	0	0.051859146	0.021912933	0.007755313	0	0	YAP1_Low
TCGA-86-8074-01	0.013499078	0	0.037293583	0.06507659	0	0.08580467	0	0.069938662	0.032601547	0.00E+00	0	0.066645657	0.02103249	7.15E-02	0.103915655	0.31747228	0.05872246	0.007768973	0	0.018506628	0	0.030216598	YAP1_Low
TCGA-69-7760-01	0	0.007004145	0.064507514	0.04292187	0	0.2138233	0	0.050871261	0.033540256	0.00E+00	0	0.05379572	0.06739319	0.00E+00	0	0.15099223	0	0.288936709	0	0.015714888	0	0.010498918	YAP1_Low
TCGA-55-6968-01	0.003462373	0	0.092372751	0.0983223	0	0.0733952	0.02818942	0.044165427	0.016322613	0.00E+00	0	0.056048103	0	2.26E-01	0.133602896	0.1925372	0.008578838	0	0	0.025319599	0	0.001451646	YAP1_Low
TCGA-55-A48Z-01	0.002089745	0.004939114	0.088475642	0.09240672	0	0.07201035	0	0.066075223	0.060180117	0.00E+00	0.010593237	0.01249549	0	2.76E-01	0.05688937	0.22383103	0.00508126	0	0	0.028891102	0	0	YAP1_Low
TCGA-97-A4M7-01	0.018948933	0.003838113	0.042952494	0.08045404	0	0.20630097	0	0.05453423	0.033734112	0.00E+00	0	0.025271024	0.072505879	0.00E+00	0.046675776	0.20121555	0.088393963	0.017728605	0.105701613	0	0	0.001744694	YAP1_Low
TCGA-86-A4P7-01	0.011354835	0.037167317	0.110674914	0.1059173	0	0.12226042	0	0.092542566	0.01408363	0.00E+00	0	0.041983385	0.01368543	1.03E-01	0.016504726	0.28141937	0	0.011851029	0.028450292	0.004551202	0	0.004163068	YAP1_Low
TCGA-62-8397-01	0	0.022653987	0.072701016	0.03605965	0	0.14678177	0	0.006443819	0.00504482	0.00E+00	0	0.028766947	0.147047332	4.70E-02	0	0.24467797	0.085765411	0.003184605	0.153922436	0	0	0	YAP1_Low
TCGA-91-6835-01	0.018433941	0.075175742	0	0.1078819	0	0.17294408	0	0.043519243	0.028192876	2.97E-02	0	0.003625758	0	1.27E-01	0.075509252	0.29639299	0	0	0.021135508	0	0	0	YAP1_Low
TCGA-50-5066-01	0	0.017792217	0.127980376	0.13374183	0	0.07553206	0.065465648	0.035748178	0	7.66E-02	0	0.040356973	0	3.29E-02	0.047839352	0.33556145	0.001880486	0	0	0.007860594	0	0.000732279	YAP1_Low
TCGA-38-4628-01	0.001666075	0.004018757	0	0.02594884	0	0.0607841	0	0.025483712	0.059677618	0.00E+00	0	0	0.057828649	8.71E-02	0	0.56820925	0.043594289	0.001661811	0.041206662	0.016212261	0	0.006572151	YAP1_Low
TCGA-97-8177-01	0.009595788	0.031836171	0.039287323	0.06843998	0	0.10360953	0	0.072932642	0.009739894	0.00E+00	0	0.061313138	0.050312628	4.95E-02	0.005161936	0.30604395	0.075496378	0.049865817	0.025907837	0.038515788	0	0.002429872	YAP1_Low
TCGA-44-2661-01	0.006807293	0.02711708	0.029905048	0.080836	0	0.13877176	0.002231994	0.053219114	0.004671257	4.58E-02	0	0.045841479	0.057248483	0.00E+00	0.055301422	0.32049775	0.055938656	0	0.073912524	0	0	0.001904194	YAP1_Low
TCGA-97-8552-01	0	0.077813308	0.010796661	0.06212505	0	0.1279829	0	0.053132963	0.009342432	0.00E+00	0	0.038394651	0.085780619	7.19E-05	0.021962478	0.36687819	0.052463466	0.017828035	0.074059719	0	0	0.001367602	YAP1_Low
TCGA-49-4490-01	0.060389158	0	0.040767062	0.11651412	0	0.15692892	0	0.059667966	0.080696852	0.00E+00	0	0.034466702	0.010652598	1.37E-01	0.057805077	0.19031231	0	0.011917828	0.043354015	0	0	0	YAP1_Low
TCGA-49-4494-01	0.026473749	0	0	0.06859116	0	0.02987936	0.02148793	0.103494945	0	5.43E-02	0	0.059749469	0.014285106	5.34E-02	0.077009909	0.36240999	0.077673272	0.009216142	0.039651049	0	0	0.002419455	YAP1_Low
TCGA-38-4629-01	0.010015801	0	0.052670621	0.08671793	0	0.05846868	0.037713463	0.017139747	0.003795182	8.44E-05	0	0.031153951	0.019242297	3.65E-02	0.083550214	0.53348979	0.005095426	0	0	0.014574417	0	0.009744317	YAP1_Low
TCGA-55-6980-01	0.00544975	0	0.019847489	0.06138058	0	0.14131954	0	0.067717231	0.101049189	0.00E+00	0	0.06867044	0.091834684	0.00E+00	0.018481581	0.25309845	0.093550203	0.045010525	0.012580638	0.017029729	0	0.002979968	YAP1_Low
TCGA-64-1681-01	0.001853866	0.066697679	0.05144977	0.01623093	0	0.16842602	0	0.039685187	0.027146568	0.00E+00	0	0.020776877	0.035488495	2.78E-02	0.108252271	0.23958716	0.104039971	0.01537057	0.076421391	0	0	0.000726856	YAP1_Low
TCGA-75-7025-01	0	0.084304057	0.049202434	0.0766665	0	0.13646615	0	0.070451963	0.022004715	0.00E+00	0	0.05486149	0.078926606	0.00E+00	0	0.19754309	0.083913945	0.065129451	0.080529601	0	0	0	YAP1_Low

EGFR_Mut_Wt_immune infitration

	B cell naive	B cell memory	B cell plasma	T cell CD8+	T cell CD4 + naive	T cell CD4+ memory resting	T cell CD4+ memory activated	T cell follicular helper	T cell regulator y (Tregs)	T cell gamma delta	NK cell resting	NK cell activated	Monocyte	Macroph age M0	Macrophage M1	Macrophage M2	Myeloid dendritic cell resting	Myeloid dendritic cell activated	Mast cell activated	Mast cell resting	Eosi nop hil	Neutroph il	Groups
TCGA-MP-A4SW-01	1.60E-03	0.049945165	0.126705707	0.0798	0	0.158672961	0.00E+00	0.042886925	6.21E-02	0.00E+00	0.00571515	2.45E-02	0.040846297	4.64E-02	0.017999933	0.26887924	8.44E-03	0.014389048	4.96E-02	0	0	1.52E-03	EGFR_Mut
TCGA-97-8547-01	5.22E-03	0	0	0.0192	0	0.023640397	0.00E+00	0.051480885	7.73E-02	0.00E+00	0	0.00E+00	0.002993427	4.20E-01	0	0.31873406	0.00E+00	0.051859146	2.19E-02	0.007755313	0	0.00E+00	EGFR_Mut
TCGA-71-8520-01	0.00E+00	0.00874063	0.044472288	0.00744	0	0.112805482	0.00E+00	0.047234316	2.79E-02	0.00E+00	0.023302218	1.67E-02	0.013418395	2.43E-01	0.034276828	0.28492971	0.00E+00	0.078741063	5.37E-02	0	0	3.11E-03	EGFR_Mut
TCGA-86-8074-01	1.35E-02	0	0.037293583	0.0651	0	0.085804673	0.00E+00	0.069938662	3.26E-02	0.00E+00	0	6.66E-02	0.02103249	7.15E-02	0.103915655	0.31747228	5.87E-02	0.007768973	0.00E+00	0.018506628	0	3.02E-02	EGFR_Mut
TCGA-05-4402-01	1.82E-02	0.000647203	0.095415049	0.0429	0	0.104503557	0.00E+00	0.030025231	3.06E-02	0.00E+00	0	1.99E-02	0.009169463	4.31E-02	0.074648764	0.41339453	1.06E-02	0.030258705	5.04E-02	0	0	2.62E-02	EGFR_Mut
TCGA-78-7147-01	3.82E-02	0	0	0.0902	0	0.107477094	0.00E+00	0.062308419	7.05E-02	1.02E-02	0	3.91E-02	0.025257831	1.20E-02	0.231063053	0.18922143	1.57E-02	0.014456439	9.42E-02	0	0	0.00E+00	EGFR_Mut
TCGA-86-8075-01	1.20E-02	0.000774008	0.119637311	0.0134	0	0.125977294	0.00E+00	0.039233763	1.49E-02	0.00E+00	0	2.05E-02	0.005268612	9.57E-02	0.061809491	0.37365029	5.53E-03	0.0394747	0.00E+00	0.044183241	0	2.80E-02	EGFR_Mut
TCGA-05-4410-01	0.00E+00	0.041135969	0.216490093	0.157	0	0.1066935	1.87E-03	0.075832675	0.00E+00	0.00E+00	0	2.69E-02	0.019526062	3.04E-02	0.118029434	0.19368813	0.00E+00	0	8.92E-03	0	0	3.44E-03	EGFR_Mut
TCGA-78-7155-01	3.50E-02	0	0.005572699	0.187	0	0.088987157	4.26E-02	0.057815256	0.00E+00	2.92E-02	0	4.18E-02	0.019180809	0.00E+00	0.093766311	0.34798717	2.38E-03	0	0.00E+00	0.048508891	0	0.00E+00	EGFR_Mut
TCGA-69-7760-01	0.00E+00	0.007004145	0.064507514	0.0429	0	0.2138233	0.00E+00	0.050871261	3.35E-02	0.00E+00	0	5.38E-02	0.06739319	0.00E+00	0	0.15099223	0.00E+00	0.288936709	0.00E+00	0.015714888	0	1.05E-02	EGFR_Mut
TCGA-75-6207-01	0.00E+00	0.002676425	0.031616077	0.0643	0	0.129947473	0.00E+00	0.026197595	2.77E-02	0.00E+00	0	5.70E-03	0.058539938	1.94E-01	0.008444073	0.36653297	4.78E-03	0.038521768	2.59E-02	0	0	1.52E-02	EGFR_Mut
TCGA-05-4382-01	0.00E+00	0.008003789	0.11380273	0.0303	0	0.061256194	2.72E-03	0.031539432	2.21E-02	0.00E+00	0	1.68E-02	0	1.44E-01	0.049249653	0.45723941	0.00E+00	0	0.00E+00	0.035179929	0	2.81E-02	EGFR_Mut
TCGA-49-4501-01	7.15E-03	0.061731066	0.088129805	0.0533	0	0.228185775	0.00E+00	0.053162035	1.53E-02	1.16E-02	0	4.20E-02	0.032306686	0.00E+00	0.033521694	0.26100373	1.68E-02	0.039701297	5.43E-02	0	0	1.96E-03	EGFR_Mut
TCGA-55-6968-01	3.46E-03	0	0.092372751	0.0983	0	0.0733952	2.82E-02	0.044165427	1.63E-02	0.00E+00	0	5.60E-02	0	2.26E-01	0.133602896	0.1925372	8.58E-03	0	0.00E+00	0.025319599	0	1.45E-03	EGFR_Mut
TCGA-55-6981-01	5.26E-02	0.014868665	0.126172149	0.0104	0	0.110981896	3.31E-03	0.054143474	9.97E-03	0.00E+00	0.006815593	3.40E-03	0.041411465	7.04E-02	0	0.2402699	8.87E-02	0.060819859	1.00E-01	0	0	5.37E-03	EGFR_Mut
TCGA-55-A48Z-01	2.09E-03	0.004939114	0.088475642	0.0924	0	0.072010349	0.00E+00	0.066075223	6.02E-02	0.00E+00	0.010593237	1.25E-02	0	2.76E-01	0.05688937	0.22383103	5.08E-03	0	0.00E+00	0.028891102	0	0.00E+00	EGFR_Mut
TCGA-97-A4M7-01	1.89E-02	0.003838113	0.042952494	0.0805	0	0.206300967	0.00E+00	0.05453423	3.37E-02	0.00E+00	0	2.53E-02	0.07250588	0.00E+00	0.046675776	0.20121555	8.84E-02	0.017728605	1.06E-01	0	0	1.74E-03	EGFR_Mut
TCGA-67-6217-01	0.00E+00	0.0729291	0.125882948	0.139	0	0.145396063	0.00E+00	0.035915125	3.58E-02	0.00E+00	0	2.97E-02	0.017456268	0.00E+00	0.051291434	0.23024436	6.37E-02	0.014174702	3.59E-02	0	0	2.79E-03	EGFR_Mut
TCGA-L9-A50W-01	6.05E-03	0	0.116172026	0.0372	0	0.203447176	0.00E+00	0.050168319	2.11E-02	0.00E+00	0	5.70E-02	0.018106346	0.00E+00	0.056521195	0.17320137	1.48E-01	0	1.11E-01	0	0	1.54E-03	EGFR_Mut
TCGA-86-A4P7-01	1.14E-02	0.037167317	0.110674914	0.106	0	0.122260415	0.00E+00	0.092542566	1.41E-02	0.00E+00	0	4.20E-02	0.01368543	1.03E-01	0.016504726	0.28141937	0.00E+00	0.011851029	2.85E-02	0.004551202	0	4.16E-03	EGFR_Mut
TCGA-86-8055-01	4.33E-03	0	0.13106745	0.0349	0	0.136953328	0.00E+00	0.011937256	1.96E-02	0.00E+00	0	1.29E-02	0.010830034	1.87E-01	0.077032629	0.29179891	5.92E-03	0.008929395	1.87E-02	0.017603432	0	3.08E-02	EGFR_Mut
TCGA-62-8397-01	0.00E+00	0.022653987	0.072701016	0.0361	0	0.146781773	0.00E+00	0.006443819	5.04E-03	0.00E+00	0	2.88E-02	0.147047332	4.70E-02	0	0.24467797	8.58E-02	0.003184605	1.54E-01	0	0	0.00E+00	EGFR_Mut
TCGA-55-8206-01	0.00E+00	0.011538145	0.030474838	0.0317	0	0.088407289	1.05E-03	0.019351815	1.97E-02	0.00E+00	0	1.65E-02	0.353927513	0.00E+00	0	0.18078978	8.99E-02	0.010488502	1.46E-01	0	0	0.00E+00	EGFR_Mut
TCGA-91-6835-01	1.84E-02	0.075175742	0	0.108	0	0.172944082	0.00E+00	0.043519243	2.82E-02	2.97E-02	0	3.63E-03	0	1.27E-01	0.075509252	0.29639299	0.00E+00	0	2.11E-02	0	0	0.00E+00	EGFR_Mut
TCGA-62-8402-01	2.11E-02	0	0	0.0646	0	0.119573119	2.37E-02	0.034463866	1.49E-02	8.16E-03	0	8.64E-02	0	1.77E-01	0.289410523	0.09573166	1.90E-02	0	3.60E-02	0	0	1.04E-02	EGFR_Mut
TCGA-50-5066-01	0.00E+00	0.017792217	0.127980376	0.134	0	0.075532063	6.55E-02	0.035748178	0.00E+00	7.66E-02	0	4.04E-02	0	3.29E-02	0.047839352	0.33556145	1.88E-03	0	0.00E+00	0.007860594	0	7.32E-04	EGFR_Mut
TCGA-78-7158-01	0.00E+00	0.033101098	0.019896042	0.0287	0	0.235616452	0.00E+00	0.015850248	1.65E-03	0.00E+00	0	3.95E-02	0.055023497	4.91E-03	0.039316161	0.25488134	6.11E-02	0.016446284	1.94E-01	0	0	0.00E+00	EGFR_Mut
TCGA-55-8616-01	6.57E-02	0.086160595	0.125978817	0.115	0	0.108455665	0.00E+00	0.024034678	3.21E-02	3.50E-03	0	2.07E-03	0.024853373	0.00E+00	0.031488252	0.3441266	0.00E+00	0.00372195	3.27E-02	0	0	0.00E+00	EGFR_Mut
TCGA-95-7947-01	4.13E-02	0	0.053745227	0.102	0	0.225485934	0.00E+00	0.078608291	6.14E-03	0.00E+00	0	2.12E-02	0.009903307	8.13E-03	0.122180556	0.2821113	0.00E+00	0.002348572	4.31E-02	0	0	3.95E-03	EGFR_Mut
TCGA-38-4628-01	1.67E-03	0.004018757	0	0.0259	0	0.060784103	0.00E+00	0.025483712	5.97E-02	0.00E+00	0	0.00E+00	0.057828649	8.71E-02	0	0.56820925	4.36E-02	0.001661811	4.12E-02	0.016212261	0	6.57E-03	EGFR_Mut
TCGA-67-3770-01	9.72E-03	0	0.034317111	0.0503	0	0.176925535	0.00E+00	0.029756092	0.00E+00	3.79E-02	0	3.05E-02	0.01997083	0.00E+00	0.028149682	0.38204098	1.63E-02	0.060828606	1.14E-01	0	0	9.15E-03	EGFR_Mut
TCGA-49-6743-01	3.90E-02	0	0.137109022	0.0824	0	0.132578424	0.00E+00	0.033118838	2.08E-02	0.00E+00	0.001797574	3.18E-03	0.005304087	1.41E-01	0.081227698	0.29708433	0.00E+00	0	0.00E+00	0.018536062	0	6.66E-03	EGFR_Mut
TCGA-97-8177-01	9.60E-03	0.031836171	0.039287323	0.0684	0	0.103609528	0.00E+00	0.072932642	9.74E-03	0.00E+00	0	6.13E-02	0.050312628	4.95E-02	0.005161936	0.30604395	7.55E-02	0.049865817	2.59E-02	0.038515788	0	2.43E-02	EGFR_Mut
TCGA-97-8171-01	0.00E+00	0.045874165	0.057119875	0.0155	0	0.045227236	0.00E+00	0.064725156	0.00E+00	0.00E+00	0	2.96E-02	0.014471168	1.80E-01	0.013945923	0.4855779	0.00E+00	0.010838084	3.75E-02	0	0	0.00E+00	EGFR_Mut
TCGA-44-A4SU-01	5.09E-03	0.097991658	0.175835232	0.0411	0	0.107519062	0.00E+00	0.110033749	5.75E-02	0.00E+00	0	3.10E-02	0	8.15E-02	0.080568557	0.13379632	3.13E-02	0	4.67E-02	0	0	0.00E+00	EGFR_Mut
TCGA-55-A57B-01	1.55E-02	0	0	0.0476	0	0.194953556	0.00E+00	0.013096634	2.72E-02	0.00E+00	0	5.27E-02	0.074354687	0.00E+00	0.011380134	0.30291077	1.35E-01	0.041707588	8.16E-02	0	0	2.38E-03	EGFR_Mut
TCGA-44-5645-01	2.27E-03	0.0579762	0.028934426	0.0536	0	0.275605717	0.00E+00	0.029931654	5.40E-02	0.00E+00	0	9.59E-03	0.054151611	0.00E+00	0.02356306	0.22622218	1.04E-01	0.026258118	5.34E-02	0	0	0.00E+00	EGFR_Mut
TCGA-50-6591-01	1.78E-03	0	0.049880695	0.0257	0	0.152938501	0.00E+00	0.022763191	2.44E-02	0.00E+00	0.044396737	0.00E+00	0	5.60E-01	0.010963085	0.02657473	1.83E-02	0	0.00E+00	0.062312738	0	0.00E+00	EGFR_Mut
TCGA-95-7039-01	1.44E-02	0	0.022563721	0.122	0	0.092846904	0.00E+00	0.096418789	5.96E-03	2.09E-02	0	2.16E-02	0	1.12E-01	0.140123978	0.31988409	9.48E-03	0	1.45E-02	0.007848405	0	0.00E+00	EGFR_Mut
TCGA-50-6595-01	7.52E-03	0	0.016159542	0.0768	0	0.086086893	6.06E-04	0.0275228	1.09E-02	0.00E+00	0.035472615	1.83E-03	0	2.11E-01	0.160341081	0.36313752	0.00E+00	0	0.00E+00	0.002855288	0	0.00E+00	EGFR_Mut
TCGA-55-8506-01	0.00E+00	0.005771964	0.061485498	0.0608	0	0.032805976	0.00E+00	0.063302544	7.62E-04	5.32E-03	0	2.94E-02	0.03470616	3.05E-01	0.051098107	0.31084019	1.03E-03	0	0.00E+00	0.03739933	0	0.00E+00	EGFR_Mut
TCGA-44-2661-01	6.81E-03	0.02711708	0.029905048	0.0808	0	0.138771756	2.23E-03	0.053219114	4.67E-03	4.58E-02	0	4.58E-02	0.057248483	0.00E+00	0.055301422	0.32049775	5.59E-02	0	7.39E-02	0	0	1.90E-03	EGFR_Mut
TCGA-55-7570-01	0.00E+00	0	0.243582735	0.155	0	0	1.27E-02	0.154224812	0.00E+00	0.00E+00	0	4.03E-02	0.025479096	1.09E-01	0.023130978	0.20253061	2.88E-04	0	0.00E+00	0.033438236	0	0.00E+00	EGFR_Mut
TCGA-86-8280-01	0.00E+00	0.037081096	0.061268562	0.0576	0	0.174756885	0.00E+00	0.082583175	3.34E-02	0.00E+00	0	2.64E-02	0.028182184	4.54E-02	0.11053867	0.23510503	3.93E-02	0.011930695	5.64E-02	0	0	0.00E+00	EGFR_Mut
TCGA-97-8552-01	0.00E+00	0.077813308	0.010796661	0.0621	0	0.127982895	0.00E+00	0.053132963	9.34E-03	0.00E+00	0	3.84E-02	0.085780619	7.19E-05	0.021962478	0.36687819	5.25E-02	0.017828035	7.41E-02	0	0	1.37E-03	EGFR_Mut
TCGA-49-4490-01	6.04E-02	0	0.040767062	0.117	0	0.156928925	0.00E+00	0.059667966	8.07E-02	0.00E+00	0	3.45E-02	0.010652598	1.37E-01	0.057805077	0.19031231	0.00E+00	0.011917828	4.34E-02	0	0	0.00E+00	EGFR_Mut
TCGA-97-A4M1-01	1.01E-02	0.003335997	0	0.072	0	0.105849567	0.00E+00	0.03244254	3.57E-02	0.00E+00	0.03597631	2.02E-02	0.124676259	3.60E-02	0.022949079	0.30475697	1.28E-02	0.0102335	1.73E-01	0	0	0.00E+00	EGFR_Mut
TCGA-49-4494-01	2.65E-02	0	0	0.0686	0	0.029879362	2.15E-02	0.103494945	0.00E+00	5.43E-02	0	5.97E-02	0.014285106	5.34E-02	0.077009909	0.36240999	7.77E-02	0.009216142	3.97E-02	0	0	2.42E-03	EGFR_Mut
TCGA-67-3771-01	9.83E-03	0	0.116435737	0.0947	0	0.132509143	1.87E-02	0.035997183	7.01E-03	0.00E+00	0	1.11E-02	0.032962456	8.02E-02	0.105731989	0.33781651	0.00E+00	0	1.51E-02	0	0	1.92E-03	EGFR_Mut
TCGA-38-4629-01	1.00E-02	0	0.052670621	0.0867	0	0.058468682	3.77E-02	0.017139747	3.80E-03	8.44E-05	0	3.12E-02	0.019242297	3.65E-02	0.083550214	0.53348979	5.10E-03	0	0.00E+00	0.014574417	0	9.74E-03	EGFR_Mut
TCGA-55-6980-01	5.45E-03	0	0.019847489	0.0614	0	0.141319545	0.00E+00	0.067717231	1.01E-01	0.00E+00	0	6.87E-02	0.091834684	0.00E+00	0.018481581	0.25309845	9.36E-02	0.045010525	1.26E-02	0.017029729	0	2.98E-03	EGFR_Mut
TCGA-69-7765-01	0.00E+00	0.046679115	0.162334221	0.0664	0	0.134550456	4.75E-02	0.050629679	3.53E-03	0.00E+00	0.015800621	0.00E+00	0.004267339	7.24E-02	0.062590031	0.1927835	1.17E-01	0.010983301	1.16E-02	0.001184878	0	0.00E+00	EGFR_Mut
TCGA-55-8096-01	0.00E+00	0.020210032	0.133592119	0.0392	0	0.074467252	0.00E+00	0	1.87E-02	0.00E+00	0	7.50E-03	0.026210341	2.69E-01	0.026828895	0.34924826	0.00E+00	0	3.52E-02	0	0	0.00E+00	EGFR_Mut
TCGA-38-6178-01	1.32E-02	0.007275998	0.190406751	0.036	0	0.17411002	2.08E-03	0.001846164	0.00E+00	0.00E+00	0.018625801	0.00E+00	0.007168405	3.17E-03	0.051026567	0.35434224	0.00E+00	0.053205927	8.76E-02	0	0	0.00E+00	EGFR_Mut
TCGA-97-A4M6-01	1.28E-02	0.046822673	0	0.0588	0	0.208885759	0.00E+00	0.084256649	5.68E-02	0.00E+00	0	3.23E-02	0.028628885	7.16E-02	0.104929998	0.21824178	2.09E-02	0	5.26E-02	0	0	2.50E-03	EGFR_Mut
TCGA-64-1681-01	1.85E-03	0.066697679	0.05144977	0.0162	0	0.168426018	0.00E+00	0.039685187	2.71E-02	0.00E+00	0	2.08E-02	0.035488495	2.78E-02	0.108252272	0.23958716	1.04E-01	0.015370571	7.64E-02	0	0	7.27E-04	EGFR_Mut
TCGA-62-8394-01	3.69E-02	0	0.060261548	0.0406	0	0.232910368	0.00E+00	0.042000898	2.58E-02	0.00E+00	0.00666342	6.96E-03	0.020295239	1.80E-01	0.075500798	0.20304653	0.00E+00	0.059865055	8.94E-03	0	0	0.00E+00	EGFR_Mut
TCGA-93-A4JP-01	7.83E-03	0	0	0.03	0	0.08764099	0.00E+00	0.043672024	0.00E+00	0.00E+00	0.005084495	1.97E-02	0.044133153	9.67E-02	0.055882193	0.39263706	0.00E+00	0.093536173	1.18E-01	0	0	5.33E-03	EGFR_Mut
TCGA-75-7025-01	0.00E+00	0.084304057	0.049202434	0.0767	0	0.136466151	0.00E+00	0.070451963	2.20E-02	0.00E+00	0	5.49E-02	0.078926606	0.00E+00	0	0.19754309	8.39E-02	0.065129451	8.05E-02	0	0	0.00E+00	EGFR_Mut
TCGA-J2-8192-01	0.00E+00	0.014320324	0.027230846	0.018	0	0.237090779	0.00E+00	0.005316681	0.00E+00	0.00E+00	0	1.74E-02	0.029443199	1.02E-01	0.015345291	0.36146388	8.27E-02	0.026984513	5.21E-02	0	0	1.01E-02	EGFR_Mut
TCGA-50-5944-01	3.62E-02	0	0.023849213	0.0331	0	0.126695544	0.00E+00	0.017346312	1.14E-03	0.00E+00	0	2.58E-02	0.080551253	5.60E-02	0.028052936	0.39365055	3.96E-03	0	1.74E-01	0	0	0.00E+00	EGFR_Mut
TCGA-MP-A4T9-01	1.04E-02	0.025611603	0	0.0952	0	0.11610841	0.00E+00	0.060593792	1.68E-02	0.00E+00	0	4.29E-02	0.054669168	4.80E-02	0.03107934	0.34922277	1.43E-02	0.022778807	1.07E-01	0	0	5.89E-03	EGFR_Mut
TCGA-38-4627-01	2.64E-03	0	0.038926234	0.0293	0	0.198523212	0.00E+00	0	0.00E+00	0.00E+00	0	2.08E-02	0.03877708	0.00E+00	0.035133913	0.55904173	4.37E-03	0	6.66E-02	0	0	5.93E-03	EGFR_Mut

TCGA-67-3772-01	3.17E-02	0	0.088477074	0.0511	0	0.212631691	0.00E+00	0.031924054	5.37E-03	0.00E+00	0	0.00E+00	0.077971551	0.00E+00	0.024525077	0.30375953	8.49E-02	0.029408194	5.34E-02	0	0	4.81E-03	EGFR_Mut
TCGA-86-8668-01	6.30E-03	0.040273063	0.091847704	0.0642	0	0.152788759	0.00E+00	0.061442672	3.24E-02	0.00E+00	0	4.65E-02	0.023397806	1.92E-02	0.05942804	0.28018358	2.46E-02	0	9.74E-02	0	0	0.00E+00	EGFR_Mut
TCGA-97-7938-01	9.51E-02	0	0.051507198	0.0955	0	0.105443011	0.00E+00	0.052222053	0.00E+00	8.99E-03	0	5.46E-02	0.000543584	7.98E-02	0.035998369	0.29892636	2.73E-02	0.005093644	6.45E-02	0.007121881	0	1.73E-02	EGFR_Wt
TCGA-MP-A4TJ-01	7.91E-03	0.0659602	0.08304659	0.122	0	0.155960287	0.00E+00	0.11455937	2.63E-02	9.58E-03	0	4.99E-02	0.005409855	7.69E-02	0.108073266	0.1575968	0.00E+00	0	0.00E+00	0.013289154	0	4.04E-03	EGFR_Wt
TCGA-97-7546-01	3.54E-02	0.013612921	0.076782724	0.0868	0	0.279974541	0.00E+00	0.026351703	9.97E-03	0.00E+00	0	9.23E-03	0.054955715	5.67E-03	0.043322734	0.21551688	2.39E-02	0.013754641	6.18E-02	0.038899071	0	4.16E-03	EGFR_Wt
TCGA-44-7670-01	6.37E-03	0.030259543	0.069534238	0.11	0	0.018018382	1.26E-02	0.123374665	0.00E+00	0.00E+00	0	9.21E-02	0.015610665	2.33E-01	0.087566811	0.16309345	0.00E+00	0	0.00E+00	0.036239789	0	2.27E-03	EGFR_Wt
TCGA-62-A46Y-01	0.00E+00	0.023375532	0.027665926	0.0949	0	0.095958174	0.00E+00	0.070943085	2.78E-03	0.00E+00	0	4.55E-02	0.0630555	2.66E-02	0.046107025	0.36292864	6.40E-02	0.026739001	4.62E-02	0	0	3.15E-03	EGFR_Wt
TCGA-78-8640-01	7.86E-03	0.005030553	0.037581849	0.146	0	0.096375988	1.25E-02	0.084036086	1.01E-02	0.00E+00	0	7.08E-02	0.025369828	1.88E-01	0.066427751	0.23329887	3.21E-03	0	5.82E-03	0.005554115	0	2.79E-03	EGFR_Wt
TCGA-J2-A4AG-01	3.01E-02	0.091835028	0.050367939	0.0696	0	0.216189126	0.00E+00	0.040188788	3.90E-02	0.00E+00	0	2.87E-02	0.043449155	0.00E+00	0.021220227	0.24080723	5.19E-02	0.05475786	4.65E-04	0.021376507	0	0.00E+00	EGFR_Wt
TCGA-95-7043-01	0.00E+00	0.027956989	0.14788676	0.146	0	0.061057306	0.00E+00	0.112306164	4.62E-02	0.00E+00	0	4.60E-02	0.015084112	0.00E+00	0.035951617	0.31755691	7.14E-03	0.010388927	2.69E-02	0	0	0.00E+00	EGFR_Wt
TCGA-55-A48X-01	4.18E-02	0.201194682	0.044869876	0.07	0	0.153604093	0.00E+00	0.050790342	6.01E-02	2.18E-03	0	4.80E-02	0.004138797	3.49E-02	0.043083116	0.17945006	7.11E-03	0	5.87E-02	0	0	0.00E+00	EGFR_Wt
TCGA-55-8620-01	1.90E-02	0.017145889	0.095843976	0.146	0	0.090551346	2.04E-02	0.055306847	3.53E-02	0.00E+00	0	4.75E-02	0.010460699	1.47E-01	0.124788428	0.14872536	6.82E-03	0	3.57E-02	0	0	0.00E+00	EGFR_Wt
TCGA-55-8092-01	0.00E+00	0.036738627	0.265094485	0.239	0	0.051347117	8.94E-03	0.05615366	1.36E-02	0.00E+00	0	6.64E-02	0.010166068	0.00E+00	0.10821256	0.14459656	0.00E+00	0	0.00E+00	0	0	0.00E+00	EGFR_Wt
TCGA-78-7163-01	1.67E-03	0.018489305	0.005364871	0.27	0	0.151630086	0.00E+00	0.071818179	6.20E-02	0.00E+00	0	4.86E-02	0.014996484	2.90E-02	0.02620097	0.14974582	4.05E-03	0.015811339	1.31E-01	0	0	0.00E+00	EGFR_Wt
TCGA-86-8279-01	0.00E+00	0.014995074	0.061382815	0.0303	0	0.023283564	0.00E+00	0.042537307	1.59E-02	0.00E+00	0	2.95E-02	0.037554801	2.52E-01	0.030758266	0.25499076	1.60E-01	0.00481266	0.00E+00	0.042451878	0	0.00E+00	EGFR_Wt
TCGA-05-5425-01	1.96E-02	0	0.061383189	0.146	0	0.107905416	3.76E-03	0.05862821	1.71E-02	1.46E-02	0	3.98E-02	0.003825562	3.91E-02	0.159819293	0.29820031	0.00E+00	0.002556816	3.27E-03	0	0	2.42E-02	EGFR_Wt
TCGA-05-4390-01	0.00E+00	0.027672657	0.136867389	0.0452	0	0.096340826	0.00E+00	0.017122174	2.39E-02	0.00E+00	0.013686456	0.00E+00	0.009109678	3.01E-01	0.047371639	0.25703465	0.00E+00	0	0.00E+00	0.022632111	0	2.32E-03	EGFR_Wt
TCGA-78-7152-01	0.00E+00	0.136454838	0.052547268	0.134	0	0.215180982	0.00E+00	0.030117102	5.27E-02	0.00E+00	0	6.10E-02	0.002081179	4.42E-02	0.051723333	0.17826086	9.85E-03	0	2.79E-02	0	0	3.69E-03	EGFR_Wt
TCGA-05-4427-01	2.02E-02	0	0.041531971	0.119	0	0.100493382	4.25E-03	0.044372832	0.00E+00	0.00E+00	0	3.37E-02	0.070546434	2.77E-02	0.068750359	0.42694311	1.55E-02	0.008581319	0.00E+00	0.016313424	0	2.27E-03	EGFR_Wt
TCGA-55-8508-01	0.00E+00	0.052994003	0.316872916	0.0345	0	0.040720858	1.65E-02	0.030965003	3.75E-02	0.00E+00	0.034880408	0.00E+00	0	2.74E-01	0.01206429	0.0647309	0.00E+00	0	0.00E+00	0.056630603	0	2.76E-02	EGFR_Wt
TCGA-05-4426-01	2.12E-02	0	0.000959892	0.0685	0	0.194841589	0.00E+00	0.006743863	5.60E-02	0.00E+00	0	2.07E-02	0.092091535	1.38E-01	0.024241854	0.30726272	3.24E-02	0	2.96E-02	0.005750426	0	1.80E-03	EGFR_Wt
TCGA-44-3918-01	0.00E+00	0.005430585	0.10895281	0.131	0	0.118480029	6.03E-03	0.069889581	0.00E+00	0.00E+00	0	5.48E-02	0.003052907	3.16E-03	0.129949639	0.33688077	0.00E+00	0	0.00E+00	0.029716338	0	3.00E-03	EGFR_Wt
TCGA-97-8172-01	3.21E-02	0.127168066	0.021044539	0.124	0	0.184350656	0.00E+00	0.033185345	4.51E-02	0.00E+00	0	2.28E-02	0.008032747	0.00E+00	0.028492768	0.24944595	5.82E-03	0.004174926	1.11E-01	0	0	3.22E-03	EGFR_Wt
TCGA-86-8359-01	7.14E-03	0	0.092305221	0.266	0	0.074546344	1.54E-02	0.136761875	7.29E-02	0.00E+00	0	9.06E-02	0.029135332	7.23E-02	0.02782224	0.02362997	0.00E+00	0	0.00E+00	0.062066067	0	2.94E-02	EGFR_Wt
TCGA-49-AAQV-01	0.00E+00	0.058764468	0.072528466	0.0673	0	0.12927931	0.00E+00	0.071585183	7.98E-02	0.00E+00	0	4.38E-02	0	2.45E-01	0.052280144	0.15564079	6.08E-03	0	1.78E-02	0	0	0.00E+00	EGFR_Wt
TCGA-64-1677-01	2.54E-02	0.03867948	0.137358897	0.109	0	0.136259556	1.56E-02	0.09154555	3.09E-02	3.80E-02	0	3.24E-02	0	4.53E-02	0.166994502	0.10511804	0.00E+00	0	0.00E+00	0.026959041	0	1.69E-04	EGFR_Wt
TCGA-91-8497-01	1.64E-02	0.067828163	0.194667956	0.0938	0	0.146038098	0.00E+00	0.046898993	4.14E-02	0.00E+00	0	3.72E-02	0.023340777	3.53E-02	0.008315668	0.16099781	5.86E-02	0.002531593	6.64E-02	0	0	1.07E-04	EGFR_Wt
TCGA-50-5946-01	3.51E-02	0	0.094420545	0.149	0	0.052442174	0.00E+00	0.130356541	1.19E-02	0.00E+00	0	3.79E-02	0.044183345	0.00E+00	0.060309091	0.32686876	4.25E-03	0.014434485	3.17E-02	0	0	7.07E-03	EGFR_Wt
TCGA-49-AAR9-01	0.00E+00	0	0.035683033	0.0889	0	0	0.00E+00	0.116874496	1.79E-02	0.00E+00	0	1.03E-01	0	3.62E-01	0.080338763	0.13540047	0.00E+00	0.045654297	0.00E+00	0.014041244	0	0.00E+00	EGFR_Wt
TCGA-MP-A4TF-01	0.00E+00	0.007675149	0.269854363	0.0862	0	0.129173658	1.16E-02	0.036341375	0.00E+00	0.00E+00	0.015466079	4.08E-02	0.000893946	1.13E-01	0.061600105	0.09042489	0.00E+00	0.072549858	0.00E+00	0.04795529	0	1.66E-02	EGFR_Wt
TCGA-49-AAR4-01	8.30E-03	0.008644917	0.071428552	0.379	0	0	4.14E-02	0.085422991	1.32E-02	1.17E-01	0	1.06E-02	0	5.84E-02	0.056493413	0.12996684	2.89E-03	0	1.12E-05	0.015255978	0	1.33E-03	EGFR_Wt
TCGA-86-7713-01	1.35E-02	0	0.187521317	0.0279	0	0.122878589	0.00E+00	0.102099527	3.25E-03	0.00E+00	0	3.46E-02	0.030882874	8.09E-02	0.066155693	0.23421148	0.00E+00	0.066270966	1.14E-02	0	0	1.83E-02	EGFR_Wt
TCGA-78-8660-01	1.60E-02	0.005016708	0	0.125	0	0.099632886	3.76E-03	0.141838236	2.10E-02	0.00E+00	0	7.79E-02	0.002151627	1.11E-01	0.10010541	0.26271216	4.32E-03	0	0.00E+00	0.027091352	0	2.34E-03	EGFR_Wt
TCGA-83-5908-01	9.52E-03	0	0	0.266	0	0.048803819	1.25E-01	0.100424476	0.00E+00	0.00E+00	0	9.49E-02	0.007535866	0.00E+00	0.127239854	0.16018536	2.47E-02	0.010450578	0.00E+00	0.016402752	0	8.27E-03	EGFR_Wt
TCGA-55-6642-01	0.00E+00	0.008520018	0.354315952	0.11	0	0.107376641	2.41E-02	0.013418395	3.98E-03	0.00E+00	0.004238471	7.88E-04	0	8.29E-02	0.036284183	0.23156592	0.00E+00	0	1.24E-02	0.005519957	0	4.86E-03	EGFR_Wt
TCGA-55-6543-01	5.51E-03	0	0.06191523	0.0303	0	0.125251165	0.00E+00	0.007791626	8.07E-03	0.00E+00	0	2.64E-02	0.027899886	1.29E-01	0.038883086	0.45613357	2.02E-03	0	0.00E+00	0.043683453	0	3.71E-02	EGFR_Wt
TCGA-05-4397-01	9.73E-03	0	0.015245725	0.0389	0	0.008749548	0.00E+00	0.03575128	1.18E-02	0.00E+00	0.00533116	1.46E-02	0.005298567	2.40E-01	0.106146044	0.36517599	0.00E+00	0.090407621	3.71E-02	0	0	1.54E-02	EGFR_Wt
TCGA-55-8621-01	0.00E+00	0.010550012	0.107783423	0.0546	0	0.134481073	0.00E+00	0.032130255	1.10E-05	0.00E+00	0.033098137	1.44E-02	0.039919163	1.46E-01	0.086921804	0.25849718	0.00E+00	0	7.75E-02	0	0	3.64E-03	EGFR_Wt
TCGA-55-6978-01	1.19E-04	0	0.005535497	0.228	0	0.022120545	6.77E-02	0.068247785	2.95E-03	1.63E-02	0	5.54E-02	0.000683644	4.20E-02	0.089782981	0.33653798	4.06E-02	0	0.00E+00	0.024572216	0	0.00E+00	EGFR_Wt
TCGA-44-2659-01	2.10E-03	0	0.116363911	0.175	0	0.18654593	0.00E+00	0.038458097	4.15E-02	1.75E-02	0	1.74E-02	0.007471313	3.87E-03	0.087059588	0.24186169	0.00E+00	0.006079643	5.43E-02	0	0	4.59E-03	EGFR_Wt
TCGA-44-5643-01	5.85E-02	0	0.032530272	0.136	0	0.10725397	6.39E-02	0.090375332	1.61E-02	0.00E+00	0.009582373	2.42E-02	0.030002586	5.65E-02	0.080499741	0.2460797	2.95E-02	0.012126095	6.94E-03	0	0	0.00E+00	EGFR_Wt
TCGA-78-7156-01	1.75E-03	0	0.174467938	0.142	0	0.116133932	0.00E+00	0.061280333	1.00E-02	0.00E+00	0.000659335	0.00E+00	0.048221842	0.00E+00	0.126248781	0.16998055	0.00E+00	0.008058887	1.42E-01	0	0	0.00E+00	EGFR_Wt
TCGA-50-5942-01	2.27E-02	0	0.190365345	0.0893	0	0.191207436	0.00E+00	0.04033324	8.83E-03	0.00E+00	0	1.90E-02	0.040934002	0.00E+00	0.032502311	0.18410065	2.29E-02	0	1.58E-01	0	0	0.00E+00	EGFR_Wt
TCGA-50-6590-01	0.00E+00	0.022939582	0.123555869	0.102	0	0.076044396	5.65E-03	0.044297893	1.73E-02	0.00E+00	0	7.42E-02	0	1.14E-01	0.129530886	0.25949862	0.00E+00	0	0.00E+00	0.027889564	0	3.15E-03	EGFR_Wt
TCGA-55-A48Y-01	5.04E-03	0.003087169	0.18781076	0.0578	0	0.163301686	1.80E-03	0.030879462	2.95E-02	0.00E+00	0	2.96E-02	0	2.20E-01	0.06724829	0.1770356	0.00E+00	0	3.74E-04	0.013151805	0	1.30E-02	EGFR_Wt
TCGA-75-5147-01	1.75E-02	0.003192286	0.131987424	0.0592	0	0.145182241	2.04E-03	0.022553855	4.31E-02	1.37E-02	0	2.32E-02	0.052556988	0.00E+00	0.040072908	0.31693907	2.65E-02	0.013402026	3.71E-02	0.026057646	0	2.57E-02	EGFR_Wt
TCGA-44-3396-01	8.67E-04	0.009196548	0.152816701	0.0863	0	0.127309313	0.00E+00	0.026172717	8.08E-03	0.00E+00	0	1.97E-02	0.022751286	9.98E-02	0.093024954	0.27523696	1.73E-03	0	0.00E+00	0.049034979	0	2.80E-02	EGFR_Wt
TCGA-93-A4JO-01	9.59E-03	0.048587056	0.016938452	0.105	0	0.139927415	0.00E+00	0.080323554	4.21E-02	0.00E+00	0	2.37E-02	0.023921432	1.13E-01	0.081216525	0.28025671	7.84E-03	0	2.70E-02	0	0	0.00E+00	EGFR_Wt
TCGA-62-8395-01	1.88E-02	0.004291973	0.102052781	0.0632	0	0.135542187	0.00E+00	0.069340811	2.82E-02	0.00E+00	0	6.46E-02	0.031524261	0.00E+00	0.025812769	0.37268798	5.74E-02	0	7.92E-03	0.016757828	0	1.81E-03	EGFR_Wt
TCGA-44-2662-01	0.00E+00	0.025289558	0.009983793	0.0136	0	0.096849491	2.53E-02	0.007102141	0.00E+00	0.00E+00	0.028049149	0.00E+00	0.200390806	1.74E-01	0.037832471	0.33708002	0.00E+00	0	1.84E-02	0	0	2.62E-02	EGFR_Wt
TCGA-50-5931-01	6.57E-02	0	0.088947731	0.0522	0	0	0.00E+00	0.183503427	0.00E+00	0.00E+00	0	2.68E-02	0	2.99E-01	0.065791065	0.18651548	0.00E+00	0.01084794	0.00E+00	0.021145131	0	0.00E+00	EGFR_Wt
TCGA-44-A47G-01	1.82E-03	0.051486852	0.089788065	0.11	0	0.171994301	0.00E+00	0.035774714	4.21E-02	8.63E-03	0	1.54E-02	0.028994594	1.20E-02	0.077244775	0.27297977	2.30E-02	0.000476825	5.40E-02	0	0	4.12E-03	EGFR_Wt
TCGA-55-8301-01	0.00E+00	0.048025375	0.044756334	0.0882	0	0.118865348	2.39E-02	0.042344316	5.17E-02	0.00E+00	0.002544338	3.31E-02	0.003253807	1.65E-01	0.206053934	0.11521592	1.72E-02	0	4.01E-02	0	0	0.00E+00	EGFR_Wt
TCGA-86-8669-01	4.19E-03	0.010380306	0.091351414	0.0828	0	0.180664116	0.00E+00	0.040463407	5.44E-02	0.00E+00	0	6.45E-02	0.095659431	3.93E-02	0.028198809	0.17372319	1.01E-01	0.015803305	0.00E+00	0.017748412	0	0.00E+00	EGFR_Wt
TCGA-55-7724-01	4.26E-02	0	0.075824232	0.0571	0	0.063751309	1.98E-03	0.023335777	0.00E+00	7.06E-02	0	4.93E-03	0	3.94E-02	0.103097186	0.39844233	1.64E-02	0.005385852	5.74E-02	0	0	3.97E-02	EGFR_Wt
TCGA-MN-A4N5-01	0.00E+00	0.040953132	0.027215919	0.242	0	0.060074238	0.00E+00	0.126991214	3.37E-03	0.00E+00	0	1.23E-01	0.024207062	5.80E-02	0.123648804	0.13784121	1.75E-03	0	3.02E-02	0	0	7.00E-04	EGFR_Wt
TCGA-50-5072-01	1.93E-02	0	0.093523092	0.0408	0	0.16054624	0.00E+00	0.039725875	4.23E-02	0.00E+00	0.015718963	6.26E-03	0.025960502	1.23E-01	0.062308697	0.32521241	0.00E+00	0.019328174	1.29E-02	0	0	1.33E-02	EGFR_Wt
TCGA-50-8460-01	0.00E+00	0.04573729	0.11796831	0.0843	0	0.098940514	0.00E+00	0.059384773	4.65E-02	0.00E+00	0	4.88E-02	0.117298052	0.00E+00	0	0.1433774	7.53E-02	0.047983208	1.14E-01	0	0	0.00E+00	EGFR_Wt
TCGA-05-4405-01	0.00E+00	0.044703659	0.141341194	0.0696	0	0.074077901	0.00E+00	0.029466443	1.87E-02	0.00E+00	0	9.64E-04	0	1.64E-01	0.018786516	0.35255209	1.20E-02	0	7.37E-02	0	0	0.00E+00	EGFR_Wt
TCGA-97-7941-01	0.00E+00	0.081669368	0	0.0884	0	0.084553952	0.00E+00	0.061761863	6.10E-02	0.00E+00	0	3.82E-02	0.025037557	4.28E-02	0.038653568	0.36815959	1.76E-02	0	9.22E-02	0	0	0.00E+00	EGFR_Wt
TCGA-38-4625-01	1.10E-02	0.009232401	0.050751765	0.0917	0	0.047901152	2.53E-02	0.045486379	0.00E+00	9.66E-03	0	4.56E-02	0.018669762	8.78E-02	0.08759571	0.34693262	0.00E+00	0.055481362	0.00E+00	0.057173118	0	6.83E-03	EGFR_Wt
TCGA-78-7160-01	2.62E-02	0	0.025091851	0.143	0	0.149136816	0.00E+00	0.035855772	1.46E-02	0.00E+00	0	4.87E-02	0.017036133	4.32E-02	0.092210552	0.36688093	3.86E-03	0.023561069	4.33E-03	0	0	6.20E-03	EGFR_Wt
TCGA-55-1596-01	6.23E-02	0	0.257740633	0.0422	0	0.013652233	0.00E+00	0.06509237	5.68E-02	0.00E+00	0.023732141	0.00E+00	0	1.18E-01	0.090271606	0.17754125	0.00E+00	0.07945065	0.00E+00	0.013354638	0	0.00E+00	EGFR_Wt
TCGA-44-7669-01	3.71E-02	0	0.043531909	0.0658	0	0.146070577	1.51E-02	0.045100517	0.00E+00	0.00E+00	0	2.38E-02	0.016008301	6.50E-02	0.104909249	0.38046538	5.31E-03	0	4.58E-02	0	0	6.07E-03	EGFR_Wt
TCGA-55-6983-01	3.65E-02	0.120343634	0.061346565	0.0932	0	0.23719426	0.00E+00	0.061237599	6.73E-03	0.00E+00	0	3.93E-02	0	7.34E-02	0.019851833	0.20258325	1.06E-02	0.009356119	2.49E-02	0	0	3.51E-03	EGFR_Wt
TCGA-99-AA5R-01	0.00E+00	0.12463598	0.042177407	0.151	0	0.151143447	0.00E+00	0.136319129	1.56E-03	0.00E+00	0	7.40E-02	0.012814651	5.86E-02	0.045543023	0.16087016	5.61E-03	0	9.12E-03	0.02707469	0	0.00E+00	EGFR_Wt
TCGA-67-6216-01	0.00E+00	0.09328775	0.028516063	0.0791	0	0.145318862	0.00E+00	0.02157406	3.94E-02	0.00E+00	0	3.97E-02	0.070892943	3.43E-02	0.012185533	0.24194847	6.90E-02	0.030847928	9.26E-02	0	0	1.34E-03	EGFR_Wt
TCGA-55-6971-01	0.00E+00	0.043456845	0.10659784	0.145	0	0.14752956	0.00E+00	0.073042077	3.18E-02	0.00E+00	0	6.58E-02	0.034547609	6.54E-02	0.082728005	0.16854506	0.00E+00	0	2.86E-02	0	0	7.01E-03	EGFR_Wt

TCGA-L9-A743-01	0.00E+00	0.098089749	0.043531414	0.123	0	0.135258464	4.85E-02	0.052396969	2.68E-02	0.00E+00	0	2.49E-02	0.041620365	1.59E-02	0.044856253	0.22608906	4.09E-02	0.05046488	7.16E-03	0.016224866	0	4.21E-03	EGFR_Wt
TCGA-86-8076-01	4.70E-03	0.028039059	0.113675988	0.189	0	0.124997043	0.00E+00	0.08343211	7.37E-02	0.00E+00	0	4.65E-02	0.009934038	9.92E-02	0.043171721	0.1487888	1.25E-02	0	1.39E-02	0	0	8.12E-03	EGFR_Wt
TCGA-91-6849-01	2.68E-02	0.031656807	0.135196901	0.0415	0	0.041240135	0.00E+00	0.089537224	1.80E-02	0.00E+00	0.033410271	6.11E-03	0	3.50E-01	0.004476711	0.18716221	0.00E+00	0.025606512	0.00E+00	0.005729845	0	3.67E-03	EGFR_Wt
TCGA-55-8204-01	0.00E+00	0.004110303	0.283195431	0.0773	0	0.082602633	0.00E+00	0.02427324	1.28E-03	0.00E+00	0	3.55E-02	0.014277478	5.98E-04	0.140032973	0.20567652	1.67E-02	0	7.51E-02	0	0	3.93E-02	EGFR_Wt
TCGA-86-A4JF-01	6.72E-03	0	0.072967808	0.106	0	0.088820108	1.35E-02	0.12676222	0.00E+00	0.00E+00	0.017396974	6.87E-02	0.032790561	2.98E-02	0.110384624	0.25950008	0.00E+00	0.016531714	4.42E-02	0	0	5.87E-03	EGFR_Wt
TCGA-91-6847-01	6.43E-02	0	0.000553438	0.21	0	0.1306291	0.00E+00	0.036129731	2.73E-03	0.00E+00	0.100403642	9.81E-02	0.019348306	1.15E-01	0.039346392	0.08466253	8.27E-02	0	1.66E-02	0	0	0.00E+00	EGFR_Wt
TCGA-86-7955-01	2.44E-02	0.02574737	0.072734788	0.126	0	0.129934816	0.00E+00	0.138383125	5.95E-02	0.00E+00	0	9.16E-02	0.011638994	4.90E-02	0.065624624	0.11312964	0.00E+00	0.05641929	3.60E-02	0	0	0.00E+00	EGFR_Wt
TCGA-55-7911-01	1.95E-02	0	0.03421589	0.156	0	0.051492171	1.16E-01	0.129531602	1.81E-04	0.00E+00	0	3.19E-02	0.027571575	1.43E-02	0.068336998	0.24378241	0.00E+00	0.101343983	0.00E+00	0.005894392	0	5.14E-05	EGFR_Wt
TCGA-86-7953-01	8.84E-03	0.015566159	0	0.0622	0	0.214416631	0.00E+00	0.050301604	4.76E-02	0.00E+00	0	4.60E-02	0.088321749	9.86E-02	0.031211951	0.28197672	0.00E+00	0.007677417	0.00E+00	0.041951521	0	5.36E-03	EGFR_Wt
TCGA-55-8207-01	0.00E+00	0.011979033	0.010711359	0.0615	0	0.153291239	0.00E+00	0.008005128	3.89E-03	0.00E+00	0	1.23E-02	0.071728734	6.61E-02	0.014845957	0.43166156	8.84E-02	0.00517591	5.84E-02	0	0	2.06E-03	EGFR_Wt
TCGA-MP-A4T8-01	1.48E-02	0	0.035649872	0.0218	0	0.017462665	0.00E+00	0.06498382	8.90E-02	5.40E-03	0	2.76E-02	0	3.88E-01	0	0.18146159	0.00E+00	0.127590819	2.66E-02	0	0	0.00E+00	EGFR_Wt
TCGA-78-7166-01	0.00E+00	0.058025169	0.143081336	0.0821	0	0.149336681	0.00E+00	0.115999626	6.03E-02	0.00E+00	0	3.62E-02	0	1.31E-01	0.10246736	0.11912137	0.00E+00	0	1.96E-03	0	0	0.00E+00	EGFR_Wt
TCGA-75-5146-01	0.00E+00	0.07034259	0.207155869	0.0557	0	0.221013587	0.00E+00	0.076127667	1.63E-02	3.28E-03	0	3.04E-02	0.00307065	0.00E+00	0.030851905	0.25428906	5.29E-03	0.004569499	1.27E-02	0	0	8.92E-03	EGFR_Wt
TCGA-49-4510-01	6.45E-03	0	0.24286785	0.115	0	0.057953093	0.00E+00	0.081155795	1.88E-03	0.00E+00	0	3.63E-02	0.022924001	0.00E+00	0.017731156	0.36885121	4.34E-03	0.01353222	2.29E-02	0	0	8.29E-03	EGFR_Wt
TCGA-78-7539-01	0.00E+00	0.010241272	0.021232506	0.136	0	0.271969447	0.00E+00	0.041592127	1.26E-02	0.00E+00	0	5.97E-02	0.026088896	4.92E-02	0.100203853	0.1837081	4.66E-02	0	4.13E-02	0	0	0.00E+00	EGFR_Wt
TCGA-64-5775-01	2.97E-03	0	0.010959941	0.0458	0	0.085295494	0.00E+00	0.013645143	1.25E-02	0.00E+00	0	5.81E-02	0.034707373	1.39E-01	0.012388283	0.51210101	0.00E+00	0	0.00E+00	0.065445041	0	7.43E-03	EGFR_Wt
TCGA-55-6712-01	1.66E-02	0.017075173	0.002528757	0.132	0	0.164804802	2.57E-02	0.00920382	1.49E-02	5.19E-02	0	2.76E-02	0	1.39E-01	0.102718009	0.23218973	0.00E+00	0	6.39E-02	0	0	0.00E+00	EGFR_Wt
TCGA-55-8087-01	0.00E+00	0.009183503	0.028021264	0.0358	0	0.154696498	0.00E+00	0.002003983	8.14E-03	0.00E+00	0	1.52E-02	0.034591418	1.56E-02	0.011781374	0.31457247	2.38E-01	0	1.31E-01	0	0	2.12E-03	EGFR_Wt
TCGA-05-5428-01	2.22E-02	0	0.016737799	0.0674	0	0.068256903	0.00E+00	0.026453555	0.00E+00	0.00E+00	0	1.42E-02	0.00791445	0.00E+00	0.031282663	0.56639779	4.41E-02	0	9.93E-02	0.01617342	0	1.95E-02	EGFR_Wt
TCGA-05-4425-01	8.67E-03	0.001017112	0	0.00561	0	0.100507106	0.00E+00	0.033945566	5.01E-02	1.04E-02	0	5.45E-03	0.002269997	1.92E-01	0.053526221	0.44898734	5.94E-02	0	1.82E-02	0.007237208	0	3.04E-03	EGFR_Wt
TCGA-55-A493-01	2.74E-02	0.022001585	0.016009296	0.164	0	0.155497535	5.71E-02	0.028300602	1.98E-02	0.00E+00	0	6.99E-02	0.017610203	6.16E-02	0.105162012	0.20627941	2.52E-02	0	0.00E+00	0.021072565	0	2.66E-03	EGFR_Wt
TCGA-97-A4LX-01	2.87E-02	0.01067887	0.051867451	0.108	0	0.140329791	0.00E+00	0.061037638	6.11E-02	0.00E+00	0	4.57E-02	0.025660649	4.55E-03	0.122418857	0.26950105	2.20E-02	0	4.30E-02	0	0	5.13E-03	EGFR_Wt
TCGA-75-6206-01	2.69E-03	0	0.025256107	0.0487	0	0.061074925	0.00E+00	0.08166292	5.61E-03	0.00E+00	0.001715714	6.35E-03	0	2.88E-01	0.002278562	0.41846343	0.00E+00	0.028244956	3.04E-02	0	0	0.00E+00	EGFR_Wt
TCGA-55-7914-01	0.00E+00	0.032508448	0.212833235	0.13	0	0.213887992	0.00E+00	0.05197808	1.64E-02	0.00E+00	0	2.87E-02	0.00773	0.00E+00	0.101040538	0.16799847	0.00E+00	0.000667876	3.63E-02	0	0	0.00E+00	EGFR_Wt
TCGA-L9-A5IP-01	0.00E+00	0.001962966	0.090772771	0.132	0	0.117760892	0.00E+00	0.077470529	3.74E-02	0.00E+00	0	8.23E-02	0	1.21E-01	0.037568496	0.22432628	0.00E+00	0	1.68E-02	0	0	6.12E-02	EGFR_Wt
TCGA-05-5423-01	1.99E-02	0.001945691	0.040100579	0.0373	0	0.095178695	6.21E-03	0.033130521	2.48E-02	1.52E-02	0	3.69E-02	0.041453073	0.00E+00	0.002540461	0.36281328	1.24E-01	0.062361883	6.17E-02	0	0.01	2.14E-02	EGFR_Wt
TCGA-44-6145-01	3.38E-03	0.027489237	0.133250805	0.219	0	0.059948727	2.50E-02	0.072270198	1.45E-03	0.00E+00	0	5.65E-02	0.004762622	1.50E-02	0.124094298	0.22713818	6.80E-04	0	7.85E-03	0	0	2.17E-02	EGFR_Wt
TCGA-91-6836-01	6.67E-03	0	0.105504232	0.147	0	0.081203666	9.98E-02	0.033863047	3.87E-04	0.00E+00	0	5.81E-02	0.002999972	1.03E-01	0.011897135	0.17631016	3.07E-02	0.031177214	0.00E+00	0.100115918	0	1.09E-02	EGFR_Wt
TCGA-44-7671-01	0.00E+00	0.015130132	0.232121928	0.041	0	0.098406084	0.00E+00	0.033376303	0.00E+00	0.00E+00	0	1.95E-02	0.013084716	6.29E-02	0.007912912	0.36779207	1.02E-03	0.048238844	5.09E-02	0	0	8.58E-03	EGFR_Wt
TCGA-S2-AA1A-01	5.04E-02	0.028401196	0.162287919	0.0768	0	0.210319879	0.00E+00	0.055075982	3.51E-02	0.00E+00	0	4.25E-02	0.022571269	5.42E-02	0.060923003	0.11894737	2.83E-02	0	5.42E-02	0	0	0.00E+00	EGFR_Wt
TCGA-73-7499-01	7.42E-03	0.006242291	0.058327054	0.244	0	0.005773761	1.81E-02	0.052168344	7.67E-02	6.52E-02	0	4.84E-02	0	7.56E-04	0.151394237	0.23239155	1.25E-02	0	2.03E-02	0	0	0.00E+00	EGFR_Wt
TCGA-49-AARN-01	0.00E+00	0.162882379	0.10084632	0.0734	0	0.143617759	0.00E+00	0.079775068	8.00E-02	0.00E+00	0	5.83E-02	0.010711393	2.20E-02	0.038901352	0.15794004	1.30E-02	0.01080974	4.78E-02	0	0	0.00E+00	EGFR_Wt
TCGA-05-4433-01	5.09E-03	0.012995149	0	0.118	0	0.117097681	0.00E+00	0.058608896	3.26E-02	0.00E+00	0	4.39E-02	0.025614632	1.84E-01	0.07515053	0.25552792	5.96E-03	0	6.57E-02	0	0	0.00E+00	EGFR_Wt
TCGA-49-6761-01	0.00E+00	0.039374595	0.035851327	0.144	0	0.06411029	5.25E-02	0.083688266	1.11E-02	0.00E+00	0	1.29E-01	0.016831252	6.12E-02	0.110745074	0.20090745	0.00E+00	0.004439937	0.00E+00	0.042309395	0	4.11E-03	EGFR_Wt
TCGA-86-7711-01	7.66E-03	0	0.029588481	0.0387	0	0.155604362	0.00E+00	0.002879482	2.29E-02	0.00E+00	0.055303201	0.00E+00	0.01876052	2.34E-01	0.031435262	0.16319168	0.00E+00	0	0.00E+00	0.2083967	0	3.17E-02	EGFR_Wt
TCGA-50-5068-01	2.61E-03	0.044001368	0.081641244	0.075	0	0.094616687	0.00E+00	0.067976947	4.25E-03	1.22E-01	0	4.69E-02	0.002210833	1.78E-02	0.089258465	0.20328097	8.73E-02	0	6.08E-02	0	0	0.00E+00	EGFR_Wt
TCGA-67-4679-01	0.00E+00	0.009424594	0	0.0795	0	0.146506637	0.00E+00	0.006266517	1.90E-02	0.00E+00	0	2.30E-02	0.157386449	0.00E+00	0.01623095	0.23840229	8.86E-02	0.006024058	2.10E-01	0	0	0.00E+00	EGFR_Wt
TCGA-44-2665-01	0.00E+00	0.037565284	0.01793388	0.0382	0	0.176276493	0.00E+00	0.013120908	0.00E+00	0.00E+00	0.021048903	2.06E-02	0.020912435	8.41E-02	0	0.40510676	6.43E-02	0.046544408	4.65E-02	0	0	7.84E-03	EGFR_Wt
TCGA-44-3919-01	1.05E-02	0.013709089	0.061964375	0.0955	0	0.178290124	0.00E+00	0.038583852	3.06E-02	0.00E+00	0	3.90E-02	0.003677488	1.56E-03	0.15178375	0.2466877	5.23E-02	0	7.17E-02	0	0	4.11E-03	EGFR_Wt
TCGA-55-6987-01	8.12E-03	0.035253955	0.125357652	0.219	0	0.023033841	3.91E-02	0.104672101	9.36E-03	0.00E+00	0.009899316	3.50E-02	0.024053584	8.09E-02	0.13417926	0.1355323	3.15E-04	0	0.00E+00	0.012993697	0	3.03E-03	EGFR_Wt
TCGA-91-6829-01	3.57E-03	0.008804754	0.001266298	0.0743	0	0.022014981	0.00E+00	0.012641106	3.86E-03	0.00E+00	0	2.72E-02	0.017424679	1.20E-01	0.015412253	0.50573264	0.00E+00	0	1.79E-01	0	0	9.32E-03	EGFR_Wt
TCGA-05-4396-01	5.80E-02	0	0.082192577	0.0668	0	0.183357466	0.00E+00	0.085589418	2.08E-02	0.00E+00	0	2.59E-02	0.018301969	4.03E-02	0.068353787	0.30437418	2.93E-03	0	2.87E-02	0	0	1.45E-02	EGFR_Wt
TCGA-62-8399-01	1.68E-02	0	0.076769963	0.109	0	0.134081096	1.12E-02	0.055597098	3.11E-03	0.00E+00	0.010976705	3.85E-02	0.112549286	0.00E+00	0.029066174	0.23137651	0.00E+00	0.070491482	2.69E-02	0.043360902	0	3.03E-02	EGFR_Wt
TCGA-86-8358-01	5.20E-02	0	0.111201776	0.119	0	0.024193261	0.00E+00	0.126978236	0.00E+00	0.00E+00	0	4.67E-02	0.005290544	1.37E-01	0.0785749	0.28540746	8.34E-05	0	0.00E+00	0.012903999	0	0.00E+00	EGFR_Wt
TCGA-MP-A4TK-01	1.04E-02	0.034971711	0.094009674	0.0518	0	0.102609521	0.00E+00	0.070105202	2.24E-02	0.00E+00	0.005149957	0.00E+00	0	2.80E-01	0.045716585	0.27220701	0.00E+00	0	9.88E-03	0	0	7.16E-04	EGFR_Wt
TCGA-44-7667-01	0.00E+00	0.005224564	0.056926009	0.0776	0	0.063225488	0.00E+00	0.012484903	1.37E-02	0.00E+00	0	9.29E-02	0.008218251	3.34E-01	0.075923119	0.20516372	0.00E+00	0	0.00E+00	0.054175012	0	0.00E+00	EGFR_Wt
TCGA-97-8174-01	2.98E-02	0	0.071149216	0.0552	0	0.205470704	0.00E+00	0.019052732	4.37E-03	0.00E+00	0.00873391	1.14E-02	0.036463276	0.00E+00	0.084784867	0.40113084	0.00E+00	0.006021223	6.64E-02	0	0	0.00E+00	EGFR_Wt
TCGA-44-A4SS-01	2.11E-02	0	0.01109566	0.151	0	0.213925591	4.38E-03	0.062978737	5.12E-03	0.00E+00	0.010897127	2.33E-02	0.01360014	2.82E-02	0.110142287	0.32644904	3.52E-03	0	1.41E-02	0	0	0.00E+00	EGFR_Wt
TCGA-NJ-A55O-01	0.00E+00	0.028316126	0.007513522	0.0446	0	0.199984661	0.00E+00	0.048588632	1.52E-02	0.00E+00	0	3.04E-02	0.007305736	8.32E-02	0.058127453	0.40843919	4.91E-03	0.009147215	4.74E-02	0	0	6.98E-03	EGFR_Wt
TCGA-78-7159-01	7.92E-03	0.014179761	0.207644938	0.0849	0	0.197883706	0.00E+00	0.015033109	5.32E-02	0.00E+00	0	6.41E-02	0	8.53E-02	0.052993703	0.11848424	0.00E+00	0.006273128	0.00E+00	0.053295941	0	3.88E-02	EGFR_Wt
TCGA-78-7535-01	0.00E+00	0.041928228	0.012498345	0.0377	0	0.096528553	0.00E+00	0.014952999	3.74E-03	0.00E+00	0.020044055	7.38E-03	0.013924789	7.01E-02	0.002717962	0.63431323	2.50E-02	0.002291302	0.00E+00	0.016486261	0	4.38E-04	EGFR_Wt
TCGA-91-8499-01	0.00E+00	0.106957432	0	0.0933	0	0.052374177	5.23E-03	0.11397318	0.00E+00	2.07E-02	0	5.83E-02	0.018230641	4.39E-02	0.108593345	0.30595523	2.44E-02	0	4.81E-02	0	0	0.00E+00	EGFR_Wt
TCGA-44-5644-01	2.37E-02	0	0.157044115	0.105	0	0.127946294	0.00E+00	0.11236776	2.53E-03	0.00E+00	0.057865132	5.04E-03	0	1.00E-01	0.025065216	0.1725962	0.00E+00	0.006328957	0.00E+00	0.079376635	0	2.51E-02	EGFR_Wt
TCGA-55-8614-01	0.00E+00	0.013256935	0.110669939	0.0837	0	0.106012223	0.00E+00	0.05109806	1.87E-02	0.00E+00	0	5.38E-02	0.009837681	1.38E-01	0.096415289	0.22985425	9.13E-03	0	7.05E-02	0	0	8.61E-03	EGFR_Wt
TCGA-55-7910-01	2.03E-02	0	0.323346925	0.0267	0	0.171800712	0.00E+00	0.090497541	8.37E-03	0.00E+00	0.013874207	0.00E+00	0.025883032	7.29E-02	0.019958479	0.09133011	0.00E+00	0.062680388	0.00E+00	0.05046696	0	2.20E-02	EGFR_Wt
TCGA-73-4668-01	0.00E+00	0.023088434	0.139348804	0.0905	0	0.07684565	0.00E+00	0.027456502	1.13E-02	0.00E+00	0	5.57E-03	0	2.92E-01	0.04276731	0.2241782	0.00E+00	0.0051633	6.15E-02	0	0	0.00E+00	EGFR_Wt
TCGA-69-7978-01	0.00E+00	0.014278848	0.044029484	0.119	0	0.15407377	0.00E+00	0.032503027	4.87E-02	0.00E+00	0	5.27E-02	0.011204156	3.09E-02	0.085471658	0.29839732	8.35E-02	0	1.14E-02	0.000837056	0	1.34E-02	EGFR_Wt
TCGA-44-4112-01	5.26E-03	0.001990795	0.134545219	0.0898	0	0.113334732	4.52E-02	0.021764254	0.00E+00	5.47E-03	0	1.25E-02	0.023884413	2.58E-03	0.02337239	0.4027634	1.33E-02	0.060310513	1.60E-02	0.014407357	0	1.34E-02	EGFR_Wt
TCGA-73-4675-01	6.15E-03	0.027464345	0.000775647	0.0303	0	0.056795762	0.00E+00	0.015013735	3.26E-02	0.00E+00	0	2.00E-02	0.050199543	2.67E-01	0	0.30207214	7.32E-02	0.035909884	8.18E-02	0	0	8.83E-04	EGFR_Wt
TCGA-50-5933-01	0.00E+00	0.002362806	0.014189859	0.044	0	0.12278392	0.00E+00	0.022192294	0.00E+00	0.00E+00	0	1.91E-02	0.066600518	1.27E-01	0.052934399	0.46355335	2.66E-03	0	0.00E+00	0.058440558	0	4.05E-03	EGFR_Wt
TCGA-50-6592-01	5.10E-02	0	0.044600614	0.168	0	0.102248789	1.22E-02	0.0438633	3.15E-03	0.00E+00	0	6.03E-02	0.007823311	1.83E-01	0.104893427	0.17357607	0.00E+00	0	0.00E+00	0.03720369	0	7.99E-03	EGFR_Wt
TCGA-44-6146-01	2.88E-02	0	0.082265303	0.123	0	0.291240164	0.00E+00	0.036497665	0.00E+00	0.00E+00	0.01510371	1.77E-03	0.047489586	0.00E+00	0.06299319	0.22507441	0.00E+00	0.009487378	5.15E-02	0	0	2.52E-02	EGFR_Wt
TCGA-05-4432-01	0.00E+00	0.022269417	0.133105184	0.097	0	0.119890596	0.00E+00	0.052131079	4.49E-02	0.00E+00	0	5.49E-02	0.014139814	1.40E-01	0.095065506	0.19030178	0.00E+00	0	0.00E+00	0.019259247	0	1.67E-02	EGFR_Wt
TCGA-35-4123-01	0.00E+00	0.016142525	0.048445867	0.287	0	0.008190931	3.03E-02	0.074308507	5.86E-03	0.00E+00	0	7.56E-02	0.032963023	1.28E-02	0.039065976	0.27458309	2.08E-02	0	0.00E+00	0.073739879	0	0.00E+00	EGFR_Wt
TCGA-49-AAR2-01	0.00E+00	0.035322513	0.293570792	0.105	0	0.123411535	0.00E+00	0.055647389	5.56E-02	0.00E+00	0	3.36E-02	0.00954212	1.86E-02	0.040839598	0.21149083	0.00E+00	0	9.01E-03	0	0	7.88E-03	EGFR_Wt
TCGA-49-AARO-01	1.78E-02	0.050631469	0.029946357	0.073	0	0.112672667	0.00E+00	0.081861638	3.76E-02	0.00E+00	0	3.47E-02	0.03059663	1.72E-01	0.033976181	0.24190767	4.08E-02	0.02817835	0.00E+00	0.010416924	0	3.81E-03	EGFR_Wt
TCGA-75-6211-01	5.07E-02	0	0.239520379	0.113	0	0.126949312	6.22E-02	0.073241591	3.51E-02	0.00E+00	0.018453682	0.00E+00	0.007930027	1.17E-02	0.02030257	0.22684668	6.13E-03	0	3.87E-04	0	0	7.13E-03	EGFR_Wt
TCGA-55-8085-01	4.95E-03	0.038561888	0.06345802	0.133	0	0.111551198	0.00E+00	0.092126052	5.08E-02	0.00E+00	0	6.08E-02	0.054633117	9.08E-02	0.078019045	0.20657709	0.00E+00	0.00052452	0.00E+00	0.014178308	0	0.00E+00	EGFR_Wt
TCGA-80-5607-01	1.21E-03	0.016606532	0.06472437	0.15	0	0.107083014	0.00E+00	0.071831484	1.61E-03	0.00E+00	0	8.16E-02	0.017970913	0.00E+00	0.110037556	0.25592524	3.23E-02	0	8.68E-02	0	0	2.01E-03	EGFR_Wt

TCGA-55-7728-01	1.48E-02	0.030142063	0	0.0359	0	0.038350798	0.00E+00	0.045302571	5.37E-02	0.00E+00	0.013318896	7.10E-03	0	4.97E-01	0.02464635	0.22420774	0.00E+00	0	1.54E-02	0	0	0.00E+00	EGFR_Wt
TCGA-64-5815-01	0.00E+00	0.038427093	0.020883322	0.0271	0	0.10813557	2.95E-03	0.014936603	2.11E-02	0.00E+00	0.007845513	0.00E+00	0.022456016	3.27E-01	0.00080806	0.31150877	1.50E-02	0.04880341	3.33E-02	0	0	5.35E-05	EGFR_Wt
TCGA-NJ-A4YI-01	0.00E+00	0.013260178	0.009432908	0.0337	0	0.120030394	0.00E+00	0.050819534	1.56E-02	0.00E+00	0.0196219	2.15E-02	0.105636291	9.32E-02	0.008950223	0.35738513	8.74E-02	0	2.68E-02	0	0	3.67E-02	EGFR_Wt
TCGA-55-7284-01	1.20E-02	0.001299944	0	0.112	0	0.13063113	0.00E+00	0.034978721	5.42E-02	0.00E+00	0	2.30E-02	0.03631396	1.60E-01	0.057389054	0.29943797	1.25E-02	0	6.66E-02	0	0	0.00E+00	EGFR_Wt
TCGA-78-7149-01	0.00E+00	0	0.360694902	0.0898	0	0.131114865	0.00E+00	0.081753355	1.20E-02	0.00E+00	0	6.86E-02	0	0.00E+00	0.078882972	0.14171635	0.00E+00	0	2.28E-02	0	0	1.26E-02	EGFR_Wt
TCGA-86-8674-01	5.10E-02	0	0.10443003	0.0508	0	0.029852913	0.00E+00	0.200331066	1.77E-03	0.00E+00	0.001927531	1.71E-02	0	3.62E-01	0.024211108	0.08845643	0.00E+00	0.054870845	0.00E+00	0.013218039	0	0.00E+00	EGFR_Wt
TCGA-86-6851-01	0.00E+00	0.012463894	0.17501011	0.135	0	0.133223484	1.85E-02	0.083932763	1.64E-02	0.00E+00	0.006456459	2.52E-02	0.005794671	9.27E-03	0.117900699	0.23728762	8.47E-03	0	1.53E-02	0	0	0.00E+00	EGFR_Wt
TCGA-86-8673-01	0.00E+00	0.024370103	0.11649587	0.173	0	0.030016749	0.00E+00	0.053154595	5.11E-02	0.00E+00	0	7.32E-02	0	2.05E-01	0.03466683	0.2130085	0.00E+00	0.009529073	0.00E+00	0.011269395	0	4.63E-03	EGFR_Wt
TCGA-49-4507-01	1.62E-02	0	0.068380345	0.324	0	0.029500569	1.74E-02	0.06286775	4.80E-02	0.00E+00	0	1.49E-01	0.002355009	0.00E+00	0.065115132	0.16930599	1.05E-02	0	0.00E+00	0.037767243	0	0.00E+00	EGFR_Wt
TCGA-L9-A443-01	1.08E-02	0.01276476	0.049985564	0.123	0	0.152799382	0.00E+00	0.082178268	5.09E-04	3.42E-02	0	4.08E-02	0.00022547	4.25E-02	0.099341379	0.28571949	9.54E-03	0	5.52E-02	0	0	0.00E+00	EGFR_Wt
TCGA-55-1592-01	2.75E-02	0	0.082655088	0.0385	0	0.085613079	0.00E+00	0.045634852	0.00E+00	0.00E+00	0	1.61E-02	0.076304989	1.66E-01	0.047365465	0.32175285	3.74E-02	0	2.01E-02	0.027275455	0	8.06E-03	EGFR_Wt
TCGA-55-A4DF-01	8.89E-03	0.040293257	0.002335856	0.123	0	0.145365751	2.93E-02	0.067792479	0.00E+00	0.00E+00	0	6.06E-02	0.030633943	1.60E-01	0.150026442	0.15026646	3.40E-03	0	2.82E-02	0	0	0.00E+00	EGFR_Wt
TCGA-69-A59K-01	2.16E-02	0	0.287003058	0.0797	0	0.075261275	8.43E-03	0.04767203	6.19E-03	0.00E+00	0.011835255	0.00E+00	0.011867987	3.95E-02	0.08253108	0.30535118	1.26E-02	0	1.56E-03	0.008917174	0	0.00E+00	EGFR_Wt
TCGA-78-7162-01	1.50E-02	0.029962525	0.057955872	0.12	0	0.187802614	0.00E+00	0.084852729	9.04E-03	0.00E+00	0	4.52E-02	0.006441388	0.00E+00	0.064898911	0.2603245	1.46E-02	0.025855604	7.77E-02	0	0	0.00E+00	EGFR_Wt
TCGA-05-4434-01	2.15E-02	0.005477419	0.013140397	0.0487	0	0.177637193	0.00E+00	0.038570725	3.19E-02	0.00E+00	0	2.52E-02	0.023891627	3.51E-02	0.150167654	0.3541102	2.27E-02	0	4.82E-02	0	0	3.81E-03	EGFR_Wt
TCGA-05-4249-01	7.51E-03	0.010959033	0.041285851	0.0316	0	0.083655869	0.00E+00	0.036744116	2.30E-02	0.00E+00	0	1.87E-02	0.032868625	0.00E+00	0.021189255	0.31220194	2.40E-01	0.005354526	1.16E-01	0	0	1.84E-02	EGFR_Wt
TCGA-44-7661-01	3.21E-02	0	0	0.0477	0	0.213637582	0.00E+00	0.039814266	2.19E-02	0.00E+00	0	2.40E-02	0.10848951	2.65E-02	0.071179841	0.32457133	0.00E+00	0.078231714	1.19E-02	0	0	0.00E+00	EGFR_Wt
TCGA-64-5778-01	1.26E-02	0.03077743	0.073042087	0.241	0	0.02955094	0.00E+00	0.092119943	4.04E-02	5.82E-02	0	8.04E-02	0	1.80E-02	0.120912826	0.19243867	0.00E+00	0	1.03E-02	0	0	0.00E+00	EGFR_Wt
TCGA-75-7031-01	4.42E-03	0	0.082611179	0.184	0	0.101646931	0.00E+00	0.080918093	1.26E-02	0.00E+00	0	5.80E-02	0.011813306	2.03E-02	0.113938737	0.30258023	9.77E-03	0	0.00E+00	0.007943836	0	9.43E-03	EGFR_Wt
TCGA-73-4658-01	0.00E+00	0.005296873	0.052541547	0.0575	0	0.127410093	1.83E-02	0.04391812	7.56E-03	8.47E-03	0	1.84E-02	0.055631281	0.00E+00	0.057888947	0.38181638	8.64E-02	0.006187141	0.00E+00	0.066023269	0	6.61E-03	EGFR_Wt
TCGA-J2-A4AD-01	0.00E+00	0	0.15100522	0.203	0	0	0.00E+00	0.03031983	1.59E-02	0.00E+00	0	0.00E+00	0.009688365	1.77E-01	0	0.22510862	2.24E-02	0.033769597	1.24E-01	0	0	6.59E-03	EGFR_Wt
TCGA-69-8255-01	1.45E-02	0	0.086326762	0.303	0	0	1.22E-01	0.126030024	0.00E+00	1.48E-02	0	4.19E-02	0.0005274	4.18E-02	0.080773256	0.16001507	9.51E-04	0	0.00E+00	0.007031793	0	0.00E+00	EGFR_Wt
TCGA-55-8505-01	0.00E+00	0.006871718	0.205676525	0.0305	0	0.065503543	0.00E+00	0.023233904	2.21E-02	0.00E+00	0	1.22E-02	0	3.71E-01	0.022003928	0.19644545	0.00E+00	0	4.72E-03	0.035663268	0	4.08E-03	EGFR_Wt
TCGA-55-7907-01	1.07E-02	0	0.055352347	0.157	0	0.116479669	0.00E+00	0.066002403	2.82E-02	0.00E+00	0	3.26E-02	0.023759872	5.29E-02	0.164403051	0.25017079	1.38E-02	0	2.88E-02	0	0	0.00E+00	EGFR_Wt
TCGA-75-6212-01	1.01E-01	0	0.098198471	0.0444	0	0.109650658	0.00E+00	0.026078522	2.10E-02	0.00E+00	0	2.44E-02	0.005158784	0.00E+00	0.009493604	0.3103317	1.19E-01	0.006204603	1.10E-01	0	0	1.56E-02	EGFR_Wt
TCGA-55-8205-01	1.27E-02	0.02227503	0.003174824	0.14	0	0.067787388	4.88E-02	0.052453058	0.00E+00	0.00E+00	0	8.14E-02	0.053189117	0.00E+00	0.085118596	0.39846619	1.79E-02	0.001214251	0.00E+00	0.010205785	0	5.79E-03	EGFR_Wt
TCGA-86-8672-01	0.00E+00	0.023104706	0.068868194	0.215	0	0.006034518	5.10E-02	0.080688657	1.87E-02	0.00E+00	0	7.40E-02	0.023403945	4.27E-02	0.081802278	0.24795914	1.11E-02	0	0.00E+00	0.048054538	0	7.70E-03	EGFR_Wt
TCGA-55-8097-01	0.00E+00	0.059295897	0.063180371	0.0676	0	0.144872658	0.00E+00	0.067549995	3.45E-02	0.00E+00	0	3.48E-02	0.029749201	0.00E+00	0.015997097	0.28369857	6.33E-02	0.009222793	1.26E-01	0	0	0.00E+00	EGFR_Wt
TCGA-NJ-A4YP-01	0.00E+00	0.011588294	0.060946668	0.137	0	0.032826253	0.00E+00	0.084436725	2.91E-02	0.00E+00	0	5.72E-02	0.001791747	1.31E-01	0.068230639	0.25823994	0.00E+00	0.086455636	3.44E-02	0.000261018	0	6.53E-03	EGFR_Wt
TCGA-38-7271-01	0.00E+00	0.236423046	0.05295665	0.139	0	0.149891928	0.00E+00	0.000200424	5.02E-02	2.84E-02	0	2.47E-02	0.01315132	6.56E-02	0.031217527	0.17930734	0.00E+00	0.009671358	1.94E-02	0	0	0.00E+00	EGFR_Wt
TCGA-86-8281-01	2.20E-03	0.03588651	0.223717265	0.0799	0	0.147357774	0.00E+00	0.060568915	3.53E-02	0.00E+00	0	4.65E-02	0.019689342	3.65E-02	0.030504512	0.1966792	2.79E-02	0.009956106	4.73E-02	0	0	0.00E+00	EGFR_Wt
TCGA-55-A494-01	2.07E-02	0	0.094236047	0.175	0.05	0	0.00E+00	0.044648197	1.08E-01	0.00E+00	0	3.23E-02	0.020597091	1.38E-01	0	0.19553819	0.00E+00	0.117723822	0.00E+00	0.000926025	0	3.96E-03	EGFR_Wt
TCGA-05-4422-01	0.00E+00	0.032670367	0.181941585	0.204	0	0.156795313	0.00E+00	0.053973381	2.03E-02	0.00E+00	0	3.34E-02	0.012488411	1.62E-02	0.046882422	0.23946097	0.00E+00	0	0.00E+00	0.002211821	0	0.00E+00	EGFR_Wt
TCGA-55-7913-01	0.00E+00	0.002008564	0.09304166	0.0807	0	0.042696729	0.00E+00	0.091429764	7.56E-02	0.00E+00	0	2.17E-02	0	3.37E-01	0.037955008	0.13662914	2.01E-02	0	0.00E+00	0.042728132	0	1.81E-02	EGFR_Wt
TCGA-49-6745-01	0.00E+00	0.007301904	0.013662585	0.122	0	0.140984914	2.59E-02	0.058810338	4.40E-03	0.00E+00	0	3.37E-02	0.067445852	3.25E-02	0.096615932	0.30544272	1.51E-02	0.022930124	5.01E-02	0	0	3.33E-03	EGFR_Wt
TCGA-50-8457-01	0.00E+00	0.063584829	0.185847455	0.0853	0	0.108620957	0.00E+00	0.044286029	2.98E-02	0.00E+00	0	5.32E-02	0.089322786	0.00E+00	0.037307263	0.21773459	1.12E-02	0	7.39E-02	0	0	0.00E+00	EGFR_Wt
TCGA-97-8176-01	1.39E-02	0.005642881	0.046722332	0.0559	0	0.061035746	0.00E+00	0.061506142	6.53E-03	0.00E+00	0.026592774	0.00E+00	0.050899916	1.09E-01	0.018174903	0.46125862	0.00E+00	0.004371985	4.82E-02	0	0	3.05E-02	EGFR_Wt
TCGA-73-4670-01	0.00E+00	0.030610979	0.141405325	0.078	0	0.040853447	0.00E+00	0.057629289	2.73E-02	0.00E+00	0	4.25E-02	0.032009771	1.48E-01	0	0.22333652	0.00E+00	0.101598943	0.00E+00	0.041209673	0	3.51E-02	EGFR_Wt
TCGA-50-5055-01	5.87E-02	0.066315175	0.08000228	0.113	0	0.195592387	0.00E+00	0.043374789	2.71E-02	4.30E-02	0	2.29E-02	0.00672363	8.99E-02	0.034792548	0.16299247	3.23E-02	0.004666881	1.90E-02	0	0	0.00E+00	EGFR_Wt
TCGA-95-A4VN-01	1.15E-03	0.023113229	0	0.139	0	0.152657568	1.71E-03	0.087778872	1.08E-02	0.00E+00	0	5.52E-02	0.019859853	4.51E-02	0.117696223	0.29769036	4.12E-03	0.006032178	2.54E-02	0	0	1.29E-02	EGFR_Wt
TCGA-44-7659-01	0.00E+00	0.088596442	0.080710942	0.0911	0	0.173388535	0.00E+00	0.032667237	2.72E-02	0.00E+00	0	5.01E-02	0.022912391	3.02E-02	0.01710491	0.27078939	1.05E-02	0.028614748	6.66E-02	0	0	9.57E-03	EGFR_Wt
TCGA-NJ-A7XG-01	0.00E+00	0.010567831	0.052576543	0.0847	0	0.250851771	0.00E+00	0.030422957	2.83E-02	0.00E+00	0	8.65E-02	0.017984048	0.00E+00	0.010912929	0.15009836	3.59E-02	0.034844414	2.06E-01	0	0	0.00E+00	EGFR_Wt
TCGA-97-8179-01	4.90E-03	0	0.10910912	0.0822	0	0.046343757	0.00E+00	0.066718858	1.26E-02	0.00E+00	0	6.08E-02	0.032769705	0.00E+00	0.02770742	0.38298423	2.89E-02	0.020010675	1.14E-01	0	0	1.14E-02	EGFR_Wt
TCGA-44-8120-01	1.39E-02	0.028796257	0.073346589	0.0593	0	0.140227614	0.00E+00	0.025970966	3.99E-02	0.00E+00	0	3.84E-02	0.03091525	5.22E-02	0.014589058	0.3295015	5.36E-02	0.012225988	7.75E-02	0	0	9.62E-03	EGFR_Wt
TCGA-44-3398-01	1.81E-03	0.015275445	0.045876423	0.0309	0	0.19234799	0.00E+00	0.020013809	7.70E-03	0.00E+00	0	3.85E-02	0.023544273	0.00E+00	0.030718749	0.26160575	2.22E-01	0.027924282	6.50E-02	0	0	1.66E-02	EGFR_Wt
TCGA-NJ-A55R-01	0.00E+00	0.124140743	0.230321433	0.0236	0	0.038583856	0.00E+00	0.078133803	3.19E-02	0.00E+00	0	2.53E-02	0	2.27E-01	0.010855973	0.15532589	0.00E+00	0	5.53E-02	0	0	0.00E+00	EGFR_Wt
TCGA-55-8511-01	9.93E-03	0.059741505	0.066544032	0.109	0	0.144502535	0.00E+00	0.084699362	3.65E-02	0.00E+00	0	4.59E-02	0.019037808	7.07E-02	0.082617324	0.25973986	0.00E+00	0	1.08E-02	0	0	0.00E+00	EGFR_Wt
TCGA-MN-A4N4-01	7.81E-03	0.008786091	0.023539006	0.044	0	0.052098233	7.67E-03	0.051044514	6.84E-03	3.76E-03	0.015599559	0.00E+00	0	4.91E-01	0.021173813	0.20727596	0.00E+00	0	5.23E-02	0.001785654	0	5.18E-03	EGFR_Wt
TCGA-MP-A4TE-01	1.15E-02	0	0.319020991	0.0981	0	0.00243498	0.00E+00	0.047628786	6.77E-02	0.00E+00	0.018556385	0.00E+00	0	9.45E-02	0	0.18786322	0.00E+00	0.002617033	0.00E+00	0.08635601	0	6.38E-02	EGFR_Wt
TCGA-55-7574-01	1.98E-02	0.150179132	0.084954731	0.127	0	0.193672478	0.00E+00	0.053509546	4.55E-03	0.00E+00	0	2.99E-02	0.009964362	3.03E-02	0.04428529	0.20966027	0.00E+00	0.016028352	2.58E-02	0	0	9.40E-05	EGFR_Wt
TCGA-99-8025-01	0.00E+00	0.059235747	0.102294096	0.034	0	0.120071355	0.00E+00	0.046908625	2.35E-02	0.00E+00	0	2.55E-02	0	2.22E-01	0.055966246	0.28969784	0.00E+00	0	2.09E-02	0	0	0.00E+00	EGFR_Wt
TCGA-55-8299-01	3.20E-03	0.005533001	0.041016004	0.215	0	0.019789312	3.71E-03	0.096747382	1.47E-02	0.00E+00	0.007332538	5.17E-02	0.034377084	0.00E+00	0.130686487	0.2159484	0.00E+00	0.142148549	1.13E-02	0	0	7.14E-03	EGFR_Wt
TCGA-38-4626-01	7.15E-03	0	0.018299209	0.0462	0	0.062719981	0.00E+00	0.024325306	0.00E+00	0.00E+00	0.011010733	4.36E-03	0.101930101	1.45E-01	0	0.45784709	0.00E+00	0.047680404	4.39E-02	0	0	2.93E-02	EGFR_Wt
TCGA-L4-A4E5-01	0.00E+00	0.010482015	0.133974277	0.105	0	0.093082482	0.00E+00	0.109706397	8.07E-02	0.00E+00	0	6.64E-02	0	1.47E-01	0.046220389	0.19225396	4.05E-03	0	0.00E+00	0.011873829	0	0.00E+00	EGFR_Wt
TCGA-MP-A4TC-01	8.57E-03	0	0.026400653	0.0725	0	0.04720219	3.86E-03	0.032415008	3.27E-02	1.28E-02	0	1.50E-02	0	2.22E-01	0.074352246	0.31485348	4.65E-03	0	0.00E+00	0.123370485	0	9.38E-03	EGFR_Wt
TCGA-95-8494-01	0.00E+00	0.04787027	0.000584204	0.0354	0	0.111873667	0.00E+00	0.037714228	2.98E-02	0.00E+00	0	2.88E-02	0.235307815	0.00E+00	0	0.23769504	1.09E-01	0.084964221	1.74E-02	0.023965036	0	0.00E+00	EGFR_Wt
TCGA-97-A4M3-01	0.00E+00	0.014257964	0.072387028	0.0732	0	0.212701843	0.00E+00	0.084960373	1.19E-02	0.00E+00	0	5.53E-02	0.032101229	0.00E+00	0	0.30354602	0.00E+00	0.031761806	0.00E+00	0.1078466	0	0.00E+00	EGFR_Wt
TCGA-44-6774-01	1.00E-03	0	0.094134071	0.0311	0	0.084715856	1.23E-03	0.043499654	1.69E-02	2.99E-04	0	6.32E-03	0.073839524	1.32E-01	0.049392693	0.39672412	1.36E-02	0.002048593	0.00E+00	0.032710671	0	2.08E-02	EGFR_Wt
TCGA-75-6205-01	0.00E+00	0.022636302	0.007841457	0.0655	0	0.230772059	3.11E-03	0.018042022	5.69E-03	0.00E+00	0	0.00E+00	0.006383979	0.00E+00	0.106492532	0.4820669	1.06E-02	0	4.09E-02	0	0	0.00E+00	EGFR_Wt
TCGA-86-A456-01	1.88E-02	0.004163414	0.01704042	0.0379	0	0.127395664	0.00E+00	0.048838199	0.00E+00	2.23E-02	0	2.57E-02	0	1.37E-01	0.048667991	0.31857092	0.00E+00	0.018465188	0.00E+00	0.166623487	0	9.05E-03	EGFR_Wt
TCGA-L9-A8F4-01	4.80E-03	0.041104654	0.114722564	0.149	0	0.069852312	1.66E-02	0.070150026	6.89E-02	0.00E+00	0	6.24E-02	0	1.08E-01	0.083504655	0.19618984	9.95E-03	0	5.16E-03	0	0	0.00E+00	EGFR_Wt
TCGA-05-4424-01	2.88E-02	0	0.032442958	0.0933	0	0.133879453	0.00E+00	0.027416361	5.97E-03	0.00E+00	0	4.72E-03	0.029439826	9.47E-02	0.070360583	0.45227802	7.80E-04	0	1.65E-02	0	0	9.35E-03	EGFR_Wt
TCGA-49-4487-01	2.09E-02	0.094005067	0.066845626	0.15	0	0.15737699	0.00E+00	0.049659938	3.95E-02	9.31E-03	0	4.77E-02	0	1.59E-01	0.066590569	0.13855229	0.00E+00	0	0.00E+00	0	0	0.00E+00	EGFR_Wt
TCGA-55-6970-01	2.18E-02	0	0.093703325	0.0878	0	0.198652896	0.00E+00	0.055263462	0.00E+00	0.00E+00	0	3.04E-02	0.045294777	1.17E-02	0.053768985	0.30063739	0.00E+00	0.063839463	3.18E-02	0	0	5.35E-03	EGFR_Wt
TCGA-MN-A4N1-01	0.00E+00	0.030922187	0.016211186	0.0162	0	0.104077459	0.00E+00	0.060913442	2.73E-02	0.00E+00	0.009278826	1.85E-02	0.106093984	2.89E-01	0	0.24081114	0.00E+00	0.070996413	9.66E-03	0	0	0.00E+00	EGFR_Wt
TCGA-55-7727-01	4.98E-02	0.07143699	0.066473694	0.0369	0	0.159672043	3.73E-02	0.003924947	0.00E+00	6.97E-02	0	0.00E+00	0	0.00E+00	0.094823184	0.3645248	1.04E-02	0	3.39E-02	0	0	1.03E-03	EGFR_Wt
TCGA-J2-8194-01	1.55E-02	0	0.105604253	0	0	0.238020553	0.00E+00	0.036798546	4.97E-03	0.00E+00	0.055780247	0.00E+00	0.099810295	0.00E+00	0.038322056	0.29438146	2.97E-02	0.00575818	0.00E+00	0.038580967	0	3.68E-02	EGFR_Wt

TCGA-73-4666-01	1.71E-03	0	0.003908134	0.212	0	0.023810368	1.02E-01	0.047747974	1.76E-03	0.00E+00	0	4.06E-02	0.010982635	3.84E-02	0.109403759	0.39500271	0.00E+00	0	7.92E-03	0	0	4.31E-03	EGFR_Wt
TCGA-95-7567-01	0.00E+00	0.008062313	0.167088341	0.112	0	0.119180531	3.06E-02	0.041928319	4.97E-02	0.00E+00	0	5.29E-02	0.009277298	0.00E+00	0.07417366	0.31492019	7.41E-03	0.012447459	0.00E+00	0	0	3.01E-04	EGFR_Wt
TCGA-44-2656-01	6.65E-03	0	0.105046126	0.184	0	0.115359717	3.20E-03	0.040846272	1.98E-03	0.00E+00	0	2.87E-02	0.015999701	3.83E-02	0.10782866	0.3092465	1.64E-02	0	2.18E-02	0	0	4.56E-03	EGFR_Wt
TCGA-53-7626-01	0.00E+00	0.042103803	0.078913405	0.106	0	0.214623867	0.00E+00	0.053826876	6.67E-04	0.00E+00	0	3.87E-02	0.037885821	1.78E-02	0.067093228	0.26934552	1.80E-02	0	0.00E+00	0.044684712	0	1.07E-02	EGFR_Wt
TCGA-05-4250-01	1.86E-02	0	0.024902542	0.206	0	0.022615877	1.21E-01	0.054389136	0.00E+00	0.00E+00	0	3.26E-02	0.010402863	0.00E+00	0.063568784	0.38929888	0.00E+00	0	0.00E+00	0.050715708	0	5.88E-03	EGFR_Wt
TCGA-55-7227-01	2.27E-03	0.005202714	0.022192015	0.0525	0	0.247703265	0.00E+00	0.037343901	3.21E-02	0.00E+00	0	1.94E-02	0.059335407	1.12E-02	0.064103259	0.2345652	1.44E-01	0.019731212	4.79E-02	0	0	1.73E-04	EGFR_Wt
TCGA-05-4417-01	7.66E-03	0.028503125	0.041849388	0.107	0	0.179861544	6.46E-02	0.06027357	2.02E-02	0.00E+00	0	4.62E-02	0.009692888	6.67E-02	0.058970915	0.25290153	2.01E-02	0	2.80E-02	0	0	7.32E-03	EGFR_Wt
TCGA-MP-A4TD-01	3.93E-03	0.105684511	0.089910541	0.0674	0	0.175171522	0.00E+00	0.030782322	1.74E-02	0.00E+00	0	4.69E-02	0.00242874	2.10E-01	0.009618556	0.20092241	0.00E+00	0.009067139	3.05E-02	0	0	0.00E+00	EGFR_Wt
TCGA-50-5941-01	2.98E-02	0	0.052457419	0.138	0	0.111527719	0.00E+00	0.071060514	4.40E-02	0.00E+00	0	5.53E-02	0.018837729	1.79E-02	0.163257086	0.23240048	7.11E-03	0	4.65E-02	0	0	1.17E-02	EGFR_Wt
TCGA-44-6775-01	8.48E-03	0	0.016705943	0.0428	0	0.076046979	0.00E+00	0.038732805	6.18E-02	0.00E+00	0.005547874	0.00E+00	0.071814556	1.77E-01	0.032209469	0.34747575	7.54E-02	0.015149514	2.79E-02	0	0	2.78E-03	EGFR_Wt
TCGA-55-A490-01	0.00E+00	0.001900907	0.088456959	0.103	0	0.076300431	0.00E+00	0.015760382	8.00E-02	0.00E+00	0	2.32E-02	0.071411059	1.97E-01	0.022044762	0.28236054	3.40E-02	0	0.00E+00	0.004767889	0	0.00E+00	EGFR_Wt
TCGA-49-4488-01	2.64E-03	0.011831066	0.065727461	0.151	0	0.084580175	0.00E+00	0.046730919	5.00E-02	0.00E+00	0	2.47E-02	0.014398771	9.11E-02	0.088950171	0.28956563	6.38E-02	0	6.18E-03	0.008411858	0	0.00E+00	EGFR_Wt
TCGA-55-8089-01	1.39E-02	0.004434959	0.016877666	0.179	0	0.000146246	4.34E-02	0.064379141	0.00E+00	0.00E+00	0	1.09E-01	0.015439487	1.22E-01	0.186750553	0.2084031	1.18E-02	0	2.45E-02	0	0	0.00E+00	EGFR_Wt
TCGA-05-4384-01	2.00E-03	0.010045448	0.055385255	0.0449	0	0.086186367	0.00E+00	0.01835987	3.14E-03	0.00E+00	0.003864162	2.08E-02	0.022750749	1.23E-01	0.022610067	0.51863185	2.73E-02	0	0.00E+00	0.023718918	0	1.68E-02	EGFR_Wt
TCGA-50-6594-01	0.00E+00	0	0.114381649	0.0184	0	0.22057066	5.27E-03	0.010381862	2.55E-02	0.00E+00	0.036154746	1.87E-02	0.061999358	9.84E-02	0.019980913	0.30036307	2.24E-02	0.027895344	1.00E-02	0	0	9.57E-03	EGFR_Wt
TCGA-55-6979-01	0.00E+00	0.005805566	0.074009845	0.103	0	0.111256102	2.62E-02	0.055908577	3.27E-02	0.00E+00	0	4.24E-02	0.015132119	4.60E-02	0.117122948	0.35309014	9.66E-03	0	2.02E-03	0.002901816	0	3.04E-03	EGFR_Wt
TCGA-73-4659-01	9.96E-04	0	0.046695392	0.038	0	0.134804509	0.00E+00	0.003546627	0.00E+00	0.00E+00	0.045664988	0.00E+00	0.050932561	5.72E-03	0.00088703	0.56223603	1.95E-02	0	6.97E-02	0	0	2.13E-02	EGFR_Wt
TCGA-O1-A52J-01	5.82E-03	0	0.014929202	0.0422	0	0.070200093	0.00E+00	0.042384212	3.35E-02	0.00E+00	0.005334614	2.99E-02	0.28879212	0.00E+00	0.000624109	0.34865732	1.03E-02	0.043315652	6.40E-02	0	0	0.00E+00	EGFR_Wt
TCGA-44-7660-01	2.33E-02	0	0.065108998	0.0959	0	0.079950347	0.00E+00	0.139663471	1.86E-02	0.00E+00	0	6.19E-02	0	2.49E-02	0.116860063	0.37009756	0.00E+00	0	8.03E-06	0	0	3.72E-03	EGFR_Wt
TCGA-99-8032-01	0.00E+00	0.053281272	0.249114265	0.0675	0	0.14259553	0.00E+00	0.038284507	7.99E-02	0.00E+00	0	4.69E-02	0.009134435	6.86E-02	0.017690431	0.17835521	0.00E+00	0.002558584	4.01E-02	0	0	6.03E-03	EGFR_Wt
TCGA-53-7624-01	7.27E-02	0	0.058668608	0.0955	0	0.03928604	0.00E+00	0.072374364	1.55E-02	1.09E-02	0	6.87E-02	0	1.87E-01	0.198268088	0.12082047	0.00E+00	0.028840912	0.00E+00	0.02849544	0	3.37E-03	EGFR_Wt
TCGA-55-7816-01	9.64E-03	0	0	0.102	0	0.022743656	0.00E+00	0.017140198	4.71E-03	4.26E-03	0	2.69E-02	0.191170762	0.00E+00	0.082266843	0.51922997	0.00E+00	0	2.03E-02	0	0	0.00E+00	EGFR_Wt
TCGA-91-6830-01	1.18E-02	0	0.030462091	0.108	0	0.134691078	0.00E+00	0.034822748	5.03E-05	0.00E+00	0	4.67E-02	0.041246372	1.11E-01	0.115968321	0.20459573	0.00E+00	0.035558997	1.10E-01	0	0	1.50E-02	EGFR_Wt
TCGA-62-A470-01	2.20E-03	0.002249686	0.041608029	0.0666	0	0	0.00E+00	0.027484651	9.96E-03	0.00E+00	0.068514734	0.00E+00	0.02648	3.43E-02	0	0.66200672	2.10E-03	0.016145553	0.00E+00	0.036735604	0	3.61E-03	EGFR_Wt
TCGA-91-A4BC-01	3.55E-03	0	0.103206089	0.365	0	0	1.09E-01	0.063203244	1.16E-02	8.85E-02	0	3.44E-02	0	4.71E-02	0.039077475	0.13224791	0.00E+00	0	0.00E+00	0.002927159	0	0.00E+00	EGFR_Wt
TCGA-35-4122-01	4.59E-03	0	0.092802644	0.0949	0	0.053398886	3.52E-02	0.044095768	0.00E+00	6.25E-03	0	5.16E-02	0.059845074	4.07E-02	0.061605719	0.41200457	4.49E-03	0	0.00E+00	0.030187104	0	8.32E-03	EGFR_Wt
TCGA-86-7701-01	1.32E-02	0.029343483	0.190181117	0.0932	0	0.279697782	1.63E-02	0.074316584	2.97E-02	0.00E+00	0	1.87E-02	0.004845088	2.84E-02	0.033178955	0.16049507	0.00E+00	0.013200253	8.45E-03	0.001238878	0	5.55E-03	EGFR_Wt
TCGA-L4-A4E6-01	1.00E-02	0.015873988	0	0.0381	0	0.074621929	0.00E+00	0.033953435	1.68E-02	0.00E+00	0.035389883	0.00E+00	0.019873158	1.24E-01	0.031684849	0.55372804	5.69E-03	0	2.68E-02	0.011453053	0	2.29E-03	EGFR_Wt
TCGA-55-7815-01	4.67E-02	0	0.045944929	0.0215	0	0.263387544	4.92E-02	0	0.00E+00	7.53E-02	0	0.00E+00	0	3.40E-02	0.035612917	0.34938077	5.56E-02	0.002523992	7.40E-03	0	0	1.35E-02	EGFR_Wt
TCGA-44-8119-01	3.51E-04	0	0.128798312	0.0728	0	0.078517167	4.20E-03	0.039349709	2.23E-02	0.00E+00	0.016675335	4.38E-02	0.054012777	1.94E-01	0.061970467	0.25172033	0.00E+00	0	0.00E+00	0.030118556	0	9.20E-04	EGFR_Wt
TCGA-99-8033-01	5.07E-03	0	0.078852716	0.167	0	0.010214746	4.89E-02	0.134093015	0.00E+00	0.00E+00	0	1.11E-01	0.019769053	2.97E-02	0.06984675	0.12304217	0.00E+00	0.060360998	0.00E+00	0.127803757	0	1.51E-02	EGFR_Wt
TCGA-95-A4VP-01	9.91E-02	0.008178113	0.087924047	0.157	0	0.090874913	0.00E+00	0.139049506	4.01E-02	0.00E+00	0	2.98E-02	0.023454033	5.23E-02	0.054123168	0.20558271	0.00E+00	0.010618134	2.20E-03	0	0	0.00E+00	EGFR_Wt
TCGA-49-AARE-01	3.06E-02	0.021575467	0.122218494	0.0746	0	0.040319537	0.00E+00	0.105343876	5.04E-02	0.00E+00	0	2.65E-02	0	2.60E-01	0.100930976	0.14433855	0.00E+00	0	2.37E-02	0	0	0.00E+00	EGFR_Wt
TCGA-97-8175-01	0.00E+00	0.016837347	0.00410484	0.0124	0	0.101708982	0.00E+00	0.059744872	2.13E-02	0.00E+00	0	1.84E-02	0.034314257	4.85E-02	0.032989854	0.33831354	2.15E-01	0.023974377	3.41E-02	0	0	3.81E-02	EGFR_Wt
TCGA-05-4403-01	8.65E-03	0.002994186	0	0.0488	0	0.126034181	0.00E+00	0.049776723	3.35E-02	0.00E+00	0.002836027	2.32E-02	0.068902428	1.07E-01	0.057406984	0.40222072	2.03E-02	0	4.86E-02	0	0	0.00E+00	EGFR_Wt
TCGA-55-A4DG-01	3.38E-02	0.129124545	0.138537451	0.122	0	0.128783683	0.00E+00	0.065880836	0.00E+00	0.00E+00	0	3.00E-02	0.013715592	2.28E-02	0.056476173	0.21591094	0.00E+00	0	4.33E-02	0	0	0.00E+00	EGFR_Wt
TCGA-69-7973-01	5.12E-02	0	0.060337043	0.0896	0	0.216708449	0.00E+00	0.050624315	2.52E-02	0.00E+00	0	3.40E-02	0.029989622	6.47E-03	0.028796477	0.32919111	0.00E+00	0.016679327	5.56E-02	0	0	5.51E-03	EGFR_Wt
TCGA-55-8094-01	3.18E-02	0	0.07764615	0.0578	0	0	0.00E+00	0.185469025	0.00E+00	0.00E+00	0	3.88E-02	0	6.20E-02	0	0.21981055	0.00E+00	0.284946141	3.89E-02	0	0	2.84E-03	EGFR_Wt
TCGA-55-8510-01	8.36E-04	0.017101329	0	0.121	0	0.082412849	0.00E+00	0.072468503	5.37E-02	0.00E+00	0	4.93E-02	0.033042951	7.70E-02	0.097890791	0.30353903	2.17E-02	0	6.55E-02	0	0	4.96E-03	EGFR_Wt
TCGA-78-7145-01	6.11E-03	0	0.025031409	0	0	0.212124345	1.45E-02	0.021319297	2.53E-02	0.00E+00	0.006322055	0.00E+00	0.091939604	8.60E-02	0.018365218	0.25587887	5.93E-02	0.053041483	5.87E-02	0.055050465	0	1.10E-02	EGFR_Wt
TCGA-69-7980-01	1.15E-03	0.004247155	0.020413357	0.124	0	0.179194106	0.00E+00	0.08225725	2.62E-02	0.00E+00	0	2.45E-02	0.003838704	1.34E-02	0.107277077	0.38365751	0.00E+00	0	1.56E-02	0	0	1.47E-02	EGFR_Wt
TCGA-62-A471-01	3.54E-02	0	0.170268891	0.156	0	0.015701327	0.00E+00	0.203630359	1.35E-02	0.00E+00	0	1.15E-01	0.005219332	8.81E-02	0.033277317	0.08468805	0.00E+00	0.034123182	1.33E-02	0.011990154	0	1.99E-02	EGFR_Wt
TCGA-78-7633-01	4.29E-02	0	0.041285531	0.0252	0	0.217664006	0.00E+00	0.057243405	0.00E+00	0.00E+00	0	6.03E-02	0.118549055	0.00E+00	0	0.2997294	0.00E+00	0.112981684	2.41E-02	0	0	0.00E+00	EGFR_Wt
TCGA-64-1680-01	1.68E-02	0	0.100552486	0.055	0	0.163381739	0.00E+00	0.060399493	0.00E+00	1.51E-02	0	1.79E-02	0	0.00E+00	0.048865583	0.45369364	0.00E+00	0	6.64E-02	0	0	1.81E-03	EGFR_Wt
TCGA-64-1679-01	0.00E+00	0.009557889	0.096268743	0.0139	0	0.06275469	0.00E+00	0.00326024	1.51E-02	0.00E+00	0	9.22E-03	0	2.74E-01	0.037388089	0.25930909	1.40E-01	0.017007505	1.37E-02	0.039415823	0	9.53E-03	EGFR_Wt
TCGA-75-6203-01	0.00E+00	0	0.027478004	0.0645	0	0.139181969	0.00E+00	0.00473385	8.56E-05	0.00E+00	0	3.16E-02	0.022316791	8.97E-02	0.014623978	0.50268203	1.87E-02	0.009221501	6.62E-02	0	0	8.85E-03	EGFR_Wt
TCGA-97-7554-01	1.29E-03	0.013330805	0.168441741	0.0489	0	0.138573684	0.00E+00	0.033523723	2.06E-02	0.00E+00	0	1.40E-02	0.010286706	9.74E-02	0.062148101	0.35321592	0.00E+00	0.001610511	3.23E-02	0	0	4.36E-03	EGFR_Wt
TCGA-95-A4VK-01	5.62E-02	0.099406682	0.077981454	0.118	0	0.173337636	0.00E+00	0.098690586	4.00E-02	0.00E+00	0	4.60E-02	0.013193313	6.90E-02	0.070536615	0.11042554	0.00E+00	0.004271689	2.29E-02	0	0	0.00E+00	EGFR_Wt
TCGA-78-8655-01	1.31E-02	0	0.012658661	0.114	0	0.134196002	0.00E+00	0.042143495	1.72E-02	0.00E+00	0	1.83E-02	0.036992809	1.81E-01	0.102709141	0.24457227	1.41E-02	0	6.87E-02	0	0	1.11E-03	EGFR_Wt
TCGA-49-4486-01	3.65E-02	0	0.269184774	0.0628	0	0.155129576	0.00E+00	0.1029605	2.22E-03	0.00E+00	0	2.82E-02	0.013006379	0.00E+00	0.032307216	0.24852588	0.00E+00	0.036785137	0.00E+00	0.011346029	0	1.00E-03	EGFR_Wt
TCGA-55-8208-01	5.21E-03	0.057244878	0.0478716	0.0959	0	0.097501779	2.99E-03	0.052118414	3.51E-02	0.00E+00	0	5.69E-02	0.019240332	6.01E-02	0.1344571	0.27822748	1.01E-02	0	2.88E-02	0	0	1.82E-02	EGFR_Wt
TCGA-05-4415-01	4.44E-02	0	0.122863869	0.208	0	0.020497207	4.23E-02	0.063361001	0.00E+00	0.00E+00	0.033941253	5.48E-02	0.019591925	4.35E-02	0.126218695	0.17034288	0.00E+00	0.000300516	0.00E+00	0.037082905	0	1.27E-02	EGFR_Wt
TCGA-97-A4M5-01	6.67E-03	0.020925432	0.027560766	0.0661	0	0.119903408	0.00E+00	0.038776722	1.65E-02	0.00E+00	0.010652862	3.88E-02	0.025828082	7.96E-02	0.032259028	0.36053331	0.00E+00	0.03176867	1.11E-01	0	0	1.28E-02	EGFR_Wt
TCGA-MP-A4TH-01	2.25E-02	0.166778244	0.081827774	0.136	0	0.139700203	0.00E+00	0.071746962	6.38E-02	0.00E+00	0	5.11E-02	0.00886813	1.15E-02	0.019876902	0.17937516	1.27E-02	0.010641696	2.39E-02	0	0	0.00E+00	EGFR_Wt
TCGA-75-5125-01	2.06E-02	0	0	0.0746	0	0.106048517	7.36E-03	0.06707549	0.00E+00	0.00E+00	0	6.81E-02	0.030359013	0.00E+00	0.123652116	0.37557377	1.93E-02	0.022207804	6.75E-02	0	0	1.77E-02	EGFR_Wt
TCGA-MP-A4TA-01	1.52E-02	0	0.108171108	0.151	0	0.093921112	0.00E+00	0.069044832	4.80E-03	0.00E+00	0	1.10E-01	0.011428669	1.01E-01	0.062545699	0.25509866	0.00E+00	0	0.00E+00	0.016596509	0	1.13E-03	EGFR_Wt
TCGA-73-4662-01	0.00E+00	0.068529685	0.116784638	0.0817	0	0.17857489	0.00E+00	0.045951841	4.46E-02	0.00E+00	0	4.40E-02	0.123107002	1.26E-02	0.02347764	0.17175525	4.45E-02	0.028300416	1.29E-02	0.003404583	0	0.00E+00	EGFR_Wt
TCGA-95-7944-01	4.35E-03	0	0.005768132	0.245	0	0	1.28E-01	0.131688743	0.00E+00	0.00E+00	0	1.51E-01	0.007015308	6.17E-02	0.111477114	0.14215579	0.00E+00	0.00259006	0.00E+00	0.010027275	0	0.00E+00	EGFR_Wt
TCGA-86-8278-01	0.00E+00	0.013095839	0.121187441	0.064	0	0.178382487	0.00E+00	0.045607271	4.86E-03	0.00E+00	0	4.45E-02	0.025695079	3.34E-03	0.050719943	0.22350151	5.54E-02	0.063697013	1.01E-01	0	0	4.70E-03	EGFR_Wt
TCGA-55-7283-01	1.11E-02	0	0.00718948	0.102	0	0.253846919	0.00E+00	0.032799886	0.00E+00	0.00E+00	0	2.33E-02	0.041386427	3.08E-02	0.065068953	0.29719688	3.55E-02	0.00894078	8.95E-02	0	0	1.54E-03	EGFR_Wt
TCGA-55-7726-01	5.42E-03	0	0.139113728	0.0258	0	0.145253745	1.81E-02	0.005521362	0.00E+00	0.00E+00	0	5.05E-03	0.00698009	0.00E+00	0.076325539	0.24725582	2.43E-01	0	0.00E+00	0.07233336	0	9.72E-03	EGFR_Wt
TCGA-MP-A4SY-01	1.65E-02	0	0.021339007	0.0178	0	0.08662873	0.00E+00	0.01863113	2.56E-02	0.00E+00	0	3.32E-02	0.005280457	2.43E-01	0.048454028	0.32077651	9.30E-02	0	6.84E-02	0	0	1.02E-03	EGFR_Wt
TCGA-55-7995-01	1.46E-02	0.037386163	0	0.117	0	0.102123507	0.00E+00	0.056504816	4.81E-02	3.34E-02	0	4.60E-02	0.02823851	2.79E-01	0.114588676	0.10929021	4.97E-03	0	0.00E+00	0.008387615	0	0.00E+00	EGFR_Wt
TCGA-50-5935-01	0.00E+00	0.018862626	0.05713446	0.0671	0	0.284348922	1.65E-03	0.000783915	5.25E-02	0.00E+00	0	1.01E-02	0.041420063	1.02E-01	0.045442487	0.14976549	5.87E-02	0.058469578	5.16E-02	0	0	0.00E+00	EGFR_Wt
TCGA-L9-A7SV-01	3.99E-02	0	0.090780062	0.101	0	0.184234295	0.00E+00	0.1334693	0.00E+00	7.22E-03	0	4.41E-02	0.00201628	0.00E+00	0.098921576	0.29129666	0.00E+00	0	0.00E+00	0.007339672	0	0.00E+00	EGFR_Wt
TCGA-55-7725-01	8.26E-02	0	0.023841396	0.0724	0	0.168759222	8.27E-03	0.054345741	3.45E-03	3.62E-02	0	0.00E+00	0.007329678	4.81E-02	0.127965284	0.2115088	3.01E-02	0.009963703	1.12E-01	0	0	3.45E-03	EGFR_Wt
TCGA-95-7562-01	3.10E-02	0	0.035803258	0.0772	0	0.1328735	5.95E-03	0.029373615	4.82E-02	0.00E+00	0	9.30E-03	0.00644851	1.53E-01	0.148580357	0.29102057	0.00E+00	0	2.55E-02	0	0	6.00E-03	EGFR_Wt

TCGA-05-4398-01	3.60E-03	0.008826606	0.07741144	0.0631	0	0.164879303	0.00E+00	0.036256545	1.72E-02	0.00E+00	0	2.99E-02	0.057192867	2.24E-02	0.086430664	0.37790575	3.10E-02	0	0.00E+00	0.019084744	0	4.86E-03	EGFR_Wt
TCGA-55-6982-01	1.62E-02	0.029149634	0.045746449	0.0537	0	0.049041815	7.43E-03	0.034823441	1.24E-02	0.00E+00	0	0.00E+00	0.00527111	1.01E-01	0.180558914	0.41755115	3.23E-02	0.006388969	0.00E+00	0.0046934	0	3.78E-03	EGFR_Wt
TCGA-50-7109-01	5.33E-03	0.002137757	0.177660455	0.134	0	0.113028654	0.00E+00	0.065496956	1.89E-02	0.00E+00	0	4.89E-02	0.019279356	6.41E-02	0.117075839	0.20768307	0.00E+00	0	0.00E+00	0.023060544	0	3.63E-03	EGFR_Wt
TCGA-44-2657-01	6.86E-02	0.034616964	0.115686234	0.153	0	0.128007433	3.85E-03	0.061176542	1.56E-02	2.24E-02	0	4.05E-02	0	4.47E-03	0.073624739	0.21807248	9.27E-04	0	5.48E-02	0	0	4.26E-03	EGFR_Wt
TCGA-49-6742-01	4.97E-04	0	0.070262411	0.0909	0	0.136196115	0.00E+00	0.131447484	3.26E-03	0.00E+00	0	2.36E-02	0.044391502	0.00E+00	0.020377007	0.3571162	0.00E+00	0.079206737	3.24E-02	0	0	1.04E-02	EGFR_Wt
TCGA-NJ-A4YG-01	0.00E+00	0.020458056	0.034330775	0.0616	0	0.114436934	0.00E+00	0.092937666	3.80E-02	0.00E+00	0	3.83E-02	0.023998013	4.98E-02	0.043831314	0.31495781	9.68E-02	0.019583547	4.66E-02	0	0	4.40E-03	EGFR_Wt
TCGA-55-7281-01	0.00E+00	0.022993765	0.014487035	0.0622	0	0.145865574	0.00E+00	0.038656244	8.93E-03	0.00E+00	0	3.75E-02	0.075828583	0.00E+00	0.08869631	0.31175078	1.71E-01	0	2.23E-02	0	0	0.00E+00	EGFR_Wt
TCGA-05-4244-01	0.00E+00	0.042220555	0.006547029	0.0453	0	0.145816841	0.00E+00	0.056268756	7.23E-03	0.00E+00	0.002573353	1.40E-02	0.127183202	9.57E-02	0.021750397	0.30787751	5.15E-02	0.017695648	5.83E-02	0	0	1.05E-04	EGFR_Wt
TCGA-49-4514-01	7.37E-02	0	0.054907074	0.181	0	0.065037745	1.98E-02	0.148620929	2.48E-02	0.00E+00	0	1.03E-01	0.032418382	1.40E-02	0.072404057	0.17263832	4.27E-04	0.012241033	3.71E-03	0.016195552	0	4.87E-03	EGFR_Wt
TCGA-MP-A4T6-01	6.76E-03	0.040920095	0.039354669	0.0779	0	0.139157773	0.00E+00	0.123184211	4.98E-02	0.00E+00	0	4.06E-02	0.001950575	2.11E-01	0.05769904	0.1660941	1.05E-02	0.000562859	3.40E-02	0	0	0.00E+00	EGFR_Wt
TCGA-55-8203-01	0.00E+00	0.074722462	0.191375661	0.101	0	0.176025853	0.00E+00	0.080728166	5.01E-02	0.00E+00	0	2.30E-02	0.011652892	3.82E-02	0.068711985	0.1545924	9.02E-03	0.007963293	9.15E-03	0	0	3.72E-03	EGFR_Wt
TCGA-55-7573-01	6.22E-03	0.103193843	0.018821765	0.0663	0	0.246082443	0.00E+00	0.050237619	2.06E-02	0.00E+00	0	3.21E-02	0.023387072	0.00E+00	0.047809584	0.17628031	1.36E-01	0.00986074	5.99E-02	0	0	3.07E-03	EGFR_Wt
TCGA-49-6744-01	2.59E-03	0.014092475	0.088455071	0.0919	0	0.179098819	0.00E+00	0.047297186	3.74E-02	0.00E+00	0	4.09E-02	0.049789232	3.02E-02	0.035574274	0.16836496	8.99E-02	0.082155717	4.15E-02	0	0	8.62E-04	EGFR_Wt
TCGA-93-7347-01	9.76E-03	0.027687892	0.015465384	0.143	0	0.105308554	0.00E+00	0.059681031	6.33E-02	0.00E+00	0	2.03E-02	0	1.86E-01	0.04986092	0.26273524	0.00E+00	0	5.39E-02	0	0	3.29E-03	EGFR_Wt
TCGA-78-7542-01	3.15E-02	0	0.019942847	0.0312	0	0	3.89E-02	0.143803893	0.00E+00	1.21E-02	0.01565249	3.97E-02	0.004933153	4.55E-02	0.164119679	0.30601627	4.00E-02	0.07222199	3.44E-02	0	0	0.00E+00	EGFR_Wt
TCGA-69-7979-01	0.00E+00	0	0.119446447	0.0651	0	0.08802234	0.00E+00	0.053577412	6.14E-03	0.00E+00	0	6.05E-02	0.021338976	9.99E-02	0.061303117	0.32683709	0.00E+00	0.004825606	8.63E-02	0	0	6.73E-03	EGFR_Wt
TCGA-05-4395-01	1.96E-03	0	0.016213807	0.132	0	0.126351629	0.00E+00	0.032644752	9.53E-03	0.00E+00	0	1.03E-01	0.037199341	0.00E+00	0.043372409	0.39097082	1.67E-02	0.047436859	0.00E+00	0.037398217	0	5.26E-03	EGFR_Wt
TCGA-MP-A4SV-01	8.97E-05	0.023295545	0.068060862	0.158	0	0.151960348	1.16E-03	0.084139212	3.57E-02	0.00E+00	0	5.98E-02	0.017256057	3.19E-02	0.122125062	0.20105297	4.60E-03	0	4.04E-02	0	0	6.09E-04	EGFR_Wt
TCGA-MP-A4T4-01	2.44E-04	0	0.080827227	0.186	0	0.089483762	5.39E-02	0.074372614	1.06E-02	0.00E+00	0	4.70E-02	0.007792585	4.47E-02	0.117592306	0.25486609	1.43E-03	0	3.11E-02	0	0	3.33E-04	EGFR_Wt
TCGA-62-8398-01	1.18E-02	0	0.026150638	0.0651	0	0.199606076	0.00E+00	0.054345646	5.35E-02	0.00E+00	0.00937713	3.43E-02	0.052654753	8.75E-02	0.092978761	0.26292676	3.45E-02	0	8.83E-03	0	0	6.42E-03	EGFR_Wt
TCGA-44-8117-01	2.03E-02	0	0.034612911	0.0634	0	0.097058532	0.00E+00	0.052023204	5.48E-03	6.80E-03	0	1.06E-02	0	2.24E-01	0.074607875	0.3786674	0.00E+00	0	2.81E-02	0.004042384	0	3.92E-04	EGFR_Wt
TCGA-97-A4M2-01	0.00E+00	0.0443081	0.050688387	0.105	0	0.116800711	0.00E+00	0.083036916	1.90E-02	0.00E+00	0	3.86E-02	0.045137243	1.28E-01	0.027847886	0.24219159	1.52E-02	0.00574759	7.39E-02	0	0	4.64E-03	EGFR_Wt
TCGA-55-8507-01	0.00E+00	0.010388136	0.025055494	0.0799	0	0.121438581	0.00E+00	0.071960783	5.66E-03	0.00E+00	0	7.35E-02	0.044545415	1.52E-01	0.045138515	0.30402776	0.00E+00	0	2.19E-02	0.018073516	0	2.68E-02	EGFR_Wt
TCGA-44-7672-01	6.97E-03	0.036079735	0.094420346	0.0652	0	0.160960522	0.00E+00	0.04339982	6.21E-02	0.00E+00	0	6.61E-02	0.037665281	2.44E-02	0.039893008	0.23087221	4.89E-02	0.027885248	4.09E-02	0	0	1.42E-02	EGFR_Wt
TCGA-91-6831-01	0.00E+00	0.000260156	0.118830609	0.0872	0	0.116315025	0.00E+00	0.018350185	4.52E-02	0.00E+00	0	4.01E-02	0.025017846	1.63E-01	0.085729787	0.28192293	0.00E+00	0	2.13E-03	0	0	1.54E-02	EGFR_Wt
TCGA-78-7146-01	1.08E-02	0	0.03194578	0.0971	0	0.12269752	5.77E-02	0.034141954	0.00E+00	0.00E+00	0	4.15E-02	0.019798026	1.34E-01	0.058628957	0.25107277	0.00E+00	0.031313657	0.00E+00	0.107994848	0	1.46E-03	EGFR_Wt
TCGA-73-A9RS-01	3.12E-02	0	0.065581703	0.157	0	0.092819686	2.56E-02	0.134500506	3.28E-03	0.00E+00	0.010113511	8.15E-03	0.001834448	4.95E-02	0.071198765	0.28408578	0.00E+00	0	6.50E-02	0	0	0.00E+00	EGFR_Wt
TCGA-50-5044-01	0.00E+00	0.014851657	0.069227714	0.126	0	0.086401927	0.00E+00	0.053847767	4.79E-02	0.00E+00	0	9.24E-02	0.008622238	2.66E-02	0.034014963	0.29524627	1.61E-02	0.087198078	4.14E-02	0	0	0.00E+00	EGFR_Wt
TCGA-86-6562-01	0.00E+00	0.011159409	0.091986565	0.0222	0	0.136387204	0.00E+00	0.013880793	2.82E-02	0.00E+00	0.018635077	3.67E-05	0.003528894	1.58E-01	0.036349037	0.41267238	3.73E-03	0	2.46E-02	0.0144883	0	2.39E-02	EGFR_Wt
TCGA-05-4430-01	0.00E+00	0.017088747	0.01584978	0.0637	0	0.092299635	0.00E+00	0.034233486	3.10E-02	0.00E+00	0	3.66E-02	0.016173022	5.07E-02	0.04411137	0.43866427	7.22E-02	0.014741727	5.76E-02	0	0	1.51E-02	EGFR_Wt
TCGA-55-8615-01	9.55E-03	0	0.01176349	0.0266	0	0.026188538	0.00E+00	0.153616138	3.15E-02	0.00E+00	0.002568015	2.72E-02	0.031403019	3.93E-01	0	0.22053879	0.00E+00	0.058504854	7.50E-03	0	0	0.00E+00	EGFR_Wt
TCGA-55-8302-01	0.00E+00	0.024368163	0.053548236	0.126	0	0.061078515	0.00E+00	0.08979313	1.44E-02	0.00E+00	0	6.84E-02	0.065734972	6.17E-02	0.155232984	0.26505349	3.71E-04	0.002087474	1.12E-03	0.011516352	0	0.00E+00	EGFR_Wt
TCGA-44-6777-01	5.78E-03	0.007893414	0.054744664	0.0396	0	0.10303861	0.00E+00	0.015878128	1.38E-02	0.00E+00	0	3.65E-02	0.077943042	1.75E-02	0.020828454	0.46578468	5.20E-02	0.013213422	6.46E-02	0	0	1.09E-02	EGFR_Wt
TCGA-67-3774-01	0.00E+00	0.025640843	0.05790175	0.0729	0	0.280033556	0.00E+00	0.032270078	1.05E-03	0.00E+00	0	3.07E-02	0.014875903	3.72E-02	0.081458107	0.31125616	0.00E+00	0.0043794	4.53E-02	0	0	5.00E-03	EGFR_Wt
TCGA-55-8512-01	6.41E-03	0	0.049332592	0.146	0	0.101105018	0.00E+00	0.075146808	0.00E+00	0.00E+00	0.011813877	7.77E-02	0.115627896	4.33E-02	0	0.21878135	0.00E+00	0.057311298	1.13E-02	0.030004752	0	5.59E-02	EGFR_Wt
TCGA-49-6767-01	1.60E-02	0	0	0.1	0	0.053376317	2.91E-02	0.043965574	2.08E-02	0.00E+00	0	1.18E-01	0	3.40E-01	0.081069225	0.18246716	0.00E+00	0	1.45E-02	0	0	0.00E+00	EGFR_Wt
TCGA-50-8459-01	2.59E-03	0.042528327	0.030275369	0.0697	0	0.072877912	1.50E-03	0.029532269	4.70E-02	0.00E+00	0	2.61E-02	0.121076128	9.32E-02	0.016029466	0.33054264	5.74E-02	0	5.92E-02	0	0	3.60E-04	EGFR_Wt
TCGA-50-5939-01	0.00E+00	0.060210468	0.145613617	0.0326	0	0.119011584	6.72E-03	0.030595376	1.05E-02	0.00E+00	0	4.78E-02	0.02891678	1.16E-01	0.03811927	0.31551318	0.00E+00	0	4.52E-03	0.027226788	0	1.61E-02	EGFR_Wt
TCGA-05-5429-01	0.00E+00	0.024086198	0.1311865	0.0476	0	0.28938162	0.00E+00	0.011228731	0.00E+00	2.73E-03	0	2.31E-02	0.008636644	0.00E+00	0.032380704	0.34849647	2.62E-02	0	0.00E+00	0.034856418	0	2.01E-02	EGFR_Wt
TCGA-55-8513-01	9.53E-03	0.069273757	0.036520676	0.0231	0	0.084960971	0.00E+00	0.021519482	1.05E-02	0.00E+00	0.00652038	1.52E-02	0.022533254	9.54E-02	0.013396499	0.49052395	3.79E-02	0.009917258	4.92E-02	0	0	4.01E-03	EGFR_Wt
TCGA-64-5779-01	0.00E+00	0.043466222	0.377418972	0.125	0	0.202084809	3.33E-02	0.024096349	1.01E-02	4.20E-03	0	0.00E+00	0	5.40E-02	0.065777387	0.05893916	0.00E+00	0	0.00E+00	0.002144598	0	0.00E+00	EGFR_Wt
TCGA-44-6148-01	0.00E+00	0.074976021	0.145229353	0.0502	0	0.094494289	0.00E+00	0.027899719	2.19E-03	0.00E+00	0	1.06E-02	0.07116816	8.89E-03	0.003143178	0.31682127	0.00E+00	0.0126918	1.77E-01	0	0	4.26E-03	EGFR_Wt
TCGA-75-5122-01	0.00E+00	0	0.016616221	0.199	0	0.083893657	0.00E+00	0.048866823	0.00E+00	0.00E+00	0	3.54E-02	0.033021894	0.00E+00	0.038473613	0.42396153	3.73E-02	0	0.00E+00	0.082406545	0	1.26E-03	EGFR_Wt
TCGA-64-5781-01	0.00E+00	0.001751644	0.090256855	0.266	0	0	8.79E-02	0.119125164	4.37E-03	0.00E+00	0	1.98E-01	0	4.89E-02	0.039171061	0.08578256	0.00E+00	0.01158923	0.00E+00	0.047948727	0	0.00E+00	EGFR_Wt
TCGA-50-6593-01	0.00E+00	0.026314416	0.028807917	0.0325	0	0.092146851	0.00E+00	0.026225136	2.29E-02	0.00E+00	0	1.16E-02	0.095532616	1.72E-01	0.013277933	0.30527737	7.35E-02	0.01303784	7.63E-02	0	0	1.02E-02	EGFR_Wt
TCGA-93-8067-01	1.07E-03	0	0.067755516	0.0674	0	0.128423281	0.00E+00	0.079322026	2.47E-02	0.00E+00	0	9.19E-02	0.014984944	3.59E-02	0.088898758	0.32690813	4.22E-02	0.020414377	0.00E+00	0.010089809	0	0.00E+00	EGFR_Wt
TCGA-50-5045-01	1.04E-02	0.032448516	0.117831088	0.0785	0	0.142394327	0.00E+00	0.062571203	2.18E-02	3.27E-02	0	1.91E-02	0.017228295	0.00E+00	0.057716389	0.31268612	5.62E-02	0.003760429	3.33E-02	0	0	1.29E-03	EGFR_Wt
TCGA-93-A4JQ-01	1.49E-02	0.169167193	0	0.0543	0	0.173692497	0.00E+00	0.094754104	4.91E-02	0.00E+00	0.011039789	2.64E-02	0	1.51E-01	0.092289569	0.15607293	0.00E+00	0	2.33E-03	0.002480077	0	2.55E-03	EGFR_Wt
TCGA-35-5375-01	1.04E-02	0.030918235	0.06322159	0.141	0	0.043404677	3.34E-03	0.106969629	0.00E+00	1.27E-01	0	3.80E-02	0	2.06E-02	0.147219867	0.18745475	2.68E-02	0	2.41E-02	0.028541132	0	6.08E-04	EGFR_Wt
TCGA-55-A491-01	2.32E-02	0.013092801	0.131471918	0.0927	0	0.104255409	0.00E+00	0	4.58E-02	0.00E+00	0	7.58E-02	0.033768791	1.75E-01	0.118883703	0.17606703	1.08E-03	0	8.53E-03	0	0	0.00E+00	EGFR_Wt
TCGA-55-8091-01	0.00E+00	0	0.041525059	0.0278	0	0.175080864	0.00E+00	0.012698371	1.73E-02	0.00E+00	0	6.00E-02	0.024793055	0.00E+00	0.08052161	0.3608879	8.41E-02	0.003293543	1.03E-01	0	0	9.20E-03	EGFR_Wt
TCGA-NJ-A4YF-01	1.48E-02	0	0.058393867	0.0567	0	0.087938868	0.00E+00	0.126252763	4.84E-02	0.00E+00	0	2.92E-02	0	3.14E-01	0.047954283	0.17075179	0.00E+00	0	0.00E+00	0.04446787	0	1.45E-03	EGFR_Wt
TCGA-62-A46V-01	3.79E-02	0	0.091911488	0.00156	0	0.11442106	0.00E+00	0.006047188	0.00E+00	0.00E+00	0	2.98E-02	0.051799983	1.79E-02	0	0.37656888	8.74E-02	0.092404874	9.23E-02	0	0	0.00E+00	EGFR_Wt
TCGA-86-8671-01	1.43E-02	0.039721505	0.002303618	0.103	0	0.217239585	0.00E+00	0.039999173	5.50E-02	0.00E+00	0.010990507	1.75E-02	0.003692819	1.64E-01	0.129891435	0.15503659	3.51E-03	0	4.40E-02	0	0	0.00E+00	EGFR_Wt
TCGA-55-8619-01	3.20E-02	0.040024419	0.063006265	0.157	0	0.075356224	0.00E+00	0.075104644	3.08E-02	0.00E+00	0	5.29E-02	0.031503414	8.77E-02	0.024505812	0.24789261	2.59E-03	0	6.01E-02	0	0	1.91E-02	EGFR_Wt
TCGA-67-6215-01	0.00E+00	0.020736691	0.003798191	0.0231	0	0.128699204	0.00E+00	0.004648232	5.54E-03	0.00E+00	0	1.83E-02	0.009295404	1.02E-01	0.022787354	0.4205336	8.11E-02	0	1.34E-01	0	0	2.56E-02	EGFR_Wt
TCGA-78-7220-01	1.94E-02	0	0.062652599	0.162	0	0.121527716	0.00E+00	0.129084455	0.00E+00	0.00E+00	0	8.03E-02	0.007953163	5.02E-02	0.058907883	0.26707271	0.00E+00	0	3.60E-02	0	0	5.27E-03	EGFR_Wt
TCGA-62-A46O-01	1.11E-01	0	0.39889092	0.0366	0	0	0.00E+00	0.067660868	0.00E+00	1.62E-02	0	4.93E-02	0	8.32E-02	0.001814904	0.08267465	0.00E+00	0.136431494	1.62E-02	0	0	0.00E+00	EGFR_Wt
TCGA-86-A4P8-01	1.85E-03	0.01720929	0	0.0993	0	0.251408056	0.00E+00	0.019014649	1.10E-04	0.00E+00	0	4.49E-02	0.025528544	9.24E-02	0.046826164	0.3492452	3.66E-04	0	5.17E-02	0	0	1.95E-04	EGFR_Wt
TCGA-91-6828-01	5.85E-04	0.007274348	0.047401584	0.0961	0	0.246727538	1.32E-02	0.01426709	0.00E+00	0.00E+00	0	6.61E-03	0.037514323	0.00E+00	0.072024651	0.40475022	3.09E-02	0	3.65E-03	0.003699972	0	1.54E-02	EGFR_Wt
TCGA-97-7552-01	1.46E-03	0.093175299	0.073756012	0.254	0	0.122385342	0.00E+00	0.03019481	1.74E-02	0.00E+00	0	5.84E-02	0.019653247	4.29E-02	0.106125866	0.1346484	2.03E-02	0	2.51E-02	0	0	0.00E+00	EGFR_Wt
TCGA-62-A46S-01	0.00E+00	0	0.167274881	0.0967	0	0.209999064	0.00E+00	0.010662796	2.07E-02	0.00E+00	0.004392838	1.52E-02	0.044556135	4.14E-02	0.078343382	0.27716144	2.02E-02	0	1.34E-02	0	0	0.00E+00	EGFR_Wt
TCGA-78-7153-01	3.11E-02	0.011433277	0.14203516	0.139	0	0.218088888	0.00E+00	0.058607778	2.91E-02	0.00E+00	0	8.71E-02	0.014936912	0.00E+00	0.025361905	0.17612227	0.00E+00	0.009307788	5.82E-02	0	0	0.00E+00	EGFR_Wt
TCGA-71-6725-01	0.00E+00	0.003907459	0.015718131	0.023	0	0.100483363	0.00E+00	0.010498283	6.37E-03	0.00E+00	0	6.00E-02	0.040310901	0.00E+00	0.001614464	0.60110846	1.70E-02	0.036689472	6.45E-02	0	0	1.88E-02	EGFR_Wt
TCGA-MP-A4TI-01	2.03E-03	0.002802725	0.005454999	0.0812	0	0.151164633	0.00E+00	0.055363974	3.55E-02	0.00E+00	0	4.95E-02	0.025737951	1.65E-01	0.127198913	0.27495484	0.00E+00	0.0039424	2.03E-02	0	0	0.00E+00	EGFR_Wt
TCGA-53-7813-01	0.00E+00	0.020628674	0.380321798	0.189	0	0.053496982	3.28E-02	0.067489834	2.51E-02	0.00E+00	0	5.16E-02	0.009609197	3.13E-02	0.032179211	0.05906982	0.00E+00	0.034278982	0.00E+00	0.013371982	0	0.00E+00	EGFR_Wt
TCGA-38-4630-01	2.03E-02	0	0.010040173	0.101	0	0.191603763	0.00E+00	0.037620722	2.96E-02	0.00E+00	0	4.25E-02	0.010805109	1.05E-01	0.091814954	0.32089747	6.89E-05	0.015028865	0.00E+00	0.023942467	0	0.00E+00	EGFR_Wt
TCGA-97-A4M0-01	4.80E-04	0.020635162	0	0.118	0	0.205180718	0.00E+00	0.049113121	5.37E-02	0.00E+00	0	7.35E-02	0.082111982	2.55E-04	0.051465346	0.18936647	4.82E-02	0.017013312	9.06E-02	0	0	0.00E+00	EGFR_Wt
TCGA-05-4420-01	1.42E-02	0	0.129683589	0.103	0	0.095804747	0.00E+00	0.043441761	1.40E-02	0.00E+00	0.018973789	9.81E-03	0.006950026	4.94E-02	0.004571924	0.32841288	7.33E-03	0.021996566	0.00E+00	0.029697928	0	1.23E-01	EGFR_Wt
TCGA-38-4632-01	0.00E+00	0.021921434	0.036029006	0.0303	0	0.068807447	7.23E-02	0.07063329	0.00E+00	5.03E-02	0.007461031	0.00E+00	0.02833283	0.00E+00	0.033250764	0.36102835	1.11E-01	0.0387553	0.00E+00	0.041026399	0	2.85E-02	EGFR_Wt

TCGA-44-6776-01	8.27E-02	0	0.109858911	0.1	0	0.161182705	0.00E+00	0.063317816	0.00E+00	0.00E+00	0	5.80E-02	0.034097981	0.00E+00	0.026048487	0.20724752	5.01E-02	0.041117967	6.44E-02	0	0	1.48E-03	EGFR_Wt
TCGA-50-6597-01	0.00E+00	0.051528915	0.168567926	0.0551	0	0.153972599	0.00E+00	0.014972665	1.44E-01	7.90E-02	0	3.16E-02	0.000791065	0.00E+00	0.077704028	0.15616302	2.39E-02	0.000143774	4.20E-02	0	0	0.00E+00	EGFR_Wt
TCGA-44-6778-01	2.70E-02	0.016428433	0.024391695	0.0731	0	0.196684645	7.99E-03	0.064962829	0.00E+00	5.93E-03	0	3.69E-02	0.006194133	8.98E-02	0.082826755	0.2908722	8.81E-04	0	0.00E+00	0.070573278	0	5.37E-03	EGFR_Wt
TCGA-62-A472-01	2.88E-02	0	0.000778396	0.126	0	0.192432949	0.00E+00	0.077686608	1.80E-02	0.00E+00	0	8.16E-02	0.015549883	1.93E-02	0.06400875	0.21553959	4.70E-02	0	1.10E-01	0	0	3.92E-03	EGFR_Wt
TCGA-55-A492-01	0.00E+00	0.011640235	0.214804299	0.113	0	0.095365257	0.00E+00	0.098479363	3.08E-02	0.00E+00	0	5.46E-02	0.033422539	8.90E-03	0.017289943	0.22675296	0.00E+00	0.043906898	5.11E-02	0	0	0.00E+00	EGFR_Wt
TCGA-69-7764-01	3.58E-02	0	0.186370288	0.147	0	0.208277214	0.00E+00	0.068284501	0.00E+00	2.19E-02	0	3.91E-02	0	0.00E+00	0.077864485	0.1554572	3.15E-03	0.006405862	0.00E+00	0.050179826	0	0.00E+00	EGFR_Wt
TCGA-78-7150-01	7.13E-02	0	0.192400434	0.0749	0	0.084822298	0.00E+00	0.067995853	2.14E-02	0.00E+00	0	7.32E-02	0.000883563	1.02E-01	0.050618176	0.18075285	0.00E+00	0.034776814	0.00E+00	0.041345125	0	3.35E-03	EGFR_Wt
TCGA-80-5611-01	0.00E+00	0.033906623	0	0.0382	0	0.041053193	0.00E+00	0.050185922	3.47E-02	0.00E+00	0	3.13E-02	0.162165158	2.55E-01	0.071018106	0.23940906	8.08E-03	0	0.00E+00	0.035486779	0	0.00E+00	EGFR_Wt
TCGA-86-8585-01	8.35E-03	0.04668617	0	0.142	0	0.052045625	0.00E+00	0.086155859	5.96E-02	7.22E-03	0	5.99E-02	0.040456471	2.49E-01	0.083808347	0.16025549	0.00E+00	0	4.20E-03	0	0	0.00E+00	EGFR_Wt
TCGA-44-A47A-01	5.60E-03	0.018418474	0	0.0454	0	0.114848376	0.00E+00	0.052170762	7.85E-02	0.00E+00	0.007086214	2.88E-02	0	7.43E-02	0.021973921	0.27270735	1.88E-01	0.046224631	4.54E-02	0	0	0.00E+00	EGFR_Wt
TCGA-50-5930-01	0.00E+00	0.05142467	0.200434241	0.144	0	0.080975875	0.00E+00	0.064920722	7.37E-03	0.00E+00	0	8.71E-03	0.002431721	1.54E-01	0.059355658	0.22271658	0.00E+00	0	3.50E-03	0	0	0.00E+00	EGFR_Wt
TCGA-49-AAR0-01	5.16E-02	0	0.066484472	0.0799	0	0.038918821	0.00E+00	0.186585711	4.14E-02	9.67E-03	0	4.99E-02	0.0064848	6.67E-02	0.060267381	0.31850897	3.12E-03	0	2.01E-02	0	0	3.74E-04	EGFR_Wt
TCGA-49-AARQ-01	1.80E-02	0	0	0.194	0	0.01373999	0.00E+00	0.15868531	5.91E-02	0.00E+00	0.004719687	3.88E-02	0.018964139	9.18E-02	0.200682932	0.17796096	0.00E+00	0	2.34E-02	0	0	0.00E+00	EGFR_Wt
TCGA-73-4677-01	0.00E+00	0.027318119	0.13975455	0.109	0	0.234958415	0.00E+00	0.013403726	8.49E-03	0.00E+00	0	3.98E-02	0.030286271	0.00E+00	0.030608084	0.31093759	3.02E-02	0.007301186	1.08E-02	0.002365102	0	5.01E-03	EGFR_Wt
TCGA-J2-A4AE-01	2.55E-02	0.06549606	0.149662059	0.0909	0	0.112118203	0.00E+00	0.095746869	3.12E-03	0.00E+00	0	4.84E-02	0.008304947	1.01E-02	0.053103741	0.25681044	5.91E-03	0.01498387	0.00E+00	0.049842133	0	1.01E-02	EGFR_Wt
TCGA-50-5051-01	3.04E-02	0.046177584	0.127138149	0.104	0	0.035099056	0.00E+00	0.078318734	6.92E-02	0.00E+00	0	4.78E-02	0.008082806	1.31E-01	0	0.1789203	2.49E-02	0.050750536	5.47E-02	0.013446198	0	0.00E+00	EGFR_Wt
TCGA-91-6840-01	5.72E-02	0	0.009519771	0.119	0	0.161624329	0.00E+00	0.044036358	1.01E-02	0.00E+00	0	5.17E-02	0.03463188	7.32E-02	0.176250089	0.22348642	3.00E-03	0	3.64E-02	0	0	0.00E+00	EGFR_Wt
TCGA-55-1594-01	3.10E-02	0	0.092736272	0.0841	0	0.166999209	0.00E+00	0.052642032	5.42E-03	0.00E+00	0	4.89E-02	0.005255551	1.56E-02	0.119795714	0.33591589	8.24E-03	0	7.11E-03	0	0	2.63E-02	EGFR_Wt
TCGA-55-5899-01	1.45E-02	0	0.044626982	0.0278	0	0.069335886	0.00E+00	0.045727986	1.32E-02	0.00E+00	0.007699607	5.41E-02	0	2.74E-01	0.075263433	0.34888372	2.53E-03	0	2.01E-02	0	0	2.10E-03	EGFR_Wt
TCGA-95-7948-01	0.00E+00	0.042456565	0.117454086	0.0849	0	0.099890999	0.00E+00	0.040174989	0.00E+00	1.47E-02	0	4.43E-02	0.047294285	0.00E+00	0.018442506	0.35721158	2.20E-02	0.027144358	8.41E-02	0	0	0.00E+00	EGFR_Wt
TCGA-L9-A444-01	1.15E-02	0.004504598	0.105209242	0.23	0	0.145268395	5.33E-03	0.109175858	1.46E-03	0.00E+00	0	5.00E-02	0.008054695	9.16E-03	0.16206014	0.14568819	2.22E-03	0	1.08E-02	0	0	0.00E+00	EGFR_Wt
TCGA-69-8254-01	0.00E+00	0.0542965	0.137270257	0.076	0	0.174465612	0.00E+00	0.077697956	5.79E-03	9.76E-03	0	1.57E-02	0.018204761	0.00E+00	0.08662666	0.27809922	1.71E-02	0.007662913	8.78E-04	0.031678971	0	8.76E-03	EGFR_Wt
TCGA-78-7167-01	3.08E-03	0	0.147395771	0.112	0	0.134590751	0.00E+00	0.084622545	8.26E-03	0.00E+00	0	8.26E-02	0.087028032	1.86E-02	0.049614831	0.18730894	0.00E+00	0.002375691	8.25E-02	0	0	0.00E+00	EGFR_Wt
TCGA-49-4506-01	7.01E-03	0.018609307	0.066945802	0.303	0	0.059689195	7.95E-03	0.089772674	1.40E-02	0.00E+00	0	8.22E-02	0.010188262	1.85E-02	0.072487588	0.21252057	1.12E-02	0	0.00E+00	0.022642948	0	3.65E-03	EGFR_Wt
TCGA-95-8039-01	7.36E-03	0.026583766	0	0.0424	0	0.254677834	0.00E+00	0.032145556	4.15E-02	0.00E+00	0	2.24E-02	0.030925276	0.00E+00	0.031071883	0.30831619	9.85E-02	0.039818007	6.04E-02	0	0	3.77E-03	EGFR_Wt
TCGA-75-6214-01	0.00E+00	0	0.082052622	0.0849	0	0.0772987	0.00E+00	0.066148508	3.50E-02	0.00E+00	0.006214721	6.57E-03	0	2.19E-01	0.03865477	0.32697984	7.27E-03	0.00538215	0.00E+00	0.024220384	0	2.06E-02	EGFR_Wt
TCGA-55-7903-01	3.63E-02	0	0.009710881	0.143	0	0.083548246	1.81E-02	0.093342899	5.11E-03	0.00E+00	0.004262214	3.69E-02	0.038080769	2.99E-02	0.126526492	0.25729458	3.30E-02	0.04206538	0.00E+00	0.041305653	0	1.32E-03	EGFR_Wt
TCGA-38-4631-01	5.70E-02	0	0.19880247	0.0939	0	0.094820212	0.00E+00	0.051965239	1.48E-02	2.63E-04	0	6.57E-02	0.016372455	9.29E-02	0.083431673	0.14354306	0.00E+00	0	0.00E+00	0.031249612	0	5.53E-02	EGFR_Wt
TCGA-97-7547-01	3.99E-02	0	0.087802061	0.0674	0	0.175190027	0.00E+00	0.045486608	5.81E-02	0.00E+00	0	3.53E-02	0.055168664	2.20E-02	0.042460876	0.29150629	0.00E+00	0	7.96E-02	0	0	0.00E+00	EGFR_Wt
TCGA-69-7974-01	7.06E-03	0.010506102	0.010832592	0.0675	0	0.117168071	0.00E+00	0.04633571	0.00E+00	0.00E+00	0	5.48E-02	0.038703333	2.88E-02	0.040540505	0.52156991	2.06E-02	0.004772939	5.05E-03	0.022165956	0	3.61E-03	EGFR_Wt
TCGA-55-8514-01	0.00E+00	0.052522016	0.059676167	0.0283	0	0.107186952	0.00E+00	0.079163613	1.85E-03	0.00E+00	0.021831689	6.94E-03	0.053039074	4.32E-02	0.057749984	0.44172324	0.00E+00	0.008167481	3.44E-02	0	0	4.20E-03	EGFR_Wt
TCGA-44-A479-01	8.14E-03	0.016673249	0.157956591	0.107	0	0.162675645	1.66E-02	0.052891516	2.65E-02	2.04E-02	0	1.95E-02	0.000171694	4.22E-02	0.132040873	0.22685648	0.00E+00	0	1.09E-02	0	0	0.00E+00	EGFR_Wt
TCGA-55-7576-01	1.29E-02	0.023869747	0	0.122	0	0.092259935	0.00E+00	0.030050005	5.16E-02	0.00E+00	0	4.53E-02	0.050161221	3.80E-02	0.058984833	0.36947945	2.18E-02	0.01639709	6.35E-02	0	0	3.65E-03	EGFR_Wt
TCGA-86-7954-01	1.78E-02	0	0	0.0729	0	0.191213983	0.00E+00	0.039771393	3.61E-02	0.00E+00	0	2.68E-02	0.014407507	0.00E+00	0.191833128	0.33887289	0.00E+00	0.014938354	5.34E-02	0	0	1.97E-03	EGFR_Wt
TCGA-91-8496-01	2.74E-03	0.001653314	0	0.0674	0	0.120664015	0.00E+00	0.002239366	1.62E-02	0.00E+00	0.029378087	8.47E-03	0.112811904	5.01E-02	0.018190511	0.37238191	5.55E-02	0	1.34E-01	0	0	8.30E-03	EGFR_Wt
TCGA-55-6984-01	3.07E-02	0.094735967	0.073756921	0.0964	0	0.236313715	0.00E+00	0.024233917	9.71E-02	0.00E+00	0	6.05E-02	0.033364899	4.09E-02	0.001196479	0.09416731	0.00E+00	0.034146524	0.00E+00	0.052176916	0	3.04E-02	EGFR_Wt
TCGA-91-7771-01	1.73E-02	0	0.043372926	0.113	0	0.304149659	0.00E+00	0.035959603	0.00E+00	0.00E+00	0	3.20E-02	0.019361812	1.26E-02	0.091672115	0.31946013	2.49E-04	0	9.51E-03	0	0	8.78E-04	EGFR_Wt
TCGA-67-3773-01	1.60E-02	0	0	0.0868	0	0.113124827	0.00E+00	0.090672621	0.00E+00	1.13E-02	0	8.58E-02	0.010113163	0.00E+00	0.068168775	0.31213859	8.82E-02	0.002685632	1.07E-01	0	0	8.10E-03	EGFR_Wt
TCGA-99-8028-01	0.00E+00	0.027976418	0.103355833	0.15	0	0.136869104	0.00E+00	0.052571305	5.42E-02	0.00E+00	0	3.01E-02	0.002201537	9.44E-02	0.094051653	0.25186672	0.00E+00	0	2.83E-03	0	0	0.00E+00	EGFR_Wt
TCGA-75-7030-01	1.67E-02	0.04574387	0.176908388	0.112	0	0.241300929	0.00E+00	0.022368127	1.06E-02	0.00E+00	0	2.92E-02	0.013742968	2.12E-02	0.05196369	0.18108704	3.32E-02	0.008961524	3.35E-02	0	0	1.38E-03	EGFR_Wt
TCGA-49-AARR-01	5.59E-02	0.026869163	0.086375064	0.116	0	0.060382948	0.00E+00	0.090915543	2.36E-02	0.00E+00	0	6.09E-02	0.009131229	2.56E-02	0.014237767	0.28203315	0.00E+00	0.0191025	1.12E-01	0	0	1.67E-02	EGFR_Wt
TCGA-05-4418-01	2.72E-03	0.015839788	0.119974037	0.113	0	0.141943664	0.00E+00	0.061790068	5.88E-02	0.00E+00	0	2.76E-02	0.022460025	2.88E-02	0.103812068	0.2979166	0.00E+00	0	9.18E-04	0	0	4.79E-03	EGFR_Wt
TCGA-86-7714-01	3.44E-04	0	0.007261992	0.0274	0	0.144764532	0.00E+00	0.000954853	3.91E-03	0.00E+00	0.00210131	9.44E-03	0.013525808	0.00E+00	0.010226155	0.24863251	3.54E-01	0.037661558	1.26E-01	0	0	1.39E-02	EGFR_Wt
TCGA-44-6779-01	0.00E+00	0.004717012	0.007929189	0.197	0	0.094253902	1.87E-02	0.050524158	2.71E-02	0.00E+00	0.005843907	3.36E-02	0.004658418	3.82E-02	0.170099327	0.32401787	0.00E+00	0	1.91E-02	0	0	3.76E-03	EGFR_Wt
TCGA-69-8253-01	1.94E-03	0.061843787	0.225654535	0.0686	0	0.122889539	0.00E+00	0.060482949	1.74E-02	0.00E+00	0	5.03E-02	0.019560037	1.96E-02	0	0.2130184	3.89E-02	0.053297193	3.48E-02	0	0	1.17E-02	EGFR_Wt
TCGA-50-6673-01	4.03E-02	0	0.16398089	0.0341	0	0.128359142	0.00E+00	0.043496701	7.65E-03	0.00E+00	0	4.25E-02	0.035299084	1.01E-01	0.019687578	0.26960899	4.47E-02	0.022281344	0.00E+00	0.042983836	0	4.41E-03	EGFR_Wt
TCGA-MP-A4T7-01	8.22E-03	0.011679197	0.039058253	0.0641	0	0.109919588	0.00E+00	0.110755129	9.50E-03	0.00E+00	0	5.69E-02	0.019246876	8.59E-02	0.01566892	0.35233329	0.00E+00	0.101619489	0.00E+00	0.007397607	0	7.73E-03	EGFR_Wt
TCGA-78-7540-01	4.76E-03	0.034033441	0	0.0557	0	0.063671883	0.00E+00	0.034274248	4.87E-02	0.00E+00	0	3.92E-02	0.000608928	2.29E-01	0.032069212	0.3637159	6.49E-04	0.008954078	8.44E-02	0	0	0.00E+00	EGFR_Wt
TCGA-64-5774-01	9.85E-03	0	0.238037765	0.0547	0	0.178536895	0.00E+00	0.05172381	8.22E-02	0.00E+00	0	3.68E-02	0	6.26E-02	0	0.19822181	0.00E+00	0.008927251	0.00E+00	0.062387105	0	1.60E-02	EGFR_Wt
TCGA-97-7937-01	1.52E-02	0	0.187506092	0.0236	0	0.004699306	0.00E+00	0.066760709	0.00E+00	1.55E-02	0	2.06E-02	0	1.53E-01	0.047436137	0.33848873	2.53E-02	0	8.77E-02	0.010365747	0	4.31E-03	EGFR_Wt
TCGA-44-2655-01	4.38E-02	0.047860647	0.094550598	0.0403	0	0.280153216	0.00E+00	0.047737215	1.05E-02	0.00E+00	0.001861715	0.00E+00	0.020485828	0.00E+00	0.019930418	0.31339131	5.96E-02	0.007112324	8.09E-03	0.002190362	0	2.47E-03	EGFR_Wt
TCGA-69-7761-01	5.31E-03	0.01275834	0.104419985	0.165	0	0.202862861	2.43E-02	0.039893763	0.00E+00	0.00E+00	0	2.03E-02	0.031865738	6.11E-02	0.099568285	0.16098195	1.45E-02	0.004799747	5.25E-02	0	0	0.00E+00	EGFR_Wt
TCGA-55-6985-01	1.66E-02	0.006596042	0.071786404	0.0956	0	0.170989445	1.89E-02	0.092596351	7.50E-03	0.00E+00	0.014286361	4.83E-02	0.031066894	9.34E-02	0.123130631	0.15006845	9.43E-03	0	2.64E-02	0.023324181	0	0.00E+00	EGFR_Wt
TCGA-NJ-A4YQ-01	2.26E-02	0.053712914	0.050614916	0.154	0	0.062164298	0.00E+00	0.062051938	5.01E-02	1.29E-02	0	3.47E-02	0	2.27E-01	0.087809165	0.1715383	0.00E+00	0	0.00E+00	0.010657117	0	0.00E+00	EGFR_Wt
TCGA-35-3615-01	1.15E-02	0	0.100195335	0.0811	0	0.12557276	0.00E+00	0.050771715	9.46E-03	0.00E+00	0.000742739	3.65E-03	0.013153757	2.75E-02	0.030960272	0.44971619	2.77E-04	0.027475489	5.86E-02	0	0	9.25E-03	EGFR_Wt
TCGA-62-A46R-01	2.12E-02	0.003059858	0	0.0992	0	0.168591338	2.74E-05	0.0356392	3.55E-02	0.00E+00	0	3.44E-02	0.110341285	2.94E-02	0.016418423	0.23651856	3.03E-03	0.061346546	1.45E-01	0	0	0.00E+00	EGFR_Wt
TCGA-55-6975-01	0.00E+00	0.020733596	0.281850398	0.0719	0	0.106426755	0.00E+00	0.054617459	3.11E-03	1.31E-03	0	4.52E-02	0.032235007	1.07E-02	0.002854691	0.21682589	0.00E+00	0.121856757	2.32E-02	0	0	7.19E-03	EGFR_Wt
TCGA-62-A46P-01	1.52E-02	0.03711776	0.097262207	0.0767	0	0.11657827	0.00E+00	0.071443422	2.62E-02	0.00E+00	0	5.79E-02	0.040893299	3.08E-02	0.006255912	0.34589099	0.00E+00	0.039911854	1.43E-02	0.020054511	0	3.49E-03	EGFR_Wt
TCGA-44-6147-01	3.55E-03	0.122083171	0.129695079	0.0808	0	0.155256779	0.00E+00	0.004743948	1.15E-02	1.29E-02	0	0.00E+00	0.003693482	1.14E-01	0.075657979	0.23529909	0.00E+00	0.003765415	4.72E-02	0	0	0.00E+00	EGFR_Wt
TCGA-86-8073-01	2.96E-02	0	0.025463823	0.0561	0	0.097305876	0.00E+00	0.045160742	4.58E-03	0.00E+00	0	2.10E-02	0.114475263	2.77E-02	0.038317316	0.46375159	0.00E+00	0	7.65E-02	0	0	0.00E+00	EGFR_Wt
TCGA-44-2666-01	1.48E-02	0.003297648	0.132099007	0.104	0	0.12676749	0.00E+00	0.027750427	4.56E-04	0.00E+00	0	3.58E-02	0.017508764	2.51E-02	0.004957186	0.17517341	2.82E-02	0.024364101	2.62E-01	0	0.01	6.73E-03	EGFR_Wt
TCGA-91-6848-01	1.04E-03	0	0.0414981	0.0938	0	0.209533942	3.53E-02	0.020077637	0.00E+00	0.00E+00	0	5.23E-02	0.065002485	9.48E-02	0.133409264	0.24527287	3.22E-03	0	0.00E+00	0.003381639	0	1.29E-03	EGFR_Wt
TCGA-78-8662-01	2.45E-02	0	0.147669341	0.104	0	0.074651416	0.00E+00	0.102975695	0.00E+00	0.00E+00	0	7.16E-02	0.010953233	7.74E-02	0.118044012	0.17808961	0.00E+00	0	0.00E+00	0.089942415	0	0.00E+00	EGFR_Wt
TCGA-50-5049-01	1.47E-02	0.018403199	0.067054497	0.184	0	0.124121657	1.18E-02	0.053110761	2.27E-02	4.03E-03	0	4.37E-02	0.02559322	8.83E-02	0.083111414	0.24668391	1.31E-02	0	0.00E+00	0	0	0.00E+00	EGFR_Wt
TCGA-05-5420-01	1.78E-02	0	0.06366055	0.0732	0	0.117992311	1.42E-02	0.043621309	0.00E+00	8.31E-03	0	6.30E-02	0.016722708	5.21E-02	0.150584282	0.27154093	8.72E-02	0	0.00E+00	0.008562425	0	1.14E-02	EGFR_Wt
TCGA-MP-A5C7-01	1.88E-02	0	0.160269433	0.0572	0	0.055199343	0.00E+00	0.110994622	0.00E+00	0.00E+00	0	1.27E-01	0.01208527	3.54E-02	0.038857588	0.26467289	9.29E-03	0.063587137	4.63E-02	0	0	0.00E+00	EGFR_Wt
TCGA-55-8090-01	1.90E-03	0.018847421	0.053026599	0.0458	0	0.178889355	0.00E+00	0.048673063	3.19E-02	0.00E+00	0.004852902	2.79E-03	0.066131198	1.71E-02	0.043916533	0.38023414	5.78E-02	0	0.00E+00	0.038919797	0	9.25E-03	EGFR_Wt
TCGA-49-4505-01	0.00E+00	0.036395911	0.05778163	0.101	0	0.115583716	0.00E+00	0.04841343	2.14E-02	0.00E+00	0	5.31E-02	0.029382513	7.24E-03	0.071096617	0.30053215	5.17E-02	0.034537455	7.01E-02	0	0	1.93E-03	EGFR_Wt

TCGA-93-A4JN-01	3.81E-02	0.057267161	0.148281634	0.0657	0	0.092008139	0.00E+00	0.106585986	5.33E-02	0.00E+00	0	3.41E-02	0.034964083	3.89E-02	0.024052063	0.20364141	0.00E+00	0.014599093	8.86E-02	0	0	0.00E+00	EGFR_Wt
TCGA-55-6972-01	1.32E-02	0	0.340480015	0.161	0	0.038308034	0.00E+00	0.031571323	1.13E-02	0.00E+00	0.031131735	5.31E-02	0.006247894	0.00E+00	0.027374372	0.2095789	0.00E+00	0.073390044	3.10E-03	0	0	0.00E+00	EGFR_Wt
TCGA-97-7553-01	3.04E-02	0.001737625	0	0.11	0	0.127317288	0.00E+00	0.04789681	1.56E-02	0.00E+00	0.02743915	1.09E-02	0.053569775	1.40E-01	0.042135745	0.29749585	1.88E-02	0	1.92E-02	0.023366527	0	3.42E-02	EGFR_Wt
TCGA-55-7994-01	2.34E-02	0.004895771	0.00959245	0.199	0	0.063972841	6.00E-02	0.041211927	6.99E-03	1.09E-02	0	4.70E-02	0.003789167	4.63E-02	0.140519524	0.31078475	1.31E-02	0	0.00E+00	0.016496358	0	2.25E-03	EGFR_Wt
TCGA-69-7763-01	0.00E+00	0.029840136	0.129708385	0.0621	0	0.1761594	0.00E+00	0.066436726	1.93E-02	0.00E+00	0.004512277	1.78E-02	0.00751087	6.85E-02	0.01165764	0.31085948	0.00E+00	0.0459793	3.24E-02	0	0	1.72E-02	EGFR_Wt
TCGA-78-7161-01	0.00E+00	0	0.178366821	0	0	0.262961638	0.00E+00	0.077965259	5.44E-03	0.00E+00	0	8.83E-02	0.033398508	1.05E-01	0.048150115	0.18016959	0.00E+00	0.00801102	8.26E-03	0	0	4.38E-03	EGFR_Wt
TCGA-91-A4BD-01	0.00E+00	0.017078659	0.073081223	0.0311	0	0.066446965	0.00E+00	0.041331794	1.38E-02	0.00E+00	0	3.04E-02	0.21983463	4.05E-03	0.008407518	0.36777866	1.24E-02	0.014382663	9.99E-02	0	0	0.00E+00	EGFR_Wt
TCGA-75-7027-01	7.91E-02	0	0.091665379	0.0702	0	0.118379034	1.24E-02	0.059121739	3.06E-02	0.00E+00	0	2.88E-02	0	1.31E-01	0.062524778	0.29259215	0.00E+00	0	2.38E-02	0	0	0.00E+00	EGFR_Wt
TCGA-78-7154-01	9.54E-02	0	0.039826195	0.0808	0	0.250178736	0.00E+00	0.056809768	0.00E+00	0.00E+00	0	3.72E-02	0.018842287	1.15E-02	0.111635109	0.27460639	4.64E-03	0.000290129	1.78E-02	0	0	4.51E-04	EGFR_Wt
TCGA-78-8648-01	0.00E+00	0.0074205	0.071582695	0.102	0	0.07362746	0.00E+00	0.017903747	1.00E-03	6.96E-03	0	2.48E-02	0	4.15E-01	0.033479117	0.23008946	0.00E+00	0	1.57E-02	0	0	0.00E+00	EGFR_Wt
TCGA-86-8056-01	9.15E-03	0.02637138	0.037717822	0.0492	0	0.202282585	0.00E+00	0.017737935	9.78E-03	0.00E+00	0	3.00E-02	0.024022357	7.38E-02	0.040595174	0.37521532	3.86E-02	0	4.61E-02	0.008061793	0	1.13E-02	EGFR_Wt
TCGA-86-8054-01	6.61E-03	0	0.032234957	0.137	0	0.201238576	0.00E+00	0.033186055	0.00E+00	0.00E+00	0	0.00E+00	0.030135403	4.77E-02	0	0.34538554	2.11E-02	0.050047014	8.41E-02	0	0	1.11E-02	EGFR_Wt
TCGA-99-7458-01	8.04E-03	0.001907941	0	0.117	0	0.274556937	0.00E+00	0.062812744	6.73E-02	0.00E+00	0	2.84E-02	0.029047858	1.99E-02	0.070558545	0.22960467	3.87E-02	0	5.24E-02	0	0	0.00E+00	EGFR_Wt
TCGA-50-5932-01	0.00E+00	0.003666423	0.09017353	0.0482	0	0.167766719	0.00E+00	0.026692496	9.10E-05	0.00E+00	0	3.32E-02	0.049234302	5.72E-02	0.029063399	0.4257803	0.00E+00	0.019533691	4.77E-02	0	0	1.71E-03	EGFR_Wt
TCGA-38-A44F-01	0.00E+00	0.07099164	0.064817376	0.0769	0	0.103158772	0.00E+00	0.095997038	4.42E-02	0.00E+00	0	5.54E-02	0.016747982	3.30E-02	0.08342988	0.25133136	4.61E-02	0	5.59E-02	0	0	2.08E-03	EGFR_Wt
TCGA-73-7498-01	0.00E+00	0.027690127	0.144948667	0.0836	0	0.199723626	0.00E+00	0.037851534	5.10E-02	0.00E+00	0	3.99E-02	0.079838118	0.00E+00	0.019951407	0.20130978	2.38E-03	0.010257739	1.02E-01	0	0	0.00E+00	EGFR_Wt
TCGA-53-A4EZ-01	4.50E-02	0.034168187	0.059400224	0.102	0	0.12362185	0.00E+00	0.13031032	9.33E-03	0.00E+00	0	7.37E-02	0.032169158	0.00E+00	0.068776198	0.22414569	5.62E-02	0	4.05E-02	0	0	9.84E-04	EGFR_Wt
TCGA-73-4676-01	9.11E-04	0	0	0.0595	0	0.07422459	6.00E-02	0.013333684	0.00E+00	0.00E+00	0.008265325	1.38E-02	0.103715653	7.24E-02	0.007491647	0.49092495	0.00E+00	0.018885335	6.82E-02	0	0	8.30E-03	EGFR_Wt
TCGA-49-AAR3-01	0.00E+00	0.02700237	0.01181709	0.193	0	0.017767843	6.21E-02	0.062720468	2.57E-02	0.00E+00	0	1.02E-01	0	1.50E-01	0.086174783	0.25656719	2.97E-03	0	9.29E-04	0	0	0.00E+00	EGFR_Wt
TCGA-78-7148-01	1.78E-02	0	0.082983845	0.178	0	0.152008213	0.00E+00	0.059709408	6.29E-02	0.00E+00	0	4.91E-02	0.029484084	3.68E-02	0.045580977	0.21294996	3.24E-02	0.001583948	3.68E-02	0	0	1.55E-03	EGFR_Wt
TCGA-75-5126-01	0.00E+00	0.038136373	0	0.0698	0	0.049776494	6.14E-02	0.01955111	4.51E-03	0.00E+00	0.005203821	0.00E+00	0.026723131	1.02E-01	0.075483552	0.42365441	1.39E-02	0.029285027	3.05E-03	0.033341159	0	4.15E-02	EGFR_Wt
TCGA-44-A47B-01	1.14E-02	0	0.014799447	0.0845	0	0.11787591	4.09E-03	0.047935565	6.32E-02	0.00E+00	0	8.48E-02	0.007700315	1.58E-01	0.046530965	0.28435926	4.20E-03	0	6.59E-02	0	0	4.31E-03	EGFR_Wt
TCGA-05-5715-01	0.00E+00	0.043643599	0.014825928	0.0242	0	0.083072603	0.00E+00	0.016262819	1.02E-02	0.00E+00	0	8.71E-03	0.001629698	3.00E-01	0.006280481	0.41764989	6.05E-03	0.008133253	5.49E-02	0	0	4.87E-03	EGFR_Wt
TCGA-80-5608-01	2.13E-02	0	0.038308116	0.124	0	0.148106526	0.00E+00	0.072727317	5.37E-02	0.00E+00	0	6.02E-02	0.010327255	5.94E-02	0.055647496	0.29045802	6.27E-03	0	3.18E-02	0	0	2.78E-02	EGFR_Wt
TCGA-93-7348-01	1.63E-02	0.046797255	0.140691076	0.088	0	0.115264194	0.00E+00	0.095024396	4.79E-02	0.00E+00	0	3.95E-02	0	1.70E-01	0.034879455	0.14084681	0.00E+00	0.011597865	0.00E+00	0.045918245	0	7.23E-03	EGFR_Wt
TCGA-4B-A93V-01	0.00E+00	0.009886168	0.173665787	0.117	0	0.027240594	0.00E+00	0.106478666	2.92E-02	0.00E+00	0	6.25E-02	0.010016356	3.18E-01	0.02958333	0.10440233	0.00E+00	0	0.00E+00	0.012490389	0	0.00E+00	EGFR_Wt
TCGA-49-4512-01	3.59E-03	0.058074562	0	0.024	0	0.069590612	0.00E+00	0.043938673	7.73E-02	0.00E+00	0.014105781	6.56E-03	0.040265069	1.04E-01	0.023908097	0.45763512	9.44E-03	0.019892312	4.78E-02	0	0	1.69E-04	EGFR_Wt
TCGA-44-7662-01	2.55E-04	0	0.106332017	0.111	0	0.095573279	0.00E+00	0.042737853	1.78E-02	0.00E+00	0	3.87E-02	0.008204477	1.21E-01	0.082795953	0.31832444	0.00E+00	0	0.00E+00	0.046496022	0	1.08E-02	EGFR_Wt
TCGA-64-1676-01	5.63E-03	0	0.023454531	0.113	0	0.060578417	0.00E+00	0.029312546	1.78E-02	0.00E+00	0	3.99E-02	0.157311854	2.27E-02	0.040128772	0.43067501	3.95E-02	0	4.68E-03	0.015293742	0	0.00E+00	EGFR_Wt
TCGA-78-7536-01	3.12E-03	0	0.083306738	0.115	0	0.109215093	1.00E-02	0.052926725	0.00E+00	0.00E+00	0.013252198	4.07E-02	0.04410985	0.00E+00	0.078587608	0.38551657	9.79E-03	0	0.00E+00	0.049836948	0	5.02E-03	EGFR_Wt
TCGA-44-2668-01	0.00E+00	0.079055972	0	0.0749	0	0.092308833	3.58E-02	0.0011286	9.25E-03	0.00E+00	0.041355462	1.13E-02	0.046597141	2.86E-01	0.079151327	0.10909886	0.00E+00	0	0.00E+00	0.117459618	0	1.67E-02	EGFR_Wt
TCGA-NJ-A55A-01	0.00E+00	0.142116567	0.028122238	0.0871	0	0.21966539	0.00E+00	0.066191836	2.89E-03	0.00E+00	0	4.95E-02	0.015648367	0.00E+00	0.047320318	0.17895367	4.37E-02	0.008288702	1.09E-01	0	0	1.46E-03	EGFR_Wt
TCGA-50-5936-01	1.38E-02	0.005285047	0.039785138	0	0	0.165173084	0.00E+00	0.042796516	3.34E-02	0.00E+00	0.030154812	0.00E+00	0	3.31E-01	0	0.21444667	0.00E+00	0.079774896	0.00E+00	0.035326281	0	8.85E-03	EGFR_Wt
TCGA-55-6986-01	3.28E-03	0.030794403	0.047252751	0.0759	0	0.199322534	0.00E+00	0.003917344	8.63E-04	0.00E+00	0	3.11E-02	0.016831477	2.45E-02	0.019087559	0.41390057	6.41E-02	0	6.61E-02	0	0	3.12E-03	EGFR_Wt
TCGA-05-4389-01	1.62E-02	0	0	0.235	0	0.084129482	3.75E-03	0.060930086	4.32E-02	0.00E+00	0	5.27E-02	0.015693487	4.52E-03	0.116865207	0.24971245	6.75E-02	0	5.01E-02	0	0	0.00E+00	EGFR_Wt
TCGA-78-7537-01	0.00E+00	0.023968878	0.250261258	0.137	0	0.092469799	0.00E+00	0.045430968	6.06E-02	0.00E+00	0	4.19E-02	0.013930478	1.73E-03	0.004162182	0.2357992	2.67E-02	0.021955331	4.38E-02	0	0	0.00E+00	EGFR_Wt

Mutation enrichment

	TCGA-05-4382-01	TCGA-05-4402-01	TCGA-05-4410-01	TCGA-05-5423-01	TCGA-17-Z026-01	TCGA-17-Z032-01	TCGA-17-Z047-01	TCGA-17-Z048-01	TCGA-38-4627-01	TCGA-38-4628-01	TCGA-38-6178-01	TCGA-44-5645-01	TCGA-44-A4SU-01	TCGA-49-4490-01	TCGA-49-4494-01
EGFR	Missense_Mutation	In_Frame_Del	Missense_Mutation	Missense_Mutation	Missense_Mutation	In_Frame_Del	Missense_Mutation	In_Frame_Ins	Missense_Mutation	In_Frame_Del	In_Frame_Del	In_Frame_Ins	Missense_Mutation	Missense_Mutation	Missense_Mutation
TP53	Missense_Mutation	Frame_Shift_Del	Missense_Mutation	Missense_Mutation	Nonsense_Mutation		Splice_Site				Missense_Mutation	Nonsense_Mutation	Nonsense_Mutation	Nonsense_Mutation	
TTN		Missense_Mutation		Silent				Silent	Silent	Intron				Missense_Mutation	
MUC16		In_Frame_Del													
CSMD3	Missense_Mutation	Missense_Mutation	Missense_Mutation		Missense_Mutation	Missense_Mutation									
RYR2	Missense_Mutation		Missense_Mutation										Silent		Missense_Mutation
USH2A	Missense_Mutation		Missense_Mutation	Missense_Mutation	Missense_Mutation								Missense_Mutation		Missense_Mutation
ZFHX4	Missense_Mutation		Missense_Mutation		Missense_Mutation										
SPTA1	Silent	Missense_Mutation	Silent		Missense_Mutation								Silent		
PCDH15	Missense_Mutation		Missense_Mutation		Missense_Mutation										
MUC17	Missense_Mutation	Missense_Mutation	Missense_Mutation										Missense_Mutation		Missense_Mutation
XIRP2	Missense_Mutation		Missense_Mutation		Missense_Mutation										
CSMD1											Missense_Mutation				Missense_Mutation
CDH12	Missense_Mutation		Missense_Mutation		Missense_Mutation										
SYNE2	Missense_Mutation		Nonsense_Mutation		Missense_Mutation	Missense_Mutation									
PAPPA2	Missense_Mutation		Missense_Mutation											Missense_Mutation	
PCLO	Missense_Mutation		Frame_Shift_Del	Missense_Mutation											
PKD1L1	Missense_Mutation		Missense_Mutation	Missense_Mutation	Missense_Mutation					Missense_Mutation				Missense_Mutation	
TNR			Silent												
FLG	Missense_Mutation												Missense_Mutation		

	TCGA-49-4501-01	TCGA-49-6743-01	TCGA-50-5066-01	TCGA-50-5933-01	TCGA-50-5944-01	TCGA-50-6591-01	TCGA-50-6595-01	TCGA-50-6673-01	TCGA-55-6968-01	TCGA-55-6980-01	TCGA-55-6981-01	TCGA-55-7570-01	TCGA-55-7573-01	TCGA-55-8096-01	TCGA-55-8206-01
EGFR	In_Frame_Del	Silent	Splice_Region	Silent	Missense_Mutation	In_Frame_Del	Missense_Mutation	In_Frame_Ins	Missense_Mutation	In_Frame_Del	In_Frame_Del	Missense_Mutation	In_Frame_Del	Missense_Mutation	In_Frame_Del
TP53			Missense_Mutation	Splice_Site	Nonsense_Mutation	Frame_Shift_Del	Nonsense_Mutation	Missense_Mutation	Frame_Shift_Del	Frame_Shift_Del		Missense_Mutation	Missense_Mutation	Nonsense_Mutation	
TTN	Missense_Mutation		Missense_Mutation		Missense_Mutation	Missense_Mutation	Silent	Silent		Missense_Mutation	Missense_Mutation		Missense_Mutation	Intron	Missense_Mutation
MUC16			Missense_Mutation		Missense_Mutation	Missense_Mutation							Nonsense_Mutation		Missense_Mutation
CSMD3		Missense_Mutation	Missense_Mutation	Missense_Mutation	Missense_Mutation							Missense_Mutation		Missense_Mutation	
RYR2		Missense_Mutation		Silent					Missense_Mutation					Missense_Mutation	
USH2A		Silent		Silent					Missense_Mutation		Missense_Mutation				
ZFHX4		Missense_Mutation		Missense_Mutation					Missense_Mutation		Missense_Mutation	Missense_Mutation	Silent	Missense_Mutation	
SPTA1									Missense_Mutation			Splice_Site			
PCDH15				Missense_Mutation								Missense_Mutation		Missense_Mutation	
MUC17			Missense_Mutation	Missense_Mutation		Missense_Mutation									
XIRP2			Missense_Mutation									Missense_Mutation			
CSMD1					Missense_Mutation				Missense_Mutation			Missense_Mutation			
CDH12			Missense_Mutation						Nonsense_Mutation						
SYNE2		Frame_Shift_Ins		Frame_Shift_Del					Missense_Mutation	Missense_Mutation		Missense_Mutation			
PAPPA2			Silent	Missense_Mutation				Missense_Mutation				Missense_Mutation			
PCLO		Nonsense_Mutation		Missense_Mutation								Missense_Mutation			
PKD1L1		Silent											Missense_Mutation		
TNR		Missense_Mutation							Missense_Mutation		Silent	Silent			
FLG		Nonsense_Mutation		Missense_Mutation											

	TCGA-55-8506-01	TCGA-55-A48Z-01	TCGA-55-A57B-01	TCGA-62-8394-01	TCGA-62-8402-01	TCGA-62-A46U-01	TCGA-64-1681-01	TCGA-67-3770-01	TCGA-67-3771-01	TCGA-67-3772-01	TCGA-67-6217-01	TCGA-69-7760-01	TCGA-69-7765-01	TCGA-71-8520-01	TCGA-75-6207-01
EGFR	Missense_Mutation	Missense_Mutation	In_Frame_Del	Missense_Mutation	In_Frame_Del	In_Frame_Del	Missense_Mutation	Missense_Mutation	Silent	Missense_Mutation	Missense_Mutation	In_Frame_Del	Nonsense_Mutation	Missense_Mutation	In_Frame_Del
TP53	Missense_Mutation	Missense_Mutation	Missense_Mutation	Missense_Mutation	Missense_Mutation	Missense_Mutation	Missense_Mutation	Missense_Mutation	Missense_Mutation			Missense_Mutation	Nonsense_Mutation	Frame_Shift_Del	Nonsense_Mutation
TTN	Missense_Mutation				Silent		Missense_Mutation	Missense_Mutation	Frame_Shift_Del		Missense_Mutation	Missense_Mutation			
MUC16	In_Frame_Ins						Missense_Mutation	Missense_Mutation				Nonsense_Mutation			
CSMD3								Missense_Mutation	Missense_Mutation				Missense_Mutation		
RYR2	Missense_Mutation				Nonsense_Mutation			Missense_Mutation	Missense_Mutation	Missense_Mutation		Missense_Mutation	Missense_Mutation	Missense_Mutation	
USH2A	Missense_Mutation								Missense_Mutation		Missense_Mutation		Silent		
ZFHX4	Silent								Missense_Mutation				Missense_Mutation		
SPTA1	Missense_Mutation							Frame_Shift_Del			Missense_Mutation		Missense_Mutation		
PCDH15	Missense_Mutation						Missense_Mutation						Missense_Mutation		
MUC17	Silent							Missense_Mutation			Missense_Mutation		Silent		
XIRP2	Missense_Mutation			Missense_Mutation					Missense_Mutation						Missense_Mutation
CSMD1	Missense_Mutation								Frame_Shift_Del		Missense_Mutation	Silent			
CDH12	Missense_Mutation							Missense_Mutation	Missense_Mutation		Missense_Mutation				
SYNE2	Missense_Mutation							In_Frame_Del	Frame_Shift_Del						
PAPPA2	Missense_Mutation														
PCLO	Frame_Shift_Del							Missense_Mutation					Silent		Missense_Mutation
PKD1L1									Silent						
TNR	Frame_Shift_Ins							Missense_Mutation					Missense_Mutation		
FLG	Missense_Mutation								Missense_Mutation		Missense_Mutation		Missense_Mutation		

	TCGA-75-6212-01	TCGA-75-7025-01	TCGA-78-7147-01	TCGA-78-7155-01	TCGA-78-7158-01	TCGA-86-8055-01	TCGA-86-8074-01	TCGA-86-8075-01	TCGA-86-8280-01	TCGA-86-8668-01	TCGA-86-A4JF-01	TCGA-86-A4P7-01	TCGA-91-6835-01	TCGA-93-A4JP-01	TCGA-95-7039-01
EGFR	In_Frame_Del	In_Frame_Del	Missense_Mutation	Missense_Mutation	Missense_Mutation	Missense_Mutation	In_Frame_Del	Missense_Mutation	In_Frame_Del	Missense_Mutation	Intron	In_Frame_Del	Missense_Mutation	Missense_Mutation	Missense_Mutation
TP53			Nonsense_Mutation	Missense_Mutation		Missense_Mutation	Missense_Mutation	Splice_Site	Missense_Mutation		In_Frame_Del	Missense_Mutation		Nonsense_Mutation	Missense_Mutation
TTN		Missense_Mutation	Missense_Mutation	Missense_Mutation	Missense_Mutation					Missense_Mutation				Missense_Mutation	Missense_Mutation
MUC16			Missense_Mutation	Silent						Missense_Mutation					Missense_Mutation
CSMD3				Intron	Splice_Site		Missense_Mutation				Missense_Mutation		Missense_Mutation		Missense_Mutation
RYR2			Missense_Mutation	Silent			Silent				Missense_Mutation				Silent
USH2A				Missense_Mutation	Missense_Mutation						Missense_Mutation				
ZFHX4			Missense_Mutation	Missense_Mutation	Missense_Mutation						Missense_Mutation				Missense_Mutation
SPTA1			Nonsense_Mutation	Missense_Mutation							Missense_Mutation				Silent
PCDH15				Missense_Mutation							Missense_Mutation				Missense_Mutation
MUC17			Missense_Mutation	Missense_Mutation											Missense_Mutation
XIRP2				Missense_Mutation							Silent				Silent
CSMD1			Missense_Mutation	Missense_Mutation	Missense_Mutation	Missense_Mutation					Silent				Missense_Mutation
CDH12					Missense_Mutation						Missense_Mutation				Missense_Mutation
SYNE2				Silent											Missense_Mutation
PAPPA2				Missense_Mutation							Silent				Missense_Mutation
PCLO															Missense_Mutation
PKD1L1			Missense_Mutation	Missense_Mutation											Missense_Mutation
TNR				Silent							Missense_Mutation		Missense_Mutation		Missense_Mutation
FLG			Missense_Mutation	Missense_Mutation			Silent				Missense_Mutation				Missense_Mutation

	TCGA-95-7947-01	TCGA-97-8171-01	TCGA-97-8172-01	TCGA-97-8177-01	TCGA-97-8547-01	TCGA-97-8552-01	TCGA-97-A4M1-01	TCGA-97-A4M6-01	TCGA-97-A4M7-01	TCGA-J2-8192-01	TCGA-L9-A50W-01	TCGA-MP-A4SW-01	TCGA-MP-A4T6-01	TCGA-MP-A4T9-01
EGFR	Missense_Mutation	In_Frame_Del	Nonsense_Mutation	Missense_Mutation	In_Frame_Del	In_Frame_Del	Missense_Mutation	In_Frame_Del	Missense_Mutation	In_Frame_Del	Missense_Mutation	Missense_Mutation	In_Frame_Del	Missense_Mutation
TP53	Missense_Mutation		Splice_Region		Splice_Site		In_Frame_Del							Splice_Region
TTN						Missense_Mutation			Silent	Missense_Mutation	Missense_Mutation			
MUC16						Missense_Mutation					Intron			
CSMD3		Missense_Mutation			Frame_Shift_Ins				Missense_Mutation		Missense_Mutation			
RYR2	Silent		Missense_Mutation				Missense_Mutation							
USH2A	Silent								Missense_Mutation					
ZFHX4	Missense_Mutation													
SPTA1		Missense_Mutation	Splice_Site											
PCDH15	Missense_Mutation		Missense_Mutation						Missense_Mutation					
MUC17														
XIRP2			Missense_Mutation				Missense_Mutation		Silent					
CSMD1			Silent											
CDH12			Missense_Mutation											
SYNE2	Missense_Mutation													
PAPPA2	Missense_Mutation			Missense_Mutation					Silent			Missense_Mutation		
PCLO			Missense_Mutation		Missense_Mutation							Missense_Mutation		
PKD1L1					Missense_Mutation									
TNR	Missense_Mutation						Missense_Mutation		Frame_Shift_Ins				Missense_Mutation	
FLG									Missense_Mutation				Missense_Mutation	

GSE31210

Accession	EGFR status	YAP1	CD274
GSM773639	EGFR mutation +	12.795492	11.13795897
GSM773640	EGFR mutation +	13.01457742	10.49101368
GSM773641	EGFR mutation +	12.71321122	11.80146894
GSM773642	EGFR mutation +	12.68081266	9.848954315
GSM773643	EGFR mutation +	12.64922878	10.48660809
GSM773644	EGFR mutation +	12.84546378	10.34026238
GSM773645	EGFR mutation +	13.01287695	9.606762742
GSM773646	EGFR mutation +	13.47014624	9.113363995
GSM773647	EGFR mutation +	12.90948999	9.813399628
GSM773648	EGFR mutation +	12.55405278	11.17201152
GSM773649	EGFR mutation +	12.24701872	8.929799577
GSM773650	EGFR mutation +	12.72150254	11.24651777
GSM773651	EGFR mutation +	12.77599189	9.275454094
GSM773652	EGFR mutation +	12.66641798	11.70930429
GSM773653	EGFR mutation +	12.43211461	10.00355853
GSM773654	EGFR mutation +	12.50060216	8.52488134
GSM773655	EGFR mutation +	13.01895124	9.995155324
GSM773656	EGFR mutation +	12.62121397	10.41279825
GSM773657	EGFR mutation +	12.66052276	10.94733361
GSM773658	EGFR mutation +	12.77727504	9.700820456
GSM773659	EGFR mutation +	12.83064295	9.008821318
GSM773660	EGFR mutation +	12.88183723	9.933772081
GSM773661	EGFR mutation +	12.98237973	9.523408662
GSM773662	EGFR mutation +	13.04743393	9.146847387
GSM773663	EGFR mutation +	13.34603192	10.05099558
GSM773664	EGFR mutation +	13.45912727	10.10345909
GSM773665	EGFR mutation +	10.8196626	9.031147311
GSM773666	EGFR mutation +	11.77005101	10.05890969
GSM773667	EGFR mutation +	12.64847129	11.05847845
GSM773668	EGFR mutation +	12.77856815	9.940435175
GSM773669	EGFR mutation +	13.16818477	9.757342169
GSM773670	EGFR mutation +	12.56685914	9.404176365
GSM773671	EGFR mutation +	13.04128269	11.89436041
GSM773672	EGFR mutation +	12.85806358	10.12788034
GSM773673	EGFR mutation +	13.06750377	10.94178498
GSM773674	EGFR mutation +	12.85920669	10.97349748
GSM773675	EGFR mutation +	13.72855341	9.072532188
GSM773676	EGFR mutation +	13.28066045	9.788046332
GSM773677	EGFR mutation +	12.95969536	10.52902092
GSM773678	EGFR mutation +	13.10933819	9.340491726
GSM773679	EGFR mutation +	12.99080758	10.67417779
GSM773680	EGFR mutation +	12.78009024	11.1061283
GSM773681	EGFR mutation +	13.06625488	9.107926085
GSM773682	EGFR mutation +	12.24065928	8.063739669
GSM773683	EGFR mutation +	12.22120555	11.04411963
GSM773684	EGFR mutation +	12.98227561	9.273427304
GSM773685	EGFR mutation +	13.38393887	9.30970121
GSM773686	EGFR mutation +	13.55505425	8.976250205
GSM773687	EGFR mutation +	13.1908099	10.02819382
GSM773688	EGFR mutation +	13.46745513	11.79217014
GSM773689	EGFR mutation +	13.30491041	10.72670914
GSM773690	EGFR mutation +	13.44013868	9.871277829
GSM773691	EGFR mutation +	13.32140953	9.125224887
GSM773692	EGFR mutation +	13.0365454	11.29760115

GSM773693	EGFR mutation +	12.90790783	9.521826739
GSM773694	EGFR mutation +	13.17074927	8.557423493
GSM773695	EGFR mutation +	12.86546322	10.66722493
GSM773696	EGFR mutation +	13.01641118	10.04100842
GSM773697	EGFR mutation +	12.64757108	11.27424949
GSM773698	EGFR mutation +	13.44148568	8.619797404
GSM773699	EGFR mutation +	13.58652188	9.247709669
GSM773700	EGFR mutation +	13.336968	9.990337301
GSM773701	EGFR mutation +	13.30462512	9.938807269
GSM773702	EGFR mutation +	13.11288889	9.995022862
GSM773703	EGFR mutation +	12.54039252	9.63114922
GSM773704	EGFR mutation +	13.00365757	9.524568159
GSM773705	EGFR mutation +	13.32009962	9.462473848
GSM773706	EGFR mutation +	13.42635217	9.022537875
GSM773707	EGFR mutation +	13.08763737	9.299953218
GSM773708	EGFR mutation +	12.65727289	8.904035745
GSM773709	EGFR mutation +	13.26226988	9.220665387
GSM773710	EGFR mutation +	12.64981078	9.844858734
GSM773711	EGFR mutation +	12.78549089	9.812475603
GSM773712	EGFR mutation +	12.69857619	9.297916343
GSM773713	EGFR mutation +	12.83888954	10.34390544
GSM773714	EGFR mutation +	12.86338931	10.08793853
GSM773715	EGFR mutation +	12.85579256	10.2910323
GSM773716	EGFR mutation +	12.59336824	10.72410307
GSM773717	EGFR mutation +	13.33069032	9.407581341
GSM773718	EGFR mutation +	12.70066295	8.838473121
GSM773719	EGFR mutation +	13.26765123	8.963492042
GSM773720	EGFR mutation +	12.75093095	9.538882074
GSM773721	EGFR mutation +	12.93374616	11.53379201
GSM773722	EGFR mutation +	13.42302567	9.426974185
GSM773723	EGFR mutation +	12.74631445	10.5203143
GSM773724	EGFR mutation +	13.01674614	9.744596395
GSM773725	EGFR mutation +	12.65506264	9.662902605
GSM773726	EGFR mutation +	12.90336842	11.85370589
GSM773727	EGFR mutation +	13.54490528	9.506672945
GSM773728	EGFR mutation +	13.02921509	9.886767169
GSM773729	EGFR mutation +	13.05656101	9.563864163
GSM773730	EGFR mutation +	13.18807726	9.314697658
GSM773731	EGFR mutation +	12.96393392	10.03457161
GSM773732	EGFR mutation +	12.58564776	10.91629077
GSM773733	EGFR mutation +	13.1994081	9.693584354
GSM773734	EGFR mutation +	13.43359939	10.42687012
GSM773735	EGFR mutation +	13.55549061	9.387155071
GSM773736	EGFR mutation +	12.56536536	9.630462809
GSM773737	EGFR mutation +	13.22174781	9.374069414
GSM773738	EGFR mutation +	12.90682503	9.84896245
GSM773739	EGFR mutation +	12.94654044	10.20638706
GSM773740	EGFR mutation +	13.18126021	8.953877777
GSM773741	EGFR mutation +	12.96222437	11.64786748
GSM773742	EGFR mutation +	13.01380628	9.571906005
GSM773743	EGFR mutation +	12.80625316	10.62660906
GSM773744	EGFR mutation +	12.4835188	10.55395168
GSM773745	EGFR mutation +	13.07313872	9.216266173
GSM773746	EGFR mutation +	12.63705116	10.55718959
GSM773747	EGFR mutation +	12.80162976	9.251993595
GSM773748	EGFR mutation +	13.29373071	9.940933125
GSM773749	EGFR mutation +	13.17743387	8.673679729
GSM773750	EGFR mutation +	12.95026992	9.200145314

GSM773751	EGFR mutation +	13.31863022	9.325298034
GSM773752	EGFR mutation +	13.48604622	9.609796323
GSM773753	EGFR mutation +	13.34098473	9.256159615
GSM773754	EGFR mutation +	13.25169631	9.529358515
GSM773755	EGFR mutation +	13.45550421	9.210371817
GSM773756	EGFR mutation +	13.64469125	9.388459716
GSM773757	EGFR mutation +	13.52070424	9.834910586
GSM773758	EGFR mutation +	13.42186904	9.050942949
GSM773759	EGFR mutation +	13.05294617	10.12467075
GSM773760	EGFR mutation +	13.34862526	9.16670189
GSM773761	EGFR mutation +	13.0943174	9.569038546
GSM773762	EGFR mutation +	13.53045828	9.474932026
GSM773763	EGFR mutation +	13.54685661	9.567219904
GSM773764	EGFR mutation +	12.87296839	10.77107121
GSM773765	EGFR mutation +	12.88250321	9.625698616

GO_KEGG

ONTOLOGY	ID	Description	GeneRatio	BgRatio	p-value	p.adjust	q-value	GeneID	Count
BP	GO:0016570	histone modification	62/840	494/18800	1.70177E-13	8.06811E-10	6.92891E-10	KMT2A/ASXL1/TRIP12/EP300/RNF20/NCOA3/HUWE1/RIF1/REST/KDM5B/TAF1/MCM3AP/KDM2A/TET3/EYA3/RNF168/KMT2E/ELK4/BRPF3/JARID2/RNF40/EHMT1/CHD3/CREBBP/KMT2D/SETD1B/CHD4/BAZ2A/BCOR/MIDEAS/NIPBL/ASH1L/NSD1/HDAC4/YEATS2/SUPT6H/HCFC1/KAT6B/KMT2C/ATRX/GTF3C4/USP36/TRRAP/TAF4/BAZ1B/ATM/PHF8/MLLT6/EP400/PER2/UBR5/SKI/SETD5/MLLT3/MTHFR/MYSM1/PRKAA2/FBXL19/KDM6B/AASS/DNMT3B/PADI2	62
BP	GO:0016055	Wnt signaling pathway	43/840	452/18800	2.67851E-06	0.000552122	0.000474163	BCL9L/CTNND1/CHD8/TNKS/USP34/LRP6/FOXO3/FZD5/CELSR1/NOTCH1/DAB2IP/KANK1/ABL1/LRP5/MED12/MACF1/BCL9/SMURF1/JRK/UBR5/SKI/PTK7/FAM53B/MLLT3/PRKAA2/WNK1/AMOTL2/PKD1/ROR1/AXIN2/LRP1/RNF43/NKD1/FZD4/PRICKLE1/SOX13/AMOTL1/PTPRU/RNF213/GLI3/LRP4/DCDC2/CCND1	43
BP	GO:0035329	hippo signaling	10/840	40/18800	7.51044E-06	0.000962351	0.000826469	MOB1B/TEAD3/SHANK2/NF2/AJUBA/DLG5/AMOTL2/AMOT/AMOTL1/DCHS1	10
BP	GO:0000209	protein polyubiquitination	25/840	244/18800	0.000101018	0.006301676	0.005411891	UBE4A/TRIP12/TNKS/TTC3/RNF20/HECTD1/CBL/HUWE1/TAF1/AREL1/RNF168/RMND5A/ANAPC1/RNF26/RNF40/SASH1/RC3H2/MARCHF6/PPIL2/UBE4B/TPP2/SMURF1/TRIM56/UBR5/RNF213	25
BP	GO:0060071	Wnt signaling pathway, planar cell polarity pathway	9/840	53/18800	0.000513689	0.018039997	0.01549278	CELSR1/ABL1/MED12/SMURF1/PTK7/MLLT3/ROR1/NKD1/PRICKLE1	9
CC	GO:0005911	cell-cell junction	49/863	497/19594	1.23028E-07	7.02488E-05	5.72402E-05	CTNND1/UBN1/SPECC1L/PKP4/TLN1/TJP1/PPL/FZD5/RAPGEF2/PIKFYVE/WASF2/NF2/NOTCH1/ASH1L/PARD3B/CDC42BPB/PATJ/AJUBA/MPDZ/TJP2/TNKS1BP1/MYH9/PTK7/CDC42BPA/PODXL/AHNAK/PNN/AMOTL2/SHROOM3/MAGI3/AMOT/SPTBN2/DSP/PDZD2/FLNA/FZD4/SHROOM4/ARVCF/AMOTL1/PTPRU/NECTIN1/CGNL1/KIRREL1/DSC2/DCHS1/TRPC6/CGN/HMCN1/CCND1	49
CC	GO:0035770	ribonucleoprotein granule	28/863	261/19594	1.33322E-05	0.000761267	0.000620297	DDX6/GIGYF2/AGO1/KHNYN/PUM1/ROCK2/UBAP2/TNRC6B/UPF2/AGO3/EIF4G1/NCL/TUT4/CNOT1/PMS2/RC3H2/TRIM25/ZNFX1/AJUBA/PRRC2C/NSUN2/LARP1/UPF1/AGO2/TNRC6A/PRKAA2/SMN2/NYNRIN	28
CC	GO:0005923	bicellular tight junction	16/863	124/19594	0.000110384	0.003707607	0.003021038	UBN1/TJP1/FZD5/RAPGEF2/ASH1L/PARD3B/PATJ/MPDZ/TJP2/AMOTL2/MAGI3/AMOT/AMOTL1/CGNL1/CGN/CCND1	16
MF	GO:0042393	histone binding	41/843	250/18410	1.00264E-12	6.23633E-10	5.24664E-10	KMT2A/INO80/CHD8/TNKS/RNF20/KDM5B/TAF1/MCM3AP/CHD6/TP53BP1/RNF168/KMT2E/JARID2/SBNO1/PHC3/CHD3/BPTF/KMT2D/CHD4/CHD2/BAZ2A/ZZEF1/HIRA/YEATS2/SUPT6H/KAT6B/KMT2C/BRD2/ATRX/BRD3/BAZ1B/PHF8/MLLT6/NCAPD3/MLLT3/MYSM1/NCAPD2/NASP/CHD7/AASS/TSPYL5	41
MF	GO:0140297	DNA-binding transcription factor binding	50/843	470/18410	2.6755E-08	4.21124E-06	3.54292E-06	THRAP3/ASXL1/HIF1AN/RBFOX2/TRIP12/EP300/TOB2/NCOA3/REST/TEAD3/TAF1/NCL/SPEN/NCOR1/TP53BP1/UBXN7/HIPK2/CNOT1/MED1/CREBBP/PDCD11/GTF2I/RXRA/CHD4/NOTCH2/BAZ2A/BCOR/NSD1/HDAC4/HIRA/HCFC1/KAT6B/NCOA6/MED12/PPARA/TAF4/DNMT3A/SKI/ARID1A/PBX2/SMARCA1/DHX33/PRKDC/NCOR2/FLNA/NFIA/FOXC1/TRIB2/CENPF/PADI2	50
MF	GO:0042813	Wnt-activated receptor activity	6/843	15/18410	3.17508E-05	0.001388216	0.00116791	LRP6/FZD5/LRP5/PKD1/ROR1/FZD4	6
KEGG	hsa04512	ECM-receptor interaction	16/357	88/8164	1.03892E-06	0.000267002	0.000225281	LAMC1/LAMA5/HSPG2/AGRN/FRAS1/VWF/COL4A2/ITGA9/LAMB1/ITGA6/COL4A1/FREM2/COL4A5/ITGA8/TNXB/LAMA3	16
KEGG	hsa04330	Notch signaling pathway	10/357	59/8164	0.000208281	0.007437013	0.006274932	ATXN1L/EP300/NOTCH1/CREBBP/NOTCH2/MAML3/NOTCH3/NCOR2/MAML2/DTX4	10
KEGG	hsa04151	PI3K-Akt signaling pathway	28/357	354/8164	0.001648311	0.032585841	0.027494094	PPP2R1B/SOS1/FOXO3/LAMC1/IGF1R/MTOR/RXRA/LAMA5/INSR/TSC1/PRKAA2/PHLPP1/VWF/COL4A2/PIK3R3/FLT1/ITGA9/LAMB1/ITGA6/COL4A1/CDK6/COL4A5/IRS1/ITGA8/TNXB/LAMA3/CCND1/LPAR3	28
KEGG	hsa04152	AMPK signaling pathway	12/357	121/8164	0.00649778	0.07260563	0.061260535	PPP2R1B/FOXO3/IGF1R/MTOR/ACACA/INSR/TSC1/FASN/PRKAA2/PIK3R3/IRS1/CCND1	12
KEGG	hsa04310	Wnt signaling pathway	15/357	170/8164	0.007409394	0.079342258	0.066944522	CHD8/ROCK2/EP300/LRP6/FZD5/CREBBP/LRP5/PLCB3/ROR1/AXIN2/RNF43/NKD1/FZD4/PRICKLE1/CCND1	15

REN_immunotherapy_DATA

Sample ID	OS (months)	OS Status	PFS (months)	PFS Status	YAP1
M19D8102V1R1	28.1	0	7.2	0	16.34
M19D7753V1R1	24	0	23.7	1	57.48
M19D7754V1R1	13.8	1	4.4	1	62.63
M19D8105V1R1	24.4	0	21.8	1	39.64
M19D8111V1R1	22.2	0	4	1	59.6
M19D8112V1R1	7	0	1	1	29.47
M19D7731V1R1	15.6	1	5.8	1	89.65
M19D7740V1R1	5.3	1	1.6	1	96.12
M19D8110V1R1	21.4	0	13.1	1	86.48
M19D7729V1R1	17.7	0	8.6	1	139.94
M19D7737V1R1	18	1	5.1	1	68.63
M19D7744V1R1	17.3	1	1.4	1	132.4
M19D7748V1R1	2.7	0	2.7	1	110.64
M19D8114V1R1	15.2	0	0.1	0	46.07
M19D7735V1R1	18.9	0	5.6	1	134.36
M19D7752V1R1	22.2	0	7	1	112.15
M19D7734V1R1	6.4	1	2.8	1	121.97
M19D7746V1R1	24.6	0	8.3	1	70.9

GSE135222

Gene_id	PFS (months)	PFS STATUS	RESPONSE	YAP1
NSCLC1327	6.8	0	DCB	45.07
NSCLC1352	8.3	0	DCB	61.69
NSCLC1358	8.6	1	DCB	12.46
NSCLC1510	10.8	0	DCB	57.16
NSCLC1528	9.3	0	DCB	17.56
NSCLC1708	5.6	1	DCB	17.93
NSCLC378	13.7	0	DCB	97.94
NSCLC947	20.6	0	DCB	4.68
NSCLC1017	1.3	1	NDB	14.52
NSCLC1066	0.8	1	NDB	113.88
NSCLC1079	5.8	1	NDB	8.62
NSCLC1104	1.1	1	NDB	52.71
NSCLC1145	2.4	1	NDB	11.5
NSCLC1155	1.1	1	NDB	83.71
NSCLC1164	0.4	1	NDB	37.42
NSCLC1203	0.8	1	NDB	38.07
NSCLC1401	0.9	1	NDB	31.22
NSCLC1412	0.1	1	NDB	64.34
NSCLC1425	2	1	NDB	59.71
NSCLC1508	2.2	1	NDB	44.81
NSCLC1554	1.2	1	NDB	19
NSCLC1619	2.7	1	NDB	76.63
NSCLC1809	1.5	1	NDB	38.46
NSCLC1873	1.2	1	NDB	22.44
NSCLC573	1.5	1	NDB	54.63
NSCLC825	1.4	1	NDB	44.92
NSCLC990	3.2	1	NDB	6.95
